# Hybrid Gold(I) NHC-Artemether Complexes to Target Falciparum Malaria Parasites

**DOI:** 10.3390/molecules25122817

**Published:** 2020-06-18

**Authors:** Manel Ouji, Guillaume Barnoin, Álvaro Fernández Álvarez, Jean-Michel Augereau, Catherine Hemmert, Françoise Benoit-Vical, Heinz Gornitzka

**Affiliations:** 1 CNRS, Laboratoire de Chimie de Coordination (LCC), Université de Toulouse, UPS, INPT, 205 route de Narbonne, BP 44099, F-31077 Toulouse CEDEX 4, France; manel.ouji@lcc-toulouse.fr (M.O.); guillaume.barnoin@gmail.com (G.B.); alvaritocanada@yahoo.es (Á.F.Á.); jean-michel.augereau@lcc-toulouse.fr (J.-M.A.); 2 Institut de Pharmacologie et de Biologie Structurale, IPBS, Université de Toulouse, CNRS, UPS, 31077 Toulouse, France; 3 INSERM, Institut National de la Santé et de la Recherche Médicale, 31024 Toulouse, France

**Keywords:** malaria, *Plasmodium falciparum*, gold, NHC-ligands, hybrid molecules, drug resistance

## Abstract

The emergence of *Plasmodium falciparum* parasites, responsible for malaria disease, resistant to antiplasmodial drugs including the artemisinins, represents a major threat to public health. Therefore, the development of new antimalarial drugs or combinations is urgently required. In this context, several hybrid molecules combining a dihydroartemisinin derivative and gold(I) N-heterocyclic carbene (NHC) complexes have been synthesized based on the different modes of action of the two compounds. The antiplasmodial activity of these molecules was assessed in vitro as well as their cytotoxicity against mammalian cells. All the hybrid molecules tested showed efficacy against *P. falciparum*, in a nanomolar range for the most active, associated with a low cytotoxicity. However, cross-resistance between artemisinin and these hybrid molecules was evidenced. These results underline a fear about the risk of cross-resistance between artemisinins and new antimalarial drugs based on an endoperoxide part. This study thus raises concerns about the use of such molecules in future therapeutic malaria policies.

## 1. Introduction

*Plasmodium falciparum*, the protozoan parasite causing malaria, was responsible for 228 million cases with 405,000 deaths in 2018, mainly in Sub-Saharan Africa where about 90% of the deaths affect children under five [1]. The current malaria treatments recommended by the WHO are artemisinin-based combination therapies (ACTs) combining an artemisinin derivative with one or two other antimalarial drugs with the particularity of having different modes of action and different pharmacokinetic properties. The use of these drug combinations has contributed to a significant decrease in malaria mortality in all endemic regions these last 20 years. However, since 2008, resistance of *P. falciparum* to artemisinins and partner drugs has widely spread in South-East Asia, resulting in a loss of efficacy of many antimalarial drugs to treat patients [2,3,4]. This situation is a major threat to global public health [5] and imposes a need to accelerate the discovery of new compounds able to eliminate resistant parasites. Among the strategies followed to improve the efficacy of artemisinin both directly against the parasite or for pharmacokinetic and pharmacodynamic enhancements, the synthesis of artemisinin dimers is proposed [6,7,8]. One of the aims was to amplify oxidative stress through increased production of reactive oxygen species (ROS) to kill the parasites. However, although some of these compounds show antiplasmodial activities in the nanomolar range, they have not been evaluated in an artemisinin resistance framework needing particular assays. Indeed, because artemisinin resistance is based on a quiescence mechanism corresponding to a cell cycle arrest of a small number of parasites during artemisinins treatment and resumption of their growth when the drug is eliminated [9,10,11], specific compounds have to be designed. Artemisinin resistance is also associated with mutations in the propeller domain of the gene *pfk13* linked to a complex combination of different biochemical pathways [10,12,13]. It is to note that, at the quiescent state, the parasite’s metabolism is greatly downregulated. However, apicoplast and mitochondrion seem still active in quiescent parasites [14]. Targeting these two organelles appears thus as a very promising avenue to kill quiescent artemisinin-resistant parasites. In this context, we have recently synthetized and evaluated hybrid gold(I) *N*-heterocyclic carbene (NHC) complexes based on triclosan targeting mitochondrion and apicoplast, respectively [12]. These novel hybrids showed a strong antiplasmodial activity, however a cross-resistance trend with artemisinins was noted [12]. Interestingly, we showed that there is no cross-resistance between a gold(I) complex and artemisinins [12]. This is in accordance with former studies showing that atovaquone, a mitochondrial electron transfer inhibitor, was efficient not only on proliferating parasites but also on artemisinin-pretreated and dormant parasites [15,16].

Here, we focused our investigations on the second organelle involved in the quiescence mechanism: the parasite mitochondrion. In addition to its role as an energy supplier, mitochondrion contains a wide variety of additional processes notably the two major antioxidant defence systems: thioredoxin (Trx) and glutathione systems, Trx being essential for the erythrocytic *P. falciparum* cycle [17].

Our chemical research is mainly focused on the design of bioactive gold(I) NHC complexes for parasitic diseases, *P. falciparum* [18,19,20] and *Leishmania infantum* [21,22], and anticancer applications [23,24,25]. Moreover, in the field of anticancer metal-based agents, the ubiquitous selenoenzyme thioredoxin reductase (TrxR), responsible for cell homeostasis regulation, is considered as one of the most relevant targets for gold(I) complexes and inhibition of TrxR could lead to apoptosis though a mitochondrial pathway [24,25,26]. Considering that artemisinins—and dihydroartemisinin, the active metabolite of all artemisinin derivatives—are active against all erythrocytic stages of *P. falciparum* and are still the best antiplasmodial drugs on the field, we designed hybrid complexes combining an ether derivative of dihydroartemisinin (called here DHA) with a gold(I) cation, covalently integrated in our NHC ligand systems. This approach aims to firstly eliminate most of the parasite stages thanks to the highly active DHA part, thereafter, the remaining resistant quiescent parasites will be treated by the gold(I) moiety. The goal of this work is to determine the activity of these hybrid molecules against *P. falciparum*, then to evaluate the efficacy of the most active hybrids in a context of artemisinin resistance.

## 2. Results and Discussion

### 2.1. Chemistry

**Scheme 1:**
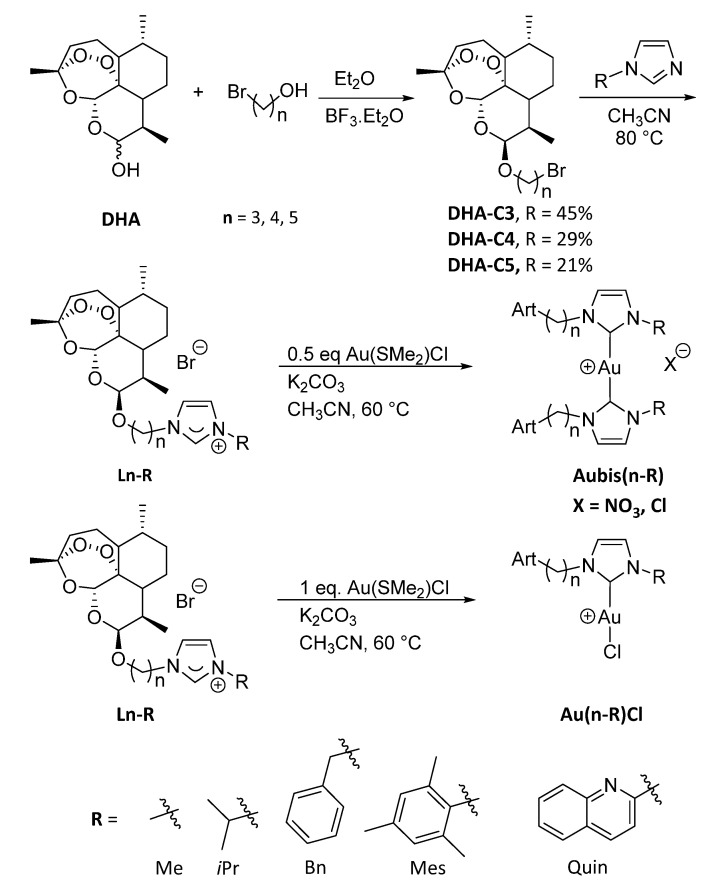
Synthesis of proligands **Ln-R** (**1**–**13**) and gold(I) complexes **Aubis(n-R)** and **Au(n-R)Cl** complexes (**14**–**29**).

The gold(I) complexes designed here are divided in three series depending on the length of the spacers between the carbene and the pharmacophore derivative. In order to fuse DHA and NHCs precursors we used aliphatic linkers containing 3 to 5 carbon atoms. The synthetic pathway involves three steps (Scheme 1). First, etherification of DHA with a bromoalcohol in the presence of boron trifluoride etherate catalyst [27,28] gave after purification the single β-isomers **DHA-C3** to **DHA-C5**. The next step was the quaternization of a substituted imidazole, either commercially available (Me, *i*Pr and Bn) or previously described in the literature (Mes, Quin) [22,29], to obtain the corresponding carbene precursors **Ln-R**(**1**–**13**) in yields ranging from 35 to 98%. They were classically characterized by ^1^H- and ^13^C-NMR spectroscopy, mass spectrometry and elemental analysis. The most notable features in the ^1^H- and ^13^C-NMR spectra of the imidazolium salts are the resonances for the imidazolium protons (H2) located between 10.07 and 12.15 ppm, the upfield value being attributed to the proligands containing a quinoline group and the corresponding imidazolium carbons (C2) in the range of 135.7–138.7 ppm. The formation of the target gold(I) complexes was achieved by direct metalation involving K_2_CO_3_ as base and **Au(SMe_2_)Cl**, in a ratio 2:1 for the cationic **Aubis(n-R)** complexes **15**–**26** and in a ratio 1:1 for the neutral **Au(n-R)Cl** ones (**27** and **28**). Complexes **Aubis(L3-Me)** (**14**), was synthetized by the convenient transmetalation route involving the mild base Ag_2_O, followed by an ion exchange with AgNO_3_ and subsequent addition of **Au(SMe_2_)Cl** [28]. The neutral complex **Au(3-Quin)Cl** (**29**) was obtained as a byproduct from the purification by column chromatography of **Aubis(3-Quin)** (**18**). The gold(I) complexes **15**–**26** were isolated after purification as white solids with yields of 31 to 92%. All compounds were characterized by ^1^H- and ^13^C-NMR spectroscopy, high-resolution mass spectrometry and elemental analysis. ^13^C-NMR spectroscopy unequivocally evidences the formation of the cationic gold(I) complexes **Aubis(n-R)** (**15**–**26**) with resonance of the carbenic carbons located at 181.8-183.9 ppm. In the case of mono-NHC complexes **Au(n-R)Cl** (**27**–**29**), the most characteristic features in ^13^C NMR spectra are the C2 peaks at 173.3, 174.5 and 173.7 ppm for **Au(3-*i*Pr)Cl** (**27**), **Au(3-Bn)Cl** (**28**) and **Au(3-Quin)Cl** (**29**), respectively. The elemental analysis for the gold(I) complexes correspond to the general formula [AuL_2_][Cl] (except for **Aubis(L3-Me)** (**14**) with nitrate anion) for the bis(NHC) complexes **Aubis(n-R)** (**15**–**26**) and **AuLCl** for the mono(NHC) complexes **Au(n-R)Cl** (**27**–**29**). The high resolution mass spectra (ESI^+^) HRMS spectra of all gold(I) complexes exhibit the classical peak *m/z* for the cationic fragment [M–X^−^]^+^.

### 2.2. Antiplasmodial Activity and Selectivity

The proligands (**Ln-R**) and the complexes (**Aubis(n-R)** and **Au(n-R)Cl**) as well as reference molecules, artemisinin, artemether, auranofin and the proligand precursor **DHA-C3**, were screened in vitro against the *P. falciparum* strain F32-Tanzania and the cytotoxicity on Vero cells was evaluated to determine the selectivity of the most active compounds (Table 1). Globally, the results are extremely interesting, with IC_50_ values against *Plasmodium* ranging from 9 to 935 nM for the proligands and the gold(I) complexes. The DHA-ether derivative used for the synthesis of the proligands containing a C3 lateral chain, **DHA-C3**, has an IC_50_ value of 8.5 nM comparable to that of the antimalarial reference drug artemether (IC_50_ = 6.1 nM). Surprisingly, for the imidazolium salts obtained by addition of a substituted imidazole moiety to **DHAC3-C5** precursors, a significant loss of the antimalarial activity was observed (IC_50_ = 98–935 nM, entries 5–9, 18-21 and 26–29). In contrast, the presence of the gold(I) cation in the corresponding complexes greatly improved the antiplasmodial efficacy (IC_50_ = 9–104 nM, entries 10–17, 22–25 and 30–33) compared to the corresponding proligands. The antiplasmodial activity was notable, with IC_50_ values below 100 nM for two proligands in the C5 series (**L5-Bn** (**11**) and **L5-Mes** (**12**) and for the majority of the cationic and neutral gold(I) complexes. By comparison, auranofin, a gold-based reference molecule used for the treatment of rheumatoid arthritis, was not active against *P. falciparum* parasites with a higher IC_50_ value of 1.5 µM.

Moreover, ten complexes are highly efficient, with IC_50_ values lower than 50 nM, including nine cationic gold(I) bis(NHC) complexes and one neutral NHC-Art complex, namely **Au(3-iPr)Cl)** (**27**). Among them, five complexes, namely **Aubis(3-iPr)** (**15**), **Aubis(3-Bn)** (**16**), **Aubis(3-Quin)** (**18**), **Aubis(4-Me)** (**19**) and **Aubis(5-Me)** (**23**) have an antiplasmodial activity comparable (an IC_50_ between 9 and 23 nM) to the reference antimalarial drugs artemisinin (IC_50_ = 18 nM) and artemether (IC_50_ = 6.1 nM). Regarding structure activity relationship, the potency of the complexes containing methyl (**14**, **19** and **23**) or benzyl (**16**, **20** and **24**) groups on the NHCs increased with the length of the spacer whereas, no correlation was highlighted for the mesityl and the quinoline series (**17**, **21**, **25**, and **18**, **22**, **26**, respectively). Surprisingly while artemether and **DHA-C3** had the same antiplasmodial activity, the selectivity indexes (SI) were largely higher for artemether (SI = 35,000) than for **DHA-C3** (SI = 294) demonstrating that the C3 moiety seems less selective. The selectivity indexes for the tested proligands and gold(I) complexes were between 8 and 255, the best value being obtained for the proligand **L5-Mes** (**12**, IC_50_ = 98 nM). Interestingly, regardless of the number of carbons in the spacer, hybrid molecules with aliphatic R groups (Me, iPr) have the best selectivities with SI values of 143 and 178 for **Aubis(3-Me)** (**14**) and **Au(3-iPr)Cl** (**27**), respectively, 62 for **Aubis(4-Me)** (**19**) and 111 for **Aubis(5-Me)** (**23**), in comparison with aromatic groups.

### 2.3. In Vitro Cross-Resistance between Hybrid Molecules and Artemisinin

Three hybrid molecules with the best selectivity indexes in each series, i.e., **Aubis(3-Me)** (**14**), **Aubis(4-Me)** (**19**) and **Aubis(5-Me)** (**23**), were evaluated in vitro for their efficacy in a context of resistance to artemisinins. For that, the comparison of the recovery capacity between the strain F32-ART, artemisinin-resistant, and its twin artemisinin-sensitive F32-TEM was evaluated after 48 h-treatment with the molecule to be tested via the recrudescence assay [9,15]. When ring-stage resistant parasites are exposed to artemisinin, most of them die, but a small sub-set of parasites is able to escape the treatment by a cell cycle arrest, called quiescence mechanism. This phenomenon is characterized by a halt of DNA synthesis, which explains the very low IC_50_ values obtained with all artemisinins even for the artemisinin-resistant strain F32-ART [9,15,30]. Therefore, the standard in vitro chemosensitivity assay, based on the measurement of DNA levels to estimate the inhibition of the parasite proliferation, is irrelevant to study the resistance to artemisinins and evaluate possible cross-resistances.

The validation of the test was here done thanks to the results obtained after 18 µM artemisinin treatment and demonstrating that F32-ART is able to recrudesce faster than F32-TEM with a difference to reach the initial parasitemia between the two strains of 9.7 days (Table 2). For the three hybrid molecules tested, namely **Aubis(3-Me)** (**14**), **Aubis(4-Me)** (**19**) and **Aubis(5-Me)** (**23**), Table 2 showed a difference of recrudescence between F32-ART and F32-TEM. Whatever the hybrid tested, the differences of recrudescence between both strains rise at the higher doses confirming that increase the doses of the molecules to be tested allows a better discrimination of the recrudescence capacity between the artemisinin-resistant and the -sensitive strains [9]. According to the obtained results, a cross-resistance between artemisinin and the hybrid molecules **Aubis(3-Me)** (**14**), **Aubis(4-Me)** (**19**) and **Aubis(5-Me)** (**23**) was noted. This cross-resistance could be explained by the DHA part of the hybrid, responsible for the quiescence entrance of the parasites and the lack of activity of the NHC part at the mitochondrial level due to limited access or pharmacodynamic properties. These data are in accordance with previously obtained results which highlighted the risks of parasites cross-resistance between artemisinins and endoperoxide-based compounds [31,32].

## 3. Materials and Methods

### 3.1. Chemistry

#### 3.1.1. General Information

All complexation reactions were performed under an inert atmosphere of dry nitrogen by using standard vacuum line and Schlenk tube techniques. Reactions involving silver compounds were performed with the exclusion of light. CH_3_CN was dried over CaH_2_ and subsequently distilled. 10β-(20-Bromopropoxy)dihydroartemisinin (**DHA-C3**) [27], 10β-(21-bromobutoxy)dihydroartemisinin **(DHA-C4)**, 10β-(22-bromopentoxy)dihydroartemisinin **(DHA-C5)**, 3′-methyl-1′-[10β-(20-propoxy)-dihydroartemisinin]1*H*-imidazol-3-ium bromide (**L3-Me** (**1**)), 3′-methyl-1′-[10β-(21-butoxy)-dihydroartemisinin]1*H*-imidazol-3-ium bromide (**L4-Me** (**2**)), 3′-methyl-1′-[10β-(22-pentoxy)-dihydroartemisinin]1*H*-imidazol-3-ium bromide (**L5-Me (3)**), complexes **Aubis(3-Me)** (**14**), **Aubis(4-Me)** (**19**), **Aubis(5-Me)** (**23**) [28], 1-mesitylimidazole [29] and 2-(2*H*-imidazol-1-yl)quinoline [22] were synthetized according to the referenced literature procedures. All other reagents were used as received from commercial suppliers. ^1^H- (300, 400 or 500 MHz) and ^13^C-NMR spectra (75, 101 or 126 MHz) were recorded at 298 K on AV300, AV400 or Avance 500 spectrometers (Bruker, Billerica, MA) in CDCl_3_ as solvent. All chemical shifts for ^1^H and ^13^C are relative to TMS using ^1^H (residual) or ^13^C chemical shifts of the solvent as a secondary standard. High Resolution Mass Spectrometry (HRMS) analysis were performed with a Xévo G2 QTOF spectrometer (Waters Corporation, Milford, MA) using electrospray ionization (ESI) by the “Service de Spectrométrie de Masse de Chimie UPS-CNRS (University of Toulouse, France)”. Elemental analyses were carried out by the “Service de Microanalyse du Laboratoire de Chimie de Coordination (Toulouse, France)”.

#### 3.1.2. Synthesis of proligands **(Ln-R)** and gold(I) complexes **Aubis(n-R)** and **Au(n-R)Cl**

*3′-Isopropyl-1′-[10β-(20-propoxy)dihydroartemisinin]1H-imidazol-3-ium bromide* (**L3-*i*Pr,** (**1**)). To a stirred solution of **DHA-C3** (164 mg, 0.4 mmol) in CH_3_CN (10 mL), 1-isopropylimidazole (61.7 mg, 0.4 mmol) was added and the reaction mixture was stirred 1 day at 70 °C. Then, the solvent was evaporated under reduced pressure and the viscous residue was washed with diethylether to afford a yellow powder (97 mg, 47% yield). Anal. Calcd. for C_24_H_39_BrN_2_O_5_: C, 55.92; H, 7.63; N, 5.43. Found C, 55.85; H, 7.64; N, 5.89. ^1^H-NMR (300 MHz, CDCl_3_): δ 10.67 (s, 1H, H_2_), 7.48 (d, *J* = 1.8 Hz, 1H, H_4_), 7.40 (d, *J* = 1.8 Hz, 1H, H_5_), 5.38 (s, 1H, H_Art_), 4.93 (h, *J* = 6.6, 5.6 Hz, 1H, H_iPr_), 4.78 (d, *J* = 3.2 Hz, 1H, H_Art_), 4.52–4.46 (m, 2H, H_CH2_), 3.92–3.84 (m, 1H, H_CH2_), 3.54–3.46 (m, 1H, H_CH2_), 2.71–2.57 (m, 1H, H_Art_), 2.40–2.22 (m, 3H, H_Art_, H_CH2_), 2.07–1.97 (s, 2H, H_Art_), 1.96–1.84 (m, 1H, H_Art_), 1.81–1.69 (m, 2H, H_Art_), 1.65 (s, 3H, H_iPr_), 1.63 (s, 3H, H_iPr_), 1.54–1.45 (m, 2H, H_Art_), 1.41 (s, 3H, H_Art_), 1.38–1.32 (m, 1H, H_Art_), 1.29–1.20 (m, 1H, H_Art_), 0.96 (d, *J* = 6.1 Hz, 3H, H_Art_), 0.95–0.86 (m, 1H, H_Art_), 0.92 (d, *J* = 7.4 Hz, 3H, H_Art_). ^13^C NMR (101 MHz, CDCl_3_): δ 136.5 (1C, C_2_), 122.3 (1C, C_4_), 119.9 (1C, C_5_), 104.2 (1C, C_Art_), 102.2 (1C, C_Art_), 88.0 (1C, C_Art_), 80.9 (1C, C_Art_), 64.7 (1C, C_CH2_), 53.5 (1C, C_iPr_), 52.4 (1C, C_Art_), 47.4 (1C, C_CH2_), 44.2 (1C, C_Art_), 37.5 (1C, C_Art_), 36.3 (1C, C_Art_), 34.5 (1C, C_Art_), 30.8 (1C, C_CH2_), 30.7 (1C, C_Art_), 26.1 (1C, C_Art_), 24.6 (1C, C_Art_), 24.6 (1C, C_Art_), 23.2 (2C, C_iPr_), 20.3 (1C, C_Art_), 13.2 (1C, C_Art_). HRMS (ESI^+^): calcd. for C_24_H_39_N_2_O_5_ *m/z* = 435.2859, found 435.2856.

*3′-Benzyl-1′-[10β-(20-propoxy)dihydroartemisinin]1H-imidazol-3-ium bromide* (**L3-Bn**, (**2**)). To a stirred solution of **DHA-C3** (275 mg, 0.68 mmol) in CH_3_CN (4 mL) heated at 70 °C, 1-benzylimidazole (107 mg, 0.68 mmol) was added and the reaction mixture was stirred 3 days. Then, the solvent was removed under reduced pressure and the crude product was dissolved in CH_2_Cl_2_ and precipitated with Et_2_O. This treatment was repeated three times to afford a sticky white solid (286 mg, 87% yield). Anal. Calcd. for C_28_H_39_BrN_2_O_5_: C, 59.68; H, 6.98; N, 4.97. Found C, 59.57; H, 6.95; N, 4.90. ^1^H-NMR (400 MHz, CDCl_3_): δ 10.71 (s, 1H, H_2_), 7.52 (m, 1H, H_Bn_), 7.51 (d, *J* = 1.8 Hz, 1H, H_4_), 7.40 (d, *J* = 1.8 Hz, 1H, H_5_), 7.39 (m, 2H, H_Bn_), 7.34 (d, *J* = 1.6 Hz, 2H, H_Bn_), 5.64 (s, 2H, H_Bn_), 5.37 (s, 1H, H_Art_), 4.76 (d, *J* = 3.6 Hz, 1H, H_Art_), 4.40-4.44 (m, 2H, H_CH2_), 3.90–3.85 (m, 1H, H_CH2_), 3.51–3.44 (m, 1H, H_CH2_), 2.62–2.66 (m, 1H, H_Art_), 2.40–2.32 (m, 1H, H_Art_), 2.29–2.23 (m, 2H, H_CH2_), 2.05 (m, 1H, H_Art_), 2.01 (m, 1H, H_Art_),1.92–1.86 (m, 1H, H_Art_), 1.78–1.74 (m, 1H, H_Art_), 1.69–1.63 (m, 2H, H_Art_), 1.50–1.43 (m, 2H, H_Art_), 1.41 (s, 3H, H_Art_), 1.33–1.37 (m, 1H, H_Art_), 1.28–1.24 (m, 1H, H_Art_), 0.96 (d, *J* = 6.3 Hz, 3H, H_Art_), 0.92 (m, 1H, H_Art_), 0.89 (d, *J* = 7.4 Hz, 3H, H_Art_). ^13^C NMR (101 MHz, CDCl_3_): δ 137.7 (1C, C_2_), 132.7 (1C, C_Bn_), 129.7 (2C, C_Bn_), 129.5 (1C, C_Bn_), 129.1 (2C, C_Bn_), 122.1 (1C, C_4_), 121.5 (1C, C_5_), 104.3 (1C, C_Art_), 102.3 (1C, C_Art_), 88.0 (1C, C_Art_), 80.9 (1C, C_Art_), 64.6 (1C, C_CH2_), 53.6 (1C, C_Bn_), 52.4 (1C, C_Art_), 47.6 (1C, C_CH2_), 44.2 (1C, C_Art_), 37.5 (1C, C_Art_), 36.3 (1C, C_Art_), 34.4 (1C, C_Art_), 30.8 (1C, C_CH2_), 30.5 (1C, C_Art_), 26.1 (1C, C_Art_), 24.7 (1C, C_Art_), 24.6 (1C, C_Art_), 20.3 (1C, C_Art_), 13.1 (1C, C_Art_). HRMS (ESI^+^): calcd. for C_28_H_39_N_2_O_5_ *m/z* = 483.2855, found 483.2859.

*3′-Mesityl-1′-[10β-(20-propoxy)dihydroartemisinin]1H-imidazol-3-ium bromide* (**L3-Mes**, (**3**)). To a stirred solution of **DHA-C3** (251 mg, 0.62 mmol) in CH_3_CN (4 mL) heated at 70 °C, 1-mesitylimidazole (115 mg, 0.62 mmol) was added and the reaction mixture was stirred for 3 days. The solvent was removed under reduced pressure and the crude product was dissolved in CH_2_Cl_2_ and precipitated with Et_2_O. This treatment was repeated three times to afford a white solid (238 mg, 76% yield). Anal. Calcd. for C_30_H_43_BrN_2_O_5_: C, 60.91; H, 7.33; N, 4.74. Found C, 60.93; H, 7.27; N, 4.65. ^1^H-NMR (300 MHz, CDCl_3_): δ 10.49 (s, 1H, H_2_), 7.76 (t, *J* = 1.8 Hz, 1H, H_4_), 7.17 (t, *J* = 1.8 Hz, 1H, H_4_), 7.03 (m, 2H, H_Mes_), 5.45 (s, 1H, H_Art_), 4.92 (m, 1H, H_Art_), 4.87–4.76 (m, 2H, H_CH2_), 3.90–3.83 (m, 1H, H_CH2_), 3.65–3.54 (m, 1H, H_CH2_), 2.71–2.66 (m, 1H, H_Art_), 2.36 (s, 3H, H_Mes_), 2.44–2.33 (m, 3H, H_Art_), 2.11 (s, 6H, H_Mes_), 2.06–2.00 (m, 1H, H_Art_), 1.95–1.84 (m, 1H, H_Art_), 1.83–1.78 (m, 1H, H_Art_), 1.77–1.67 (m, 2H, H_Art_), 1.65 (s, 1H, H_Art_), 1.54–1.48 (m, 2H, H_Art_), 1.45–1.36 (m, 1H, H_Art_), 1.37 (s, 3H, H_Art_), 1.32–1.26 (m, 1H, H_Art_), 0.98 (d, *J* = 6.2 Hz, 3H, H_Art_), 0.94 (m, 1H, H_Art_), 0.93 (d, *J* = 7.4 Hz, 3H, H_Art_). ^13^C NMR (101 MHz, CDCl_3_): δ 141.2 (1C, C_Mes_), 137.7 (1C, C_2_), 134.2 (2C, C_Mes_), 130.7 (1C, C_Mes_), 129.8 (2C, C_Mes_), 123.6 (1C, C_4_), 123.6 (1C, C_5_), 104.2 (1C, C_Art_), 102.2 (1C, C_Art_), 87.9 (1C, C_Art_), 81.0 (1C, C_Art_), 64.7 (1C, C_CH2_), 52.4 (1C, C_Art_), 47.6 (1C, C_CH2_), 44.2 (1C, C_Art_), 37.4 (1C, C_Art_), 36.3 (1C, C_Art_), 36.1 (1C, C_Art_), 34.4 (1C, C_CH2_), 30.9 (1C, C_Art_), 30.1 (1C, C_Art_), 25.9 (1C, C_Art_), 24.6 (1C, C_Art_), 24.5 (1C, C_Art_), 21.1 (1C, C_Mes_), 20.3 (1C, C_Art_), 17.6 (1C, C_Mes_), 13.2 (1C, C_Art_). HRMS (ESI^+^): calcd. for C_30_H_43_N_2_O_5_ *m/z* = 511.3174, found 511.3172.

*3′-Quinolin-2-yl-1′-[10β-(20-propoxy)dihydroartemisinin]1H-imidazol-3-ium bromide* (**L3-Quin**, (**4**)). To a stirred solution of **DHA-C3** (292 mg, 0.72 mmol) in CH_3_CN (3 mL) heated at 70)° C, 1-(quinolin-2-yl)-imidazole (140 mg, 0.72 mmol) was added and the reaction mixture was stirred for 3 days. Then, the solvent was removed under reduced pressure and the crude product was dissolved in CH_2_Cl_2_ and precipitated with Et_2_O. This treatment was repeated three times to afford a yellow solid (315 mg, 84 % yield). Anal. Calcd. for C_30_H_38_BrN_3_O_5_: C, 60.00; H, 6.38; N, 7.00. Found C, 60.15; H, 6.42; N, 6.95. ^1^H-NMR (400 MHz, CDCl_3_): δ 12.15 (s, 1H, H_2_), 8.71 (m, 1H, H_Quin_), 8.56–8.53 (m, 2H, H_4_, H_Quin_), 8.06 (d, *J* = 8.5 Hz, 1H, H_Quin_), 7.96 (d, *J* = 7.8 Hz, 1H, H_Quin_), 7.84 (m, 1H, H_Quin_), 7.68 (m, 1H, H_Quin_), 7.54 (t, *J* = 1.7 Hz, 1H, H_5_), 5.43 (s, 1H, H_Art_), 4.83 (d, *J* = 3.6 Hz, 1H, H_Art_), 4.75 (m, 2H, H_CH2_), 4.78–4.70 (m, 2H, H_CH2_), 4.02–3.96 (m, 1H, H_CH2_), 3.65–3.60 (m, 1H, H_CH2_), 2.68 (m, 1H, H_Art_), 2.47–2.41 (m, 2H, H_CH2_), 2.39–2.35 (m, 1H, H_Art_), 2.08–2.02 (m, 1H, H_Art_), 1.94–1.84 (m, 1H, H_Art_), 1.82–1.77 (m, 1H, H_Art_), 1.72–1.65 (m, 2H, H_Art_), 1.56–1.45 (m, 2H, H_Art_), 1.44 (s, 3H, H_Art_), 1.40–1.34 (m, 1H, H_Art_), 1.30–1.23 (m, 1H, H_Art_), 0.97 (d, *J* = 6.4 Hz, 3H, H_Art_), 0.95 (d, *J* = 7.4 Hz, 3H, H_Art_), 0.91–0.85 (m, 1H, H_Art_). ^13^C NMR (101 MHz, CDCl_3_): δ 146.1 (1C, C_Quin_), 144.5 (1C, C_Quin_), 141.7 (1C, C_Quin_), 137.0 (1C, C_2_), 131.5 (1C, C_Quin_), 128.8 (1C, C_Quin_), 128.3 (1C, C_Quin_), 128.2 (2C, C_Quin_), 122.4 (1C, C_4_), 118.7 (1C, C_5_), 112.7 (1C, C_Quin_), 104.3 (1C, C_Art_), 102.3 (1C, C_Art_), 88.0 (1C, C_Art_), 80.9 (1C, C_Art_), 64.8 (1C, C_CH2_), 52.4 (1C, C_Art_), 49.2 (1C, C_CH2_), 44.2 (1C, C_Art_), 37.5 (1C, C_Art_), 36.3 (1C, C_Art_), 34.5 (1C, C_Art_), 30.8 (1C, C_CH2_), 30.8 (1C, C_Art_), 26.1 (1C, C_Art_), 24.6 (1C, C_Art_), 24.6 (1C, C_Art_), 20.3 (1C, C_Art_), 13.2 (1C, C_Art_). HRMS (ESI^+^): calcd. for C_30_H_38_N_3_O_5_ *m/z* = 520.2798, found 520.2811.

*Complex***Aubis(3-iPr)** (**15**). Under a nitrogen atmosphere and protection of the light, **L3-*i*Pr** (102 mg, 0.2 mmol) and Ag_2_O (26 mg, 0.11 mmol) was dissolved in CH_3_CN (3 mL) and stirred overnight at rt. Then, AgNO_3_ (19 mg, 0.11 mmol) was added to the mixture followed 2 h later by addition of **Au(SMe_2_)Cl** (32 mg, 0.11 mmol). After stirring 1 h at rt, the solution was filtered through a pad of celite and the solvent removed under reduced pressure to afford a white solid after centrifugation (95 mg, 83% yield). Anal. Calcd. For C_48_H_76_AuClN_4_O_10_: C, 52.34; H, 6.95; N, 5.09. Found C, 52.28; H, 6.85; N, 5.02. ^1^H-NMR (400 MHz, CDCl_3_): δ 7.31 (d, *J* = 1.9 Hz, 1H, H_4_), 7.30 (s, 1H, H_5_), 5.39 (s, 1H, H_Art_), 4.95 (h, *J* = 6.9 Hz, 1H, H_iPr_), 4.80 (d, *J* = 3.6 Hz, 1H, H_Art_), 4.42–4.31 (m, 2H, H_CH2_), 3.95–3.89 (m, 1H, H_CH2_), 3.49–3.43 (m, 1H, H_CH2_), 2.68–2.64 (m, 1H, H_Art_), 2.39 (td, *J* = 14.0, 3.9 Hz, 1H, H_Art_), 2.31–2.18 (m, 2H, H_CH2_), 2.08–2.03 (m, 1H, H_Art_), 1.94–1.89 (m, 1H, H_Art_), 1.78–1.65 (m, 3H, H_Art_), 1.62 (s, 3H, H_iPr_), 1.60 (s, 3H, H_iPr_), 1.52–1.47 (m, 2H, H_Art_), 1.44 (s, 3H, H_Art_), 1.36–1.25 (m, 2H, H_Art_), 0.98 (d, *J* = 6.1 Hz, 3H, H_Art_), 0.97–0.88 (m, 1H, H_Art_), 0.93 (d, *J* = 7.3 Hz, 3H, Art). ^13^C NMR (101 MHz, CDCl_3_): δ 182.1 (1C, C_2_), 122.3 (1C, C_4_), 118.3 (1C, C_5_), 104.2 (1C, C_Art_), 102.0 (1C, C_Art_), 88.0 (1C, C_Art_), 80.9 (1C, C_Art_), 64.8 (1C, C_CH2_), 53.8 (1C, C_iPr_), 52.4 (1C, C_Art_), 48.9 (1C, C_CH2_), 44.2 (1C, C_Art_), 37.5 (1C, C_Art_), 36.4 (1C, C_Art_), 34.5 (1C, C_Art_), 31.7 (1C, C_CH2_), 30.8 (1C, C_Art_), 26.1 (1C, C_Art_), 24.7 (1C, C_Art_), 24.6 (1C, C_Art_), 23.8 (1C, C_iPr_), 23.8 (1C, C_iPr_), 20.3 (1C, C_Art_), 13.2 (1C, C_Art_). HRMS (ESI^+^): calcd. for C_48_H_76_AuClN_4_O_10_ *m/z* = 1065.5227, found 1065.5220.

*Complex***Aubis(3-Bn)** (**16**). Under a nitrogen atmosphere, K_2_CO_3_ (26 mg, 0.19 mmol) was added to **L3-Bn** (108 mg, 0.19 mmol) in dry CH_3_CN (4 mL) and heated at 60 °C under stirring. Then, **Au(SMe_2_)Cl** (28 mg, 0.096 mmol) was added and the mixture was stirred for 10 h. After cooling to room temperature, the solution was filtered through a pad of celite and the solvent removed under reduced pressure. The complex was purified by flash chromatography on silica with CH_2_Cl_2_-MeOH as eluent (100/0 to 100/10) to give a white solid (75 mg, 65 % yield). Anal. Calcd. for C_56_H_76_AuClN_4_O_10_: C, 56.16; H, 6.40; N, 4.48. Found C, 56.28; H, 6.55; N, 4.42. ^1^H-NMR (500 MHz, CDCl_3_): δ 7.34 (d, *J* = 1.9 Hz, 1H, H_4_), 7.30 (m, 2H, H_Bn_), 7.29 (m, 1H, H_Bn_), 7.24 (m, 2H, H_Bn_), 7.23 (d, *J* = 1.9 Hz, 1H, H_5_), 5.39 (s, 2H, H_Bn_), 4.74 (d, *J* = 3.5 Hz, 1H, H_Art_), 4.28–4.24 (m, 2H, H_CH2_), 3.87–3.81 (m, 1H, H_CH2_), 3.41–3.36 (m, 1H, H_CH2_), 2.64–2.60 (m, 1H, H_Art_), 2.41–2.33 (m, 1H, H_Art_), 2.11 (m, 2H, H_CH2_), 2.07–2.01 (m, 1H, H_Art_), 1.91–1.85 (m, 2H, H_Art_), 1.74–1.69 (m, 2H, H_Art_), 1.63–1.57 (m, 1H, H_Art_), 1.41–1.46 (m, 2H, H_Art_), 1.41 (s, 3H, H_Art_), 1.33–1.26 (m, 2H, H_Art_), 0.94 (d, *J* = 6.1 Hz, 3H, H_Art_), 0.92 (m, 1H, H_Art_), 0.87 (d, *J* = 7.3 Hz, 3H, H_Art_). ^13^C NMR (126 MHz, CDCl_3_): δ 183.5 (1C, C_2_), 135.7 (1C, C_Bn_), 129.1 (2C, C_Bn_), 128.6 (1C, C_Bn_), 127.6 (2C, C_Bn_), 122.5 (1C, C_4_), 122.2 (1C, C_5_), 104.2 (1C, C_Art_), 102.0 (1C, C_Art_), 87.9 (1C, C_Art_), 80.9 (1C, C_Art_), 64.7 (1C, C_CH2_), 54.8 (1C, C_Bn_), 52.5 (1C, C_Art_), 48.6 (1C, C_CH2_), 44.2 (1C, C_Art_), 37.6 (1C, C_Art_), 36.4 (1C, C_Art_), 34.5 (1C, C_Art_), 31.6 (1C, C_CH2_), 30.7 (1C, C_Art_), 26.2 (1C, C_Art_), 24.7 (1C, C_Art_), 24.5 (1C, C_Art_), 20.3 (1C, C_Art_), 13.1 (1C, C_Art_). HRMS (ESI^+^): calcd. for C_56_H_76_AuN_4_O_10_ *m/z* = 1161.5227, found 1161.5247.

*Complex***Aubis(3-Mes)** (**17**). Under a nitrogen atmosphere, K_2_CO_3_ (24 mg, 0.17 mmol) was added to L**3-Mes** (104 mg, 0.17 mmol) in dry CH_3_CN (4 mL). The mixture was then heated at 60 °C. After, **Au(SMe_2_)Cl** (26 mg, 0.087 mmol) was added and the mixture was stirred for 10 h. After cooling to room temperature, the solution was filtered through a pad of celite and the solvent removed under reduced pressure. The complex was purified by flash chromatography on silica with CH_2_Cl_2_-MeOH as eluent (100/0 to 100/10) to give a yellow solid (58 mg, 52% yield). Anal. Calcd. for C_60_H_84_AuClN_4_O_10_: C, 57.48; H, 6.75; N, 4.47. Found C, 57.42; H, 6.68; N, 4.35. ^1^H-NMR (500 MHz, CDCl_3_): δ 7.59 (d, *J* = 1.9 Hz,1H, H_4_), 6.96 (m, 3H, H_5_, H_Mes_), 5.37 (s, 1H, H_Art_), 4.73 (d, *J* = 3.5 Hz, 1H, H_1Art_), 4.13 (m, 2H, H_CH2_), 3.72 (m, 1H, H_CH2_), 3.22 (m, 1H, H_CH2_), 2.65 (m, 1H, H_Art_), 2.37 (s, 3H, H_Mes_), 2.41–2.33 (m, 1H, H_Art_), 2.09–1.98 (m, 2H, H_CH2_), 1.90 (m, 1H, H_Art_), 1.85 (s, 3H, H_Mes_), 1.83 (m, 3H, H_Mes_), 1.83–1.65 (m, 4H, H_Art_), 1.51–1.45 (m, 2H, H_Art_), 1.43 (m, 1H, H_Art_), 1.36–1.32 (m, 1H, H_Art_), 1.29–1.25 (s, 1H, H_Art_), 0.98 (d, *J* = 6.3 Hz, 3H, H_Art_), 0.96–0.88 (m, 1H, H_Art_), 0.94 (d, *J* = 7.2 Hz, 3H, H_Art_). ^13^C NMR (126 MHz, CDCl_3_): δ 183.8 (1C, C_2_), 139.6 (1C, C_Mes_), 134.8 (2C, C_Mes_), 134.7 (1C, C_Mes_), 129.2 (2C, C_Mes_), 123.0 (1C, C_5_), 122.7 (1C, C_4_), 104.1 (1C, C_Art_), 102.0 (1C, C_Art_), 87.9 (1C, C_Art_), 81.0 (1C, C_Art_), 64.8 (1C, C_CH2_), 52.5 (1C, C_Art_), 48.3 (1C, C_CH2_), 44.2 (1C, C_Art_), 37.7 (1C, C_Art_), 36.4 (1C, C_Art_), 34.6 (1C, C_Art_), 31.6 (C_CH2_), 30.9 (1C, C_Art_), 26.1 (1C, C_Art_), 24.7 (1C, C_Art_), 24.6 (1C, C_Art_), 21.2 (2C, C_Mes_), 20.3 (1C, C_Art_), 17.6 (1C, C_Mes_), 13.3 (1C, C_Art_). HRMS (ESI^+^): calcd. for C_60_H_74_AuN_6_O_10_ *m/z* = 1235.5157, found 1235.5132.

*Complex***Aubis(3-Quin)** (**18**). Under a nitrogen atmosphere, K_2_CO_3_ (24 mg, 0.17 mmol) was added to L**3-Quin** (100 mg, 0.17 mmol) in dry CH_3_CN (4 mL). The stirred mixture was then heated at 60 °C. Then, **Au(SMe_2_)Cl** (25 mg, 0.085 mmol) was added and the mixture was stirred for 10 h. After cooling to room temperature, the solution was filtered through a pad of celite and the solvent removed under reduced pressure to give a white solid (93 mg, 86% yield). Anal. Calcd. For C_60_H_74_AuClN_6_O_10_: C, 56.67; H, 5.87; N, 6.61. Found C, 56.62; H, 5.85; N, 6.54. ^1^H-NMR (500 MHz, CDCl_3_): δ 8.30 (d, *J* = 8.6 Hz, 1H, H_Quin_), 8.13 (d, *J* = 8.7 Hz, 1H, H_Quin_), 8.09 (d, J = 1.6 Hz, 1H, H_4_), 7.80 (d, *J* = 8.4 Hz, 1H, H_Quin_), 7.73 (d, *J* = 8.2 Hz, 1H, H_Quin_), 7.62 (m, 1H, H_Quin_), 7.51 (d, *J* = 2.0 Hz, 1H, H_5_), 7.47 (m, 1H, H_Quin_), 5.34 (s, 1H, H_Art_), 4.71 (d, *J* = 3.5 Hz, 1H, H_Art_), 4.53–4.44 (m, 2H, H_CH2_), 3.87–3.82 (m, 1H, H_CH2_), 3.43–3.38 (m, 1H, H_Art_), 2.61–2.56 (m, 1H, H_Art_), 2.33 (td, *J* = 14.0, 4.0 Hz, 1H, H_Art_), 2.26–2.18 (m, 2H, H_CH2_), 2.02–1.97 (m, 1H, H_Art_), 1.86–1.81 (m, 1H, H_Art_), 1.67–1.62 (m, 2H, H_Art_), 1.54–1.50 (m, 1H, H_Art_), 1.46–1.35 (m, 2H, H_Art_), 1.39 (m, 3H, H_Art_), 1.30-1.16 (m, 2H, H_Art_), 0.88 (d, *J* = 6.2 Hz, 3H, H_Art_), 0.86–080 (m, 1H, H_Art_), 0.83 (d, *J* = 7.2 Hz, 3H, H_Art_). ^13^C NMR (126 MHz, CDCl_3_): δ 181.8 (1C, C_2_), 148.9 (1C, C_Quin_), 146.3 (1C, C_Quin_), 139.9 (1C, C_Quin_), 131.0 (1C, C_Quin_), 128.4 (1C, C_Quin_), 127.9 (1C, C_Quin_), 127.6 (1C, C_Quin_), 127.5 (1C, C_Quin_), 122.9 (1C, C_4_), 121.6 (1C, C_5_), 115.7 (1C, C_Quin_), 104.2 (1C, C_Art_), 102.0 (1C, C_Art_), 87.9 (1C, C_Art_), 80.9 (1C, C_Art_), 64.8 (1C, C_CH2_), 52.4 (C1, C_Art_), 49.8 (1C, C_CH2_), 44.2 (1C, C_Art_), 37.6 (1C, C_Art_), 36.3 (1C, C_Art_), 34.5 (1C, C_Art_), 31.5 (1C, C_CH2_), 30.7 (1C, C_Art_), 26.1 (1C, C_Art_), 24.6 (1C, C_Art_), 24.5 (1C, C_Art_), 20.3 (1C, C_Art_), 13.1 (1C, C_Art_). HRMS (ESI^+^): calcd. for C_60_H_74_AuN_6_O_10_ *m/z* = 1235.5157, found 1235.5132.

*Complex***Au(3-*i*Pr)Cl** (**27**). Under a nitrogen atmosphere and protection of the light, L**3-*i*Pr** (21 mg, 0.04 mmol), K_2_CO_3_ (6 mg, 0.04 mmol) and **Au(SMe_2_)Cl** (12 mg, 0.04 mmol) were dissolved in CH_3_CN (3 mL) and stirred for 6 h at 60 °C. The solution was filtered through a syringe filter (0.2 µm) and the solvent removed under reduced pressure to afford a white powder (9.8 mg, 42% yield). Anal. Calcd. for C_24_H_38_AuClN_2_O_5_: C, 43.22; H, 5.74; N, 4.20. Found C, 43.32; H, 5.72; N, 4.15. ^1^H-NMR (400 MHz, CDCl_3_): δ 7.01 (d, *J* = 2.0 Hz, 1H, H_4_), 6.97 (d, *J* = 1.9 Hz, 1H, H_5_), 5.41 (s, 1H, H_Art_), 5.10 (hept, *J* = 7.0 Hz, 1H, H_iPr_), 4.81 (d, *J* = 3.4 Hz, 1H, H_Art_), 4.26 (td, *J* = 7.1, 4.8 Hz, 2H, H_CH2_), 3.95–3.86 (m, 1H, H_CH2_), 3.48–3.40 (m, 1H, H_CH2_), 2.73–2.63 (m, 1H, H_Art_), 2.45–2.33 (m, 1H, H_Art_), 2.23–2.13 (m, 2H, H_CH2_), 2.06 (ddd, *J* = 14.0, 4.6, 2.9 Hz, 1H, H_Art_), 1.95–1.83 (m, 2H, H_Art_), 1.78–1.70 (m, 2H, H_Art_), 1.60–1.52 (s, 2H, H_Art_), 1.50 (s, 3H, H_iPr_), 1.48 (s, 3H, H_iPr_), 1.45 (s, 3H, H_Art_), 1.41–1.36 (m, 1H, H_Art_), 1.30–1.24 (m, 1H, H_Art_), 0.99 (d, *J* = 5.5 Hz, 3H, H_Art_), 0.98 (d, *J* = 5.5 Hz, 3H, H_Art_), 0.96–0.86 (m, 1H, H_Art_). ^13^C NMR (101 MHz, CDCl_3_): δ 173.3 (1C, C_2_), 120.9 (1C, C_4_), 116.4 (1C, C_5_), 104.2 (1C, C_Art_), 102.2 (1C, C_Art_), 88.0 (1C, C_Art_), 81.0 (1C, C_Art_), 64.8 (1C, C_CH2_), 53.6 (1C, C_iPr_), 52.5 (1C, C_Art_), 48.7 (1C, C_CH2_), 44.3 (1C, C_Art_), 37.5 (1C, C_Art_), 36.4 (1C, C_Art_), 34.5 (1C, C_Art_), 31.3 (1C, C_CH2_), 30.9 (1C, C_Art_), 29.7 (1C, C_Art_), 26.2 (1C, C_Art_), 24.7 (2C, C_Art_), 23.4 (2C, C_iPr_), 20.3 (1C, C_Art_), 13.3 (1C, C_Art_). HRMS (ESI^+^): calcd. for C_24_H_38_AuN_2_O_5_ *m/z* = 631.2449, found 631.2446.

*Complex***Au(3-Bn)Cl** (**28**). Under a nitrogen atmosphere, K_2_CO_3_ (20 mg, 0.15 mmol) was added to L**3-Bn** (85 mg, 0.15 mmol) in dry CH_3_CN (7 mL). The mixture was then heated at 60 °C. After, **Au(SMe_2_)Cl** (44 mg, 0.15 mmol) was added and the mixture was stirred for 10 h. After cooling to room temperature, the solution was filtered through a pad of celite and the solvent was removed under reduced pressure to give a white solid (87 mg, 81% yield). Anal. Calcd. for C_28_H_38_AuClN_2_O_5_: C, 47.03; H, 5.36; N, 3.92. Found C, 47.12; H, 5.29; N, 3.91. ^1^H-NMR (400 MHz, CDCl_3_): δ 7.31–7.40 (m, 5H, H_Bn_), 6.97 (d, *J* = 2.0 Hz, 1H, H_4_), 6.89 (d, *J* = 1.9 Hz, 1H, H_5_), 5.38 (s, 2H, H_Bn_), 5.40 (s, 1H, H_Art_), 4.79 (d, *J* = 3.5 Hz, 1H, H_Art_), 4.31–4.24 (m, 2H, H_CH2_), 3.92–3.87 (m, 1H, H_CH2_), 3.46–4.40 (m, 1H, H_CH2_), 2.68–2.63 (m, 1H, H_Art_), 2.41–2.32 (ddd, *J* = 14.5, 13.4, 3.9 Hz, 1H, H_Art_), 2.24–2.21 (m, 2H, H_CH2_), 2.04 (ddd, *J* = 14.6, 4.8, 2.9 Hz, 1H, H_Art_), 1.93–1.68 (m, 4H, H_Art_), 1.55–1.44 (m, 2H, H_Art_), 1.43 (s, 3H, H_Art_), 1.39–1.33 (m, 1H, H_Art_), 1.29–1.22 (m, 1H, H_Art_), 0.97 (d, *J* = 6.1 Hz, 3H, H_Art_), 0.95 (d, *J* = 7.3 Hz, 3H, H_Art_), 0.94–0.88 (m, 1H, H_Art_). ^13^C NMR (101 MHz, CDCl_3_): δ 174.5 (1C, C_2_), 134.9 (1C, C_Bn_), 129.1 (2C, C_Bn_), 128.8 (2C, C_Bn_), 128.1 (2C, C_Bn_), 121.2 (1C, C_4_), 120.2 (1C, C_5_), 104.2 (1C, C_Art_), 102.2 (1C, C_Art_), 88.0 (1C, C_Art_), 81.0 (1C, C_Art_), 64.7 (1C, C_CH2_), 55.2 (1C, C_Bn_), 52.5 (1C, C_Art_), 48.7 (1C, C_CH2_), 44.3 (1C, C_Art_), 37.5 (1C, C_Art_), 36.4 (1C, C_Art_), 34.5 (1C, C_Art_), 31.2 (1C, C_CH2_), 30.9 (1C, C_Art_), 26.2 (1C, C_1Art_), 24.7 (2C, C_Art_), 20.3 (1C, C_Art_), 13.3 (C_Art_). HRMS (ESI^+^): calcd. for C_28_H_38_AuN_2_O_5_ *m/z* = 679.2452, found 679.2446.

*Complex***Au(3-Quin)Cl** (**29**) was obtained as a byproduct from the purification by column chromatography of **Aubis(3-Quin)** (8 mg). Anal. Calcd. For C_30_H_37_AuClN_3_O_5_: C, 47.91; H, 4.96; N, 5.59. Found C, 47.85; H, 5.08; N, 5.49. ^1^H-NMR (400 MHz, CDCl_3_): δ 8.78 (d, *J* = 8.7 Hz, 1H, H_Quin_), 8.41 (d, *J* = 8.6 Hz, 1H, H_Quin_), 8.07 (d, *J* = 8.2 Hz, 1H, H_Quin_), 8.04 (d, *J* = 2.0 Hz, 1H, H_4_), 7.93 (dd, *J* = 8.2, 1.4 Hz, 1H, H_Quin_), 7.81 (ddd, *J* = 8.5, 6.9, 1.5 Hz, 1H, H_Quin_), 7.64 (ddd, *J* = 8.1, 6.9, 1.2 Hz, 1H, H_Quin_), 7.20 (d, *J* = 2.1 Hz, 1H, H_5_), 5.44 (s, 1H, H_Art_), 4.84 (d, *J* = 3.6 Hz, 1H, H_Art_), 4.48–4.44 (m, 2H, H_CH2_), 4.01–3.96 (m, 1H, H_CH2_), 3.56–3.50 (m, 1H, H_CH2_), 2.69 (m, 1H, H_Art_), 2.44–2.36 (m, 1H, H_Art_), 2.31–2.27 (m, 2H, H_CH2_), 2.09–2.03 (m, 1H, H_Art_), 1.93–1.85 (m, 2H, H_Art_), 1.79–1.71 (m, 2H, H_Art_), 1.55–1.45 (m, 2H, H_Art_), 1.46 (s, 3H, H_Art_), 1.41–1.26 (m, 2H, H_Art_), 0.99 (d, *J* = 6.3 Hz, 3H, H_Art_), 0.97 (d, *J* = 6.3 Hz, 3H, H_Art_), 0.94–0.90 (m, 1H, H_Art_). ^13^C NMR (101 MHz, CDCl_3_): δ 173.7 (1C, C_2_), 149.1 (1C, C_Quin_), 146.5 (1C, C_Quin_), 139.6 (1C, C_Quin_), 130.9 (1C, C_Quin_), 128.9 (1C, C_Quin_), 127.9 (1C, C_Quin_), 127.9 (1C, C_Quin_), 127.5 (1C, C_Quin_), 121.2 (1C, C_5_), 120.6 (1C, C_4_), 115.4 (C_Quin_), 104.2 (1C, C_Art_), 102.2 (1C, C_Art_), 88.0 (1C, C_Art_), 81.0 (1C, C_Art_), 64.8 (1C, C_CH2_), 52.5 (1C, C_Art_), 49.7 (1C, C_CH2_), 44.3 (1C, C_Art_), 37.5 (1C, C_Art_), 36.4 (1C, C_Art_), 34.5 (1C, C_Art_), 31.3 (1C, C_CH2_), 30.9 (1C, C_Art_), 26.2 (1C, C_Art_), 24.7 (2C, C_Art_), 20.3 (1C, C_Art_), 13.3 (1C, C_Art_). HRMS (ESI^+^): calcd. for C_30_H_37_AuN_3_O_5_ *m/z* = 716.2399, found 716.2418.

*3′-Benzyl-1′-[10β-(21-butoxy)dihydroartemisinin]1H-imidazol-3-ium bromide* (**L4-Bn** (**7**)). To a stirred solution of **DHA-C4** (109 mg, 0.26 mmol) in CH_3_CN (4 mL) heated at 70 °C, 1-benzylimidazole (41 mg, 0.26 mmol) was added and the reaction mixture was stirred 3 days. Then, the solvent was removed under reduced pressure and the crude product was dissolved in CH_2_Cl_2_ and precipitated with Et_2_O. This treatment was repeated three times to afford a white solid (109 mg, 73% yield). Anal. Calcd. for C_29_H_41_BrN_2_O_5_: C, 60.31; H, 7.16; N, 4.85. Found C, 60.26; H, 7.07; N, 4.79. ^1^H-NMR (300 MHz, CDCl_3_): δ 11.19 (sl, 1H, H_2_), 7.50–7.43 (m, 5H, H_Bn_), 7.23–7.02 (m, 2H, H_4_, H_5_), 5.62 (s, 2H, H_Bn_), 5.38 (s, 1H, H_Art_), 5.17 (s, 2H, H_CH2_), 4.78 (d, *J* = 3.5 Hz, 1H, H_Art_), 4.52–4.41 (m, 4H, H_CH2_), 3.81 (t, *J* = 5.6 Hz, 1H, H_CH2_), 3.50 (d, *J* = 10.0 Hz, 2H, H_CH2_), 2.65 (s, 1H, H_Art_), 2.21–2.09 (m, 1H, H_Art_), 2.09–2.00 (m, 2H, H_Art_), 1.66–1.64 (m, 6H, H_Art_), 1.45 (s, 2H, H_Art_), 1.35 (sl, 1H, H_Art_), 1.25 (t, *J* = 7.0 Hz, 1H, H_Art_), 0.98 (d, *J* = 6.1 Hz, 3H, H_Art_), 0.96–0.94 (m, 1H, H_Art_), 0.91 (d, *J* = 7.4 Hz, 3H, H_Art_). ^13^C NMR (101 MHz, CDCl_3_): δ 137.3 (1C, C_2_), 129.7 (2C, C_Bn_), 129.1 (2C, C_Bn_), 128.6 (1C, C_Bn_), 122.1 (1C, C_4_), 121.4 (1C, C_5_), 120.6 (1C, C_Bn_), 102.1 (1C, C_Art_), 94.0 (1C, C_Art_), 87.9 (1C, C_Art_), 61.1 (1C, C_Art_), 56.5 (1C, C_CH2_), 53.6 (1C, C_Bn_), 51.7 (1C, C_Art_), 50.0 (1C, C_Art_), 45.3 (1C, C_Art_), 41.2 (1C, C_Art_), 40.3 (1C, C_Art_), 34.5 (1C, C_Art_), 30.3 (1C, C_Art_), 30.0 (1C, C_CH2_), 29.5 (1C, C_CH2_), 28.3 (1C, C_Art_), 27.5 (1C, C_Art_), 20.5 (1C, C_CH2_), 20.0 (1C, C_Art_), 11.1 (1C, C_Art_). HRMS (ESI^+^): calcd. for C_29_H_41_N_2_O_5_ *m/z* = 497.3010, found 497.3007.

*3′-Mesityl-1′-[10β-(21-butoxy)dihydroartemisinin]1H-imidazol-3-ium bromide* (**L4-Mes** (**8**)). To a stirred solution of **DHA-C4** (189 mg, 0.45 mmol) in CH_3_CN (4 mL) heated at 70 °C, 1-mesitylimidazole (142 mg, 0.76 mmol) was added and the reaction mixture was stirred for 3 days. The solvent was removed under reduced pressure and the crude product was dissolved in CH_2_Cl_2_ and precipitated with Et_2_O. This treatment was repeated three times to afford a white solid (168 mg, 62% yield). Anal. Calcd. for C_31_H_45_BrN_2_O_5_: C, 61.48; H, 7.49; N, 4.63. Found C, 62.21; H, 7.66; N, 4.36. ^1^H-NMR (300 MHz, CDCl_3_): δ 10.55 (t, *J* = 1.4 Hz, 1H, H_2_), 7.68 (t, *J* = 1.9 Hz, 1H, H_4_), 7.15 (t, *J* = 1.9 Hz, 1H, H_5_), 7.03 (s, 2H, H_Mes_), 5.42 (s, 1H, H_Art_), 5.02 (s, 1H, H_Art_), 4.86 (d, *J* = 3.3 Hz, 2H, H_CH2_), 3.94 (dt, *J* = 9.7, 6.2 Hz, 1H, H_CH2_), 3.63 (dt, *J* = 9.7, 6.2 Hz, 1H, H_CH2_), 2.37 (s, 3H, H_Mes_), 2.36–2.24 (m, 1H, H_Art_), 2.19 (s, 1H, H_Art_), 2.11 (s, 6H, H_Mes_), 2.10–2.01 (m, 1H, H_Art_), 1.95–1.87 (m, 1H, H_CH2_), 1.80–1.75 (m, 2H, H_CH2_), 1.72-1.66 (m, 2H, H_Art_), 1.64–1.60 (m, 3H, H_Art_), 1.57–1.52 (m, 1H, H_CH2_), 1.50–1.43 (m, 1H, H_Art_), 1.42 (s, 3H, H_Art_), 1.40–1.36 (m, 1H, H_Art_), 1.31–1.26 (m, 1H, H_Art_), 1.18 (sl, 2H, H_Art_), 1.16, (sl, 1H, H_Art_), 1.10–1.01 (m, 1H, H_Art_), 1.00–0.94 (m, 3H, H_Art_), 0.93–0.87 (m, 1H, H_Art_). ^13^C NMR (101 MHz, CDCl_3_): δ 139.1 (1C, C_Mes_), 138.9 (1C, C_2_), 136.7 (2C, C_Mes_), 129.3 (2C, C_Mes_), 127.5 (1C, C_Mes_), 123.0 (1C, C_4_), 120.6 (1C, C_5_), 104.1 (1C, C_Art_), 102.2 (1C, C_Art_), 87.9 (1C, C_Art_), 81.1 (1C, C_Art_), 67.7 (1C, C_CH2_), 52.5 (1C, C_Art_), 50.3 (1C, C_CH2_), 44.4 (1C, C_Art_), 37.5 (1C, C_Art_), 36.4 (1C, C_Art_), 34.5 (1C, C_Art_), 30.9 (1C, C_Art_), 27.4 (1C, C_CH2_), 26.2 (1C, C_Art_), 25.0 (1C, C_Art_), 24.5 (1C, C_Art_), 21.2 (1C, C_CH2_), 20.4 (1C, C_Art_), 17.6 (2C, C_Mes_), 17.3 (1C, C_Mes_), 13.2 (1C, C_Art_). HRMS (ESI^+^): calcd. for C_31_H_45_N_2_O_5_ *m/z* = 525.3328, found 525.3353.

*3′-Quinolin-2-yl-1′-[10β-(21-butoxy)dihydroartemisinin]1H-imidazol-3-ium bromide* (**L4-Quin** (**9**)). To a stirred solution of **DHA-C4** (128 mg, 0.31 mmol) in CH_3_CN (3 mL) heated at 70 °C, 1-(quinolin-2-yl)-imidazole (78 mg, 0.40 mmol) was added and the reaction mixture was stirred for 3 days. Then, the solvent was removed under reduced pressure and the crude product was dissolved in CH_2_Cl_2_ and precipitated with Et_2_O. This treatment was repeated three times to afford a white solid (111 mg, 58% yield). Anal. Calcd. for C_31_H_40_BrN_3_O_5_: C, 60.58; H, 6.56; N, 6.84. Found C, 60.45; H, 6.72; N, 6.75. ^1^H-NMR (300 MHz, CDCl_3_): δ 12.03 (s, 1H, H_2_), 8.69–8.63 (m, 1H, H_Quin_), 8.58–8.50 (m, 2H, H_Quin_, H_4_), 8.04 (d, *J* = 8.7 Hz, 1H, H_Quin_), 7.88–7.74 (m, 2H, H_Quin_, H_5_), 7.70–7.57 (m, 1H, H_Quin_), 7.57 (sl, 1H, H_Quin_), 5.38 (s, 1H, H_Art_), 4.78 (d, *J* = 3.7 Hz, 1H, H_Art_), 4.70 (t, *J* = 7.3 Hz, 2H, H_CH2_), 3.97–3.85 (m, 1H, H_CH2_), 3.49–3.47 (m, 1H, H_CH2_), 2.66–2.58 (m, 1H, H_Art_), 2.44–2.30 (m, 1H, H_Art_), 2.20–2.11 (m, 2H, H_Art_), 2.02–1.96 (m, 1H, H_Art_), 1.88–1.82 (m, 1H, H_Art_), 1.81–1.64 (m, 4H, H_CH2_), 1.42 (s, 2H, H_Art_), 1.38 (s, 3H, H_Art_), 1.28–1.25 (m, 1H, H_Art_), 1.21–1.20 (m, 1H, H_Art_), 0.97 (d, *J* = 6.4 Hz, 3H, H_Art_), 0.96–0.93 (m, 1H, H_Art_), 0.90 (d, *J* = 7.0, 3H, H_Art_). ^13^C NMR (101 MHz, CDCl_3_): δ 146.1 (1C, C_Quin_), 144.5 (1C, C_Quin_), 141.7 (1C, C_Quin_), 140.3 (1C, C_Quin_), 137.0 (1C, C_2_), 131.5 (1C, C_Quin_), 128.8 (1C, C_Quin_), 128.2 (1C, C_4_), 127.8 (1C, C_Quin_), 121.6 (1C, C_5_), 119.0 (1C, C_Quin_), 112.9 (1C, C_Quin_), 104.2 (1C, C_Art_), 102.3 (1C, C_Art_), 88.0 (1C, C_Art_), 81.1 (1C, C_Art_), 65.9 (1C, C_CH2_), 52.5 (1C, C_Art_), 50.4 (1C, C_Art_), 44.3 (1C, C_Art_), 37.5 (1C, C_Art_), 36.4 (1C, C_Art_), 34.5 (1C, C_Art_), 30.9 (1C, C_Art_), 27.3 (1C, C_CH2_), 26.5 (1C, C_CH2_), 26.2 (1C, C_Art_), 24.7 (1C, C_Art_), 20.3 (1C, C_Art_), 15.3 (1C, C_CH2_), 13.1 (1C, C_Art_). HRMS (ESI^+^): calcd. for C_31_H_40_N_3_O_5_ *m/z* = 534.2962, found 534.2976.

*Complex***Aubis(4-Bn)** (**20**). Under a nitrogen atmosphere, K_2_CO_3_ (36 mg, 0.26 mmol) was added to **L4-Bn** (108 mg, 0.19 mmol) in dry CH_3_CN (4 mL) and heated at 60 °C under stirring. Then, **Au(SMe_2_)Cl** (25 mg, 0.09 mmol) was added and the mixture was stirred for 10 h. After cooling to room temperature, the solution was filtered through a pad of celite and the solvent removed under reduced pressure. The complex was purified by flash chromatography on silica with CH_2_Cl_2_-MeOH as eluent (100/0 to 100/10) to give a white solid (100 mg, 98% yield). Anal. Calcd. for C_58_H_80_AuClN_4_O_10_: C, 56.84; H, 6.58; N, 4.57. Found C, 56.75; H, 6.46; N, 4.51. ^1^H-NMR (300 MHz, CDCl_3_): δ 7.35–7.31 (m, 5H, H_Bn_), 7.26 (sl, 2H, H_4_, H_5_), 5.45 (sl, 2H, H_Bn_), 5.36 (s, 1H, H_Art_), 5.32 (s, 1H, H_CH2_), 4.77–4.71 (m, 1H, H_Art_), 4.26 (t, *J* = 7.1 Hz, 2H, H_CH2_), 3.81 (dt, *J* = 9.9, 6.3 Hz, 1H, H_CH2_), 3.37 (dt, *J* = 9.9, 6.3 Hz, 2H, H_CH2_), 2.66–2.58 (m, 1H, H_Art_), 2.45–2.32 (m, 1H, H_Art_), 2.16–2.11 (m, 1H, H_Art_), 2.10–2.00 (m, 1H, H_Art_), 1.97–1.86 (m, 3H, H_CH2_, H_Art_), 1.78–1.68 (m, 2H, H_Art_), 1.65–1.54 (m, 2H, H_Art_), 1.44 (s, 3H, H_Art_), 1.34–1.24 (m, 2H, H_Art_), 0.96 (d, *J* = 5.8 Hz, 3H, H_Art_), 0.90 (d, *J* = 2.2 Hz, 1H, H_Art_), 0.87 (d, *J* = 2.5 Hz, 3H, H_Art_). ^13^C NMR (75 MHz, CDCl_3_): δ 183.6 (1C, C_2_), 135.7 (1C, C_Bn_), 129.1 (2C, C_Bn_), 128.6 (1C, C_Bn_), 127.4 (2C, C_Bn_), 122.4 (1C, C_4_), 121.8 (1C, C_5_), 104.2 (1C, C_Art_), 102.0 (1C, C_Art_), 87.9 (1C, C_Art_), 81.0 (1C, C_Art_), 67.5 (1C, C_Art_), 54.8 (1C, C_Bn_), 52.5 (1C, C_Art_), 51.3 (1C, C_CH2_), 44.3 (1C, C_Art_), 37.5 (1C, C_Art_), 36.4 (1C, C_Art_), 34.6 (1C, C_Art_), 30.8 (1C, C_Art_), 29.7 (1C, C_CH2_), 28.2 (1C, C_CH2_), 26.2 (1C, C_Art_), 24.7 (1C, C_Art_), 24.5 (1C, C_Art_), 22.3 (1C, C_CH2_), 13.1 (1C, C_Art_). HRMS (ESI^+^): calcd. for C_58_H_80_AuN_4_O_10_ *m/z* = 1189.5534, found 1189.5468.

*Complex***Aubis(4-Mes)** (**21**). Under a nitrogen atmosphere, K_2_CO_3_ (17 mg, 0.13 mmol) was added to **L4-Mes** (55 mg, 0.09 mmol) in dry CH_3_CN (4 mL). The mixture was then heated at 60 °C. After, **Au(SMe_2_)Cl** (15 mg, 0.05 mmol) was added and the mixture was stirred for 10 h. After cooling to room temperature, the solution was filtered through a pad of celite and the solvent removed under reduced pressure. The complex was purified by flash chromatography on silica with CH_2_Cl_2_-MeOH as eluent (100/0 to 100/10) to give a white solid (55 mg, 99% yield). Anal. Calcd. for C_62_H_88_AuClN_4_O_10_: C, 58.10; H, 6.92; N, 4.37. Found C, 58.19; H, 6.90; N, 4.37. ^1^H-NMR (300 MHz, CDCl_3_): δ 7.68 (t, *J* = 1.7 Hz, 1H, H_4_), 6.97–6.94 (m, 3H, H_5_, H_Mes_), 5.36 (s, 1H, H_Art_), 5.29 (s, 1H, H_Art_), 4.75 (d, *J* = 3.5 Hz, 1H, H_Art_), 4.10 (t, *J* = 6.9 Hz, 2H, H_CH2_), 3.79 (dt, *J* = 9.8, 6.3 Hz, 1H, H_CH2_), 3.34 (dt, *J* = 9.8, 6.4 Hz, 1H, H_CH2_), 2.67–2.60 (m, 1H, H_Art_), 2.38 (s, 6H, H_Mes_), 2.09–1.99 (m, 2H, H_Art_), 1.87 (d, *J* = 2.3 Hz, 9H, H_CH2_, H_Art_), 1.75 (sl, 3H, H_Mes_), 1.68–1.61 (m, 2H, H_CH2_), 1.43 (s, 3H, H_Art_), 0.97 (d, *J* = 5.8 Hz, 3H, H_Art_), 0.90 (d, *J* = 7.3 Hz, 4H, H_Art_). ^13^C NMR (101 MHz, CDCl_3_): δ 183.9 (1C, C_2_), 139.6 (1C, C_Mes_), 134.9 (2C, C_Mes_), 134.8 (1C, C_Mes_), 129.2 (2C, C_Mes_), 122.6 (1C, C_4_), 122.5 (1C, C_5_), 102.9 (1C, C_Art_), 102.7 (1C, C_Art_), 89.4 (1C, C_Art_), 81.8 (1C, C_Art_), 68.1 (1C, C_CH2_), 51.7 (1C, C_Art_), 46.7 (1C, C_CH2_), 40.2 (1C, C_Art_), 37.3 (1C, C_Art_), 36.5 (1C, C_Art_), 34.3 (1C, C_Art_), 31.7 (1C, C_Art_), 28.3 (1C, C_CH2_), 26.6 (1C, C_Art_), 26.0 (1C, C_Art_), 24.7 (1C, C_Art_), 21.2 (1C, C_CH2_), 20.0 (1C, C_Mes_), 19.4 (1C, C_Art_), 17.6 (2C, C_Mes_), 15.3 (1C, C_Art_). HRMS (ESI^+^): calcd. for C_62_H_88_AuN_4_O_10_ *m/z* = 1245.6166, found 1245.6188.

*Complex***Aubis(4-Quin)** (**22**). Under a nitrogen atmosphere, K_2_CO_3_ (28 mg, 0.20 mmol) was added to **L4-Quin** (89 mg, 0.14 mmol) in dry CH_3_CN (4 mL). The stirred mixture was then heated at 60 °C. Then, **Au(SMe_2_)Cl** (30 mg, 0.10 mmol) was added and the mixture was stirred for 10 h. After cooling to room temperature, the solution was filtered through a pad of celite and the solvent removed under reduced pressure to give a white solid (84 mg, 92% yield). Anal. Calcd. For C_62_H_78_AuClN_6_O_10_: C, 57.29; H, 6.05; N, 6.47. Found C, 57.35; H, 6.01; N, 6.57. ^1^H-NMR (400 MHz, CDCl_3_): δ 8.41–8.29 (m, 1H, H_Quin_), 8.20–8.14 (m, 1H, H_Quin_), 8.09–8.05 (m, 1H, H_4_), 7.90–7.85 (m, 1H, H_Quin_), 7.79–7.73 (m, 1H, H_Quin_), 7.71–7.64 (m, 1H, H_Quin_), 7.57–7.47 (m, 2H, H_5_, H_Quin_), 5.35–5.32 (m, 1H, H_Art_), 4.77–4.65 (m, 1H, H_Art_), 4.46–4.45 (m, 2H, H_CH2_), 3.93–3.91 (m, 1H, H_CH2_), 3.81–3.74 (m, 1H, H_CH2_), 3.29–3.35 (m, 2H, H_CH2_), 2.63–2.62 (m, 1H, H_Art_), 2.42–2.32 (m, 1H, H_Art_), 2.15–2.11 (m, 2H, H_CH2_), 2.07–1.96 (m, 1H, H_Art_), 1.91–1.82 (m, 1H, H_Art_), 1.74–1.66 (m, 2H, H_Art_), 1.64–1.55 (m, 1H, H_Art_), 1.43 (s, 1H, H_Art_), 1.34–1.25 (m, 1H, H_Art_), 1.25–1.21 (m, 3H, H_Art_), 1.20–1.98 (m, 1H, H_Art_), 1.22–1.16 (m, 1H, H_Art_), 0.95 (d, *J* = 6.2 Hz, 3H, H_Art_), 0.87–0.85 (m, 4H, H_Art_). ^13^C NMR (126 MHz, CDCl_3_): δ 181.9 (1C, C_2_), 149.2 (1C, C_Quin_), 146.3 (1C, C_Quin_), 139.8 (1C, C_Quin_), 131.0 (1C, C_Quin_), 128.5 (1C, C_Quin_), 127.9 (1C, C_Quin_), 127.7 (1C, C_Quin_), 127.5 (1C, C_Quin_), 122.4 (1C, C_4_), 121.6 (1C, C_5_), 116.1 (1C, C_Quin_), 104.1 (1C, C_Art_), 102.0 (1C, C_Art_), 87.9 (1C, C_Art_), 81.0 (1C, C_Art_), 67.8 (1C, C_CH2_), 65.9 (1C, C_CH2_), 52.5 (C1, C_Art_), 52.3 (1C, C_Art_), 44.3 (1C, C_Art_), 37.5 (1C, C_Art_), 36.4 (1C, C_Art_), 34.5 (1C, C_Art_), 30.8 (1C, C_Art_), 28.2 (1C, C_CH2_), 26.2 (1C, C_Art_), 24.7 (1C, C_Art_), 20.3 (1C, C_Art_), 15.3 (1C, C_CH2_), 13.1 (1C, C_Art_). HRMS (ESI^+^): calcd. for C_62_H_78_AuN_6_O_10_ *m/z* = 1263.5439, found 1263.5468.

*3′-Benzyl-1′-[10β-(22-pentoxy)dihydroartemisinin]1H-imidazol-3-ium bromide* (**L5-Bn** (**10**)). To a stirred solution of **DHA-C5** (103 mg, 0.24 mmol) in CH_3_CN (4 mL) heated at 70 °C, 1-benzylimidazole (34 mg, 0.21 mmol) was added and the reaction mixture was stirred 3 days. Then, the solvent was removed under reduced pressure and the crude product was dissolved in CH_2_Cl_2_ and precipitated with Et_2_O. This treatment was repeated three times to afford a white solid (64 mg, 52% yield). Anal. Calcd. for C_30_H_43_BrN_2_O_5_: C, 60.91; H, 7.33; N, 4.74. Found C, 60.91; H, 7.45; N, 4.68. ^1^H-NMR (300 MHz, CDCl_3_): δ 10.73 (t, *J* = 1.6 Hz, 1H, H_2_), 7.54–7.46 (m, 2H, H_Bn_, H_4_/H_5_), 7.41–7.35 (m, 5H, H_Bn_, H_4_/H_5_), 5.63 (s, 2H, H_Bn_), 5.35 (s, 1H, H_Art_), 4.75–4.71 (m, 1H, H_Art_), 4.32–4.28 (m, 2H, H_CH2_), 3.85–3.76 (m, 1H, H_CH2_), 3.38–3.30 (m, 1H, H_CH2_), 2.65–2.53 (m, 1H, H_Art_), 2.41–2.30 (m, 1H, H_Art_), 2.07–1.90 (m, 3H, H_Art_), 1.89–1.82 (m, 1H, H_CH2_), 1.78–1.68 (m, 3H, H_CH2_, H_Art_), 1.66–1.57 (m, 4H, H_CH2_, H_Art_), 1.41 (s, 3H, H_Art_), 1.35–1.10 (m, 3H, H_Art_), 0.95 (d, *J* = 6.1 Hz, 4H, H_Art_), 0.85 (d, *J* = 7.3 Hz, 3H, H_Art_). ^13^C NMR (101 MHz, CDCl_3_): δ 137.9 (1C, C_2_), 135.0 (1C, C_Bn_), 129.6 (1C, C_Bn_), 129.5 (2C, C_Bn_), 129.1 (2C, C_Bn_), 121.4 (1C, C_4_), 121.3 (1C, C_5_), 104.1 (1C, C_Art_), 102.1 (1C, C_Art_), 89.7 (1C, C_Art_), 81.1 (1C, C_Art_), 67.9 (1C, C_CH2_), 53.6 (1C, C_Bn_), 52.6 (1C, C_Art_), 50.0 (1C, C_Art_), 44.4 (1C, C_Art_), 37.5 (1C, C_Art_), 36.4 (1C, C_Art_), 34.6 (1C, C_Art_), 30.9 (1C, C_CH2_), 30.0 (1C, C_Art_), 29.3 (1C, C_CH2_), 29.0 (1C, C_CH2_), 26.2 (1C, C_Art_), 24.7 (1C, C_Art_), 22.2 (1C, C_CH2_), 20.4 (1C, C_Art_), 13.1 (1C, C_Art_). HRMS (ESI^+^): calcd. for C_30_H_43_N_2_O_5_ *m/z* = 511,3172, found 511.3171.

*3′-Mesityl-1′-[10β-(22-pentoxy)dihydroartemisinin]1H-imidazol-3-ium bromide* (**L5-Mes** (**11**)). To a stirred solution of **DHA-C5** (160 mg, 0.37 mmol) in CH_3_CN (4 mL) heated at 70 °C, 1-mesitylimidazole (51 mg, 0.27 mmol) was added and the reaction mixture was stirred for 3 days. The solvent was removed under reduced pressure and the crude product was dissolved in CH_2_Cl_2_ and precipitated with Et_2_O. This treatment was repeated three times to afford a white solid (101 mg, 60% yield). Anal. Calcd. for C_32_H_47_BrN_2_O_5_: C, 62.03; H, 7.65; N, 4.52. Found C, 62.15; H, 7.75; N, 4.62. ^1^H-NMR (400 MHz, CDCl_3_): δ 10.64 (t, *J* = 1.5 Hz, 1H, H_2_), 7.58 (t, *J* = 1.7 Hz, 1H, H_4_), 7.16 (t, *J* = 1.8 Hz, 1H, H_5_), 7.03 (sl, 2H, H_Mes_), 5.40 (s, 1H, H_Art_), 4.87–4.71 (m, 3H, H_Art_, H_CH2_), 3.85 (dt, *J* = 9.8, 6.4 Hz, 1H, H_CH2_), 3.41 (dt, *J* = 9.8, 6.4 Hz, 1H, H_CH2_), 2.69–2.59 (m, 1H, H_Art_), 2.44–2.34 (m, 4H, H_Mes_, H_Art_), 2.11 (s, 6H, H_Mes_), 2.10–2.01 (m, 3H, H_Art_), 1.94–1.87 (m, 1H, H_CH2_), 1.76–1.74 (m, 2H, H_CH2_, H_Art_), 1.72–1.66 (m, 2H, H_CH2_), 1.64 (s, 2H, H_CH2_), 1.55–1.47 (m, 3H, H_Art_), 1.45 (s, 3H, H_Art_), 1.41–1.31 (m, 1H, H_Art_), 1.30–1.23 (m, 1H, H_Art_), 0.98 (d, *J* = 6.2 Hz, 3H, H_Art_), 0.94–0.92 (m, 1H, H_Art_), 0.90 (d, *J* = 7.4 Hz, 3H, H_Art_). ^13^C NMR (101 MHz, CDCl_3_): δ 141.5 (1C, C_Mes_), 138.7 (1C, C_2_), 134.2 (2C, C_Mes_), 130.6 (1C, C_Mes_), 130.0 (2C, C_Mes_), 122.9 (1C, C_4_), 122.0 (1C, C_5_), 104.1 (1C, C_Art_), 102.1 (1C, C_Art_), 88.0 (1C, C_Art_), 81.1 (1C, C_Art_), 68.0 (1C, C_CH2_), 52.6 (1C, C_Art_), 50.5 (1C, C_CH2_), 44.4 (1C, C_Art_), 37.5 (1C, C_Art_), 36.4 (1C, C_Art_), 34.6 (1C, C_Art_), 30.9 (1C, C_Art_), 30.4 (1C, C_CH2_), 29.1 (1C, C_CH2_), 26.2 (1C, C_Art_), 24.7 (1C, C_Art_), 24.5 (1C, C_Art_), 23.0 (1C, C_CH2_), 21.1 (1C, C_Art_), 20.4 (1C, C_Mes_), 17.7 (2C, C_Mes_), 13.1 (1C, C_Art_). HRMS (ESI^+^): calcd. for C_32_H_47_N_2_O_5_ *m/z* = 539.3485, found 539.3490.

*3′-Quinolin-2-yl-1′-[10β-(22-pentoxy)dihydroartemisinin]1H-imidazol-3-ium bromide* (**L5-Quin** (**12**)). To a stirred solution of **DHA-C5** (142 mg, 0.33 mmol) in CH_3_CN (3 mL) heated at 70 °C, 1-(quinolin-2-yl)-imidazole (84 mg, 0.43 mmol) was added and the reaction mixture was stirred for 3 days. Then, the solvent was removed under reduced pressure and the crude product was dissolved in CH_2_Cl_2_ and precipitated with Et_2_O. This treatment was repeated three times to afford a white solid (136 mg, 66% yield). Anal. Calcd. for C_32_H_42_BrN_3_O_5_: C, 61.14; H, 6.73; N, 6.68. Found C, 61.18; H, 6.67; N, 6.69. ^1^H-NMR (300 MHz, CDCl_3_): δ 11.99 (s, 1H, H_2_), 8.67–8.64 (m, 1H, H_Quin_), 8.55–8.52 (m, 1H, H_Quin_), 8.34–8.31 (m, 1H, H_4_), 8.05–8.02 (m, 1H, H_Quin_), 7.95–7.90 (m, 1H, H_Quin_), 7.88–7.83 (m, 1H, H_Quin_), 7.84–7.77 (m, 1H, H_Quin_), 7.59–7.53 (m, 1H, H_5_), 5.39–5.36 (m, 1H, H_Art_), 4.78–4.75 (m, 1H, H_Art_), 4.66–4.61 (m, 2H, H_CH2_, H_Art_), 3.84–3.80 (m, 1H, H_CH2_), 3.69–3.65 (m, 1H, H_CH2_), 3.46–3.31 (m, 3H, H_CH2_), 2.69–2.52 (m, 1H, H_Art_), 2.44–2.29 (m, 2H, H_Art_), 2.19–2.08 (m, 2H, H_Art_), 1.93–1.78 (m, 2H, H_Art_), 1.75–1.67 (m, 4H, H_CH2_), 1.58–1.47 (m, 4H, H_Art_), 1.28–1.25 (m, 2H, H_Art_), 0.96 (d, *J* = 6.4 Hz, 3H, H_Art_), 0.95 (m, 1H, H_Art_), 0.93 (d, *J* = 7.4 Hz, 3H, H_Art_). ^13^C NMR (101 MHz, CDCl_3_): δ 146.1 (1C, C_Quin_), 144.5 (1C, C_Quin_), 141.6 (1C, C_Quin_), 136.8 (1C, C_2_), 131.5 (1C, C_Quin_), 129.0 (1C, C_Quin_), 128.8 (1C, C_Quin_), 128.3 (1C, C_Quin_), 128.1 (1C, C_Quin_), 122.2 (1C, C_4_), 119.0 (1C, C_5_), 112.8 (1C, C_Quin_), 104.3 (1C, C_Art_), 102.1 (1C, C_Art_), 87.9 (1C, C_Art_), 81.1 (1C, C_Art_), 67.9 (1C, C_CH2_), 51.0 (1C, C_Art_), 50.4 (1C, C_Art_), 44.4 (1C, C_Art_), 37.5 (1C, C_Art_), 36.4 (1C, C_Art_), 34.4 (1C, C_Art_), 31.1 (1C, C_CH2_), 30.0 (1C, C_Art_), 29.5 (1C, C_CH2_), 26.2 (1C, C_Art_), 26.2 (1C, C_CH2_), 23.1 (1C, C_Art_), 22.2 (1C, C_CH2_), 20.5 (1C, C_Art_), 13.1 (1C, C_Art_). HRMS (ESI^+^): calcd. for C_32_H_42_N_3_O_5_ *m/z* = 548.3119, found 548.3118.

*Complex***Aubis(5-Bn)** (**24**). Under a nitrogen atmosphere, K_2_CO_3_ (20 mg, 0.14 mmol) was added to **L5-Bn** (60 mg, 0.10 mmol) in dry CH_3_CN (4 mL) and heated at 60 °C under stirring. Then, **Au(SMe_2_)Cl** (15 mg, 0.05 mmol) was added and the mixture was stirred for 10 h. After cooling to room temperature, the solution was filtered through a pad of celite and the solvent removed under reduced pressure. The complex was purified by flash chromatography on silica with CH_2_Cl_2_-MeOH as eluent (100/0 to 100/10) to give a white solid (28 mg, 45% yield). Anal. Calcd. for C_60_H_84_AuClN_4_O_10_: C, 57.48; H, 6.75; N, 4.47. Found C, 57.58; H, 6.89; N, 4.32. ^1^H-NMR (300 MHz, CDCl_3_): δ 7.34–7.32 (m, 4H, H_Bn_), 7.26–7.25 (m, 3H, H_4_, H_5_), 5.42 (s, 2H, H_Bn_), 5.34 (s, 1H, H_Art_), 4.74 (m, 1H, H_Art_), 4.22–4.17 (m, 2H, H_CH2_), 3.81–3.74 (m, 1H, H_CH2_), 3.36–3.28 (m, 1H, H_CH2_), 2.66–2.56 (m, 1H, H_Art_), 2.42–2.32 (m, 1H, H_Art_), 2.10–2.00 (m, 1H, H_Art_), 1.96–1.82 (m, 3H, H_CH2_), 1.76–1.68 (m, 3H, H_Art_), 1.66–1.53 (m, 3H, H_CH2_), 1.50–1.45 (s, 1H, H_Art_), 1.40–1.18 (m, 6H, H_Art_), 0.96–0.94 (m, 3H, H_Art_), 0.91 (d, *J* = 7.4 Hz, 2H, H_Art_), 0.86 (d, *J* = 7.3 Hz, 3H, H_Art_). ^13^C NMR (101 MHz, CDCl_3_): δ 183.8 (1C, C_2_), 135.7 (1C, C_Bn_), 129.1 (2C, C_Bn_), 128.6 (1C, C_Bn_), 127.4 (2C, C_Bn_), 122.3 (1C, C_4_), 122.1 (1C, C_5_), 104.1 (1C, C_Art_), 101.9 (1C, C_Art_), 87.9 (1C, C_Art_), 81.1 (1C, C_Art_), 67.9 (1C, C_CH2_), 54.8 (1C, C_Bn_), 52.5 (1C, C_Art_), 51.5 (1C, C_CH2_), 44.4 (1C, C_Art_), 37.5 (1C, C_Art_), 36.4 (1C, C_Art_), 34.6 (1C, C_Art_), 31.2 (1C, C_CH2_), 30.9 (1C, C_Art_), 29.2 (1C, C_CH2_), 26.2 (1C, C_Art_), 24.7 (1C, C_Art_), 24.5 (1C, C_Art_), 23.3 (1C, C_CH2_), 20.4 (1C, C_Art_), 13.1 (1C, C_Art_). HRMS (ESI^+^): calcd. for C_60_H_84_AuN_4_O_10_ *m/z* = 1217.5847, found 1217.5889.

*Complex***Aubis(5-Mes)** (**25**). Under a nitrogen atmosphere, K_2_CO_3_ (36 mg, 0.26 mmol) was added to **L5-Mes** (101 mg, 0.16 mmol) in dry CH_3_CN (4 mL). The mixture was then heated at 60 °C. After, **Au(SMe_2_)Cl** (27 mg, 0.09 mmol) was added and the mixture was stirred for 10 h. After cooling to room temperature, the solution was filtered through a pad of celite and the solvent removed under reduced pressure. The complex was purified by flash chromatography on silica with CH_2_Cl_2_-MeOH as eluent (100/0 to 100/10) to give a white solid (78 mg, 74% yield). Anal. Calcd. for C_64_H_92_AuClN_4_O_10_: C, 58.69; H, 7.08; N, 4.28. Found C, 58.78; H, 7.02; N, 4.31. ^1^H-NMR (300 MHz, CDCl_3_): δ 7.66 (d, *J* = 1.8 Hz, 1H, H_4_), 6.97 (s, 2H, H_Mes_), 6.93 (d, *J* = 1.8 Hz, 1H, H_5_), 5.40 (s, 1H, H_Art_), 4.78 (d, *J* = 3.3 Hz, 1H, H_Art_), 4.11 (t, *J* = 6.9 Hz, 2H, H_CH2_), 3.80 (dt, *J* = 9.7, 6.9 Hz, 1H, H_CH2_), 3.34 (dt, *J* = 9.7, 6.9 Hz, 1H, H_CH2_), 2.65–2.63 (m, 1H, H_Art_), 2.40 (s, 3H, H_Mes_), 2.35 (m, 1H, H_Art_), 2.10–2.00 (m, 2H, H_Art_), 1.96–1.90 (m, 1H, H_Art_), 1.87 (d, *J* = 3.0 Hz, 6H, H_Mes_), 1.82–1.73 (m, 6H, H_CH2_), 1.72–1.60 (m, 3H, H_Art_), 1.56–1.49 (m, 1H, H_Art_), 1.45 (s, 4H, H_Art_), 1.33–1.26 (m, 1H, H_Art_), 0.98 (d, *J* = 6.0 Hz, 3H, H_Art_), 0.95–0.92 (m, 1H, H_Art_), 0.90 (d, *J* = 7.4 Hz, 3H, H_Art_). ^13^C NMR (101 MHz, CDCl_3_): δ 183.8 (1C, C_2_), 139.6 (1C, C_Mes_), 134.8 (2C, C_Mes_), 129.4 (1C, C_Mes_), 129.2 (2C, C_Mes_), 122.7 (1C, C_4_), 122.5 (1C, C_5_), 104.1 (1C, C_Art_), 102.0 (1C, C_Art_), 87.9 (1C, C_Art_), 81.1 (1C, C_Art_), 68.1 (1C, C_CH2_), 52.5 (1C, C_Art_), 51.1 (1C, C_CH2_), 44.4 (1C, C_Art_), 37.5 (1C, C_Art_), 36.4 (1C, C_Art_), 34.6 (1C, C_Art_), 31.1 (1C, C_CH2_), 30.9 (1C, C_Art_), 29.3 (1C, C_CH2_), 26.2 (1C, C_Art_), 24.7 (1C, C_Art_), 24.5 (1C, C_Art_), 23.0 (1C, C_CH2_), 21.2 (1C, C_Art_), 20.4 (2C, C_Mes_), 17.7 (1C, C_Mes_), 13.1 (1C, C_Art_). HRMS (ESI^+^): calcd. for C_64_H_92_AuN_4_O_10_ *m/z* = 1273.6473, found 1273.6495.

*Complex***Aubis(5-Quin)** (**26**). Under a nitrogen atmosphere, K_2_CO_3_ (37 mg, 0.27 mmol) was added to **L5-Quin** (121 mg, 0.19 mmol) in dry CH_3_CN (4 mL). The stirred mixture was then heated at 60 °C. Then, **Au(SMe_2_)Cl** (34 mg, 0.12 mmol) was added and the mixture was stirred for 10 h. After cooling to room temperature, the solution was filtered through a pad of celite and the solvent removed under reduced pressure to give a white solid (59 mg, 47% yield). Anal. Calcd. For C_64_H_82_AuClN_6_O_10_: C, 59.48; H, 6.40; N, 6.50. Found C, 59.45; H, 6.53; N, 6.45. ^1^H-NMR (300 MHz, CDCl_3_): δ 8.36 (m, 1H, H_Quin_), 8.18 (m, 1H, H_Quin_), 8.04 (m, 1H, H_4_), 7.89 (m, 1H, H_Quin_), 7.77 (m, 1H, H_Quin_), 7.71–7.65 (m, 1H, H_Quin_), 7.54 (m, 1H, H_5_), 7.51–7.48 (m, 1H, H_Quin_), 5.35–5.32 (m, 1H, H_Art_), 4.71 (m, 1H, H_Art_), 4.49–4.45 (m, 2H, H_Art_), 3.73–3.70 (m, 1H, H_CH2_), 3.53–3.46 (m, 3H, H_CH2_), 3.30–3.22 (m, 1H, H_CH2_), 2.63–2.57 (m, 1H, H_Art_), 2.42–2.32 (m, 1H, H_Art_), 2.13 (s, 1H, H_Art_), 2.02 (sl, 2H, H_Art_), 1.97–1.92 (m, 2H, H_Art_), 1.75–1.69 (m, 1H, H_CH2_), 1.61–1.59 (m, 1H, H_CH2_), 1.54–1.49 (m, 2H, H_CH2_), 1.44 (s, 3H, H_Art_), 1.34–1.27 (m, 3H, H_Art_), 0.94 (d, *J* = 5.9 Hz, 3H, H_Art_), 0.87 (m, 1H, H_Art_), 0.85 (d, *J* = 7.4 Hz, 3H, H_Art_). ^13^C NMR (75 MHz, CDCl_3_): δ 181.8 (1C, C_2_), 149.2 (1C, C_Quin_), 146.3 (1C, C_Quin_), 139.8 (1C, C_Quin_), 130.9 (1C, C_Quin_), 128.5 (1C, C_Quin_), 127.9 (1C, C_Quin_), 127.7 (1C, C_Quin_), 127.5 (1C, C_Quin_), 122.5 (1C, C_4_), 121.5 (1C, C_5_), 116.1 (1C, C_Quin_), 104.1 (1C, C_Art_), 101.9 (1C, C_Art_), 88.0 (1C, C_Art_), 81.0 (1C, C_Art_), 67.9 (1C, C_CH2_), 52.5 (C1, C_CH2_), 52.5 (1C, C_Art_), 50.6 (1C, C_CH2_), 44.4 (1C, C_Art_), 37.5 (1C, C_Art_), 36.4 (1C, C_Art_), 34.6 (1C, C_Art_), 31.0 (1C, C_CH2_), 30.8 (1C, C_Art_), 29.1 (1C, C_CH2_), 26.2 (1C, C_Art_), 24.7 (1C, C_Art_), 24.5 (1C, C_Art_), 20.4 (1C, C_Art_), 13.1 (1C, C_Art_). HRMS (ESI^+^): calcd. for C_64_H_82_AuN_6_O_10_ *m/z* = 1291.5758, found 1291.5785.

### 3.2. Biology

#### 3.2.1. Parasite Cultures

The *Plasmodium falciparum* F32-ART and F32-TEM parasite lines, resistant and sensitive to artemisinins respectively [9,15], were cultured in vitro in type O human erythrocytes (Etablissement Français du sang (EFS), Toulouse, France), diluted to 2% hematocrit, in RPMI-1640 medium (GIBCO, Illkirch, France) and supplemented with 5% Human Serum AB (EFS, Toulouse, France) at 37 °C and 5% CO_2_ [33]. Every other day and before each experiment, *P. falciparum* F32-ART and F32-TEM parasites were synchronized at ring stage using 5% D-sorbitol solution [34].

#### 3.2.2. Evaluation of Antiplasmodial Activity

In vitro antiplasmodial activities of the synthesized hybrid molecules were evaluated against the *P. falciparum* strain F32-TEM (corresponding to the parental strain F32-Tanzania). Each molecule was tested at least in three independent experiments (except **Aubis(5-Bn)**, tested twice) by a SYBR Green Fluorescence assay [33] but also at least once with the standard chemosensitivity assay recommended by the WHO and based on the incorporation of [^3^H] hypoxanthine [33].

For the SYBR Green Fluorescence assay, ring stage parasites were treated with the drugs for 48 h. Then, the pellets were washed twice with PBS and frozen at −20 °C. Thawed plates were incubated for 2 h at room temperature with the SYBR Green (Thermo-Fisher, Illkirch, France) lysis buffer (20 mm Tris base pH 7.5, 5 mm EDTA, 0.008% *w/v* saponin, 0.08% *w/v* Triton X-100). The plates were then read using a fluorescence plate reader (FLx800, BioTek, Illkirch, France) at an excitation wavelength of 485 nm and 535 nm for the emission wavelength. The IC_50_ values (concentration inhibiting 50% of the parasite’s growth) were determined using GraphPad Prism software 7 (GraphPad Software, San Diego, CA, USA).

For [^3^H] hypoxanthine assay, ring stage parasites were incubated with the drug dilutions for 24 h, then, [^3^H] hypoxanthine (50 μL/0.25 μCi; Perkin-Elmer, Courtaboeuf, France) was added for another 24 h period. After that, plates were frozen at −20 °C. The next step is to thaw the plates and to collect the nucleic acids. Tritium incorporation was then determined thanks to a β-counter (Perkin-Elmer, Courtaboeuf, France).

The antiplasmodial activities reported in Table 1 correspond thus to the mean of IC_50_ values from at least 4 independent experiments acquired by the two methods, SYBR Green and radioactivity.

#### 3.2.3. Evaluation of the Cytotoxicity

The most active compounds were tested for their cytotoxicity using the Vero cell line (monkey epithelial cell line), according to a previously described method [35] with some modifications. The cells were cultured in MEM medium (Dutscher, Brumath, France) supplemented with 10% fetal bovine serum (Dutscher), 0.7 mM glutamine and 100 μg/mL gentamicin.

The cells were seeded at 10^4^ cells per well in a 96-well plates and incubated during 24 h. Then, cells were incubated for additional 48 h with the drugs. Cell proliferation was measured with MTT (1-(4,5-dimethylthiazol-2-yl)-3,5-diphenylformazan, Sigma, Saint-Quentin Fallavier, France) which is added to each well for 1 h at 37° C and 5% CO_2_. Subsequently, DMSO was added to the wells containing the cells and MTT to dissolve the formed crystals. The plates were then read to determine the absorbance at wavelength of 540 nm (µQuant, BioTek, Illkirch, France). Cell proliferation was calculated from at least three independent experiments using GraphPad Prism software 7 (GraphPad Software, San Diego, CA, USA). The cytotoxic/antiplasmodial activity ratio corresponds to the selectivity index.

#### 3.2.4. Recrudescence Assay

*P. falciparum* strains F32-ART and F32-TEM, synchronized at ring stage parasites at 3% parasitemia and 2% hematocrit have undergone a 48 h-treatment with the drugs to be tested. The parasites were then washed with RPMI-1640 medium and re-cultivated in drug-free culture conditions with 10% human serum. The parasitemia was then monitored daily to determine the time required for each parasite culture to recover the initial parasitemia of 3%. If no parasites were seen up to 30 days, the parasites were considered as “not recrudescent” [9,15]. The drug doses were chosen in order to discriminate the phenotype response between the artemisinin-resistant and the -sensitive strains. A range from 1-fold to >100-fold the antiplasmodial IC_50_ value previously found has been tested to determine the most appropriate drug dose to use in this recrudescence assay.

## 4. Conclusions

Three original families of gold(I) NHCs complexes incorporating a covalently attached DHA derivative were synthetized and fully characterized. All the proligands and complexes were tested for their antiplasmodial potency on the *P. falciparum* strains, F32-TEM artemisinin-sensitive and F32-ART artemisinin-resistant. Among the 29 compounds tested, ten gold(I) complexes have shown high antiplasmodial activities, with IC_50_ values less than 50 nM and very low cytotoxicity, with selectivity indexes up to 294. However, even though a simple gold(I) bis(NHC) had shown in a previous work [20] no cross-resistance with artemisinins, the presence of this metal could not prevent cross-resistance in the hybrid gold(I)-DHA complexes **Aubis(3-Me)**, **Aubis(4-Me)** and **Aubis(5-Me)** tested. These data confirm that the presence of an endoperoxide moiety whatever the structure and the other parts of the hybrid molecules tested leads to artemisinins cross-resistance. Therefore, these findings raise concerns about the potential development of new artemisinin derivatives and more broadly endoperoxide-based antiplasmodial drugs even if they include a moiety metabolically active on biochemical pathways involved in the quiescence state.

## Figures and Tables

**Table:** 

Entry	Proligand	n	R	Yield (%)	Entry	Complex		n	R	Yield (%)
1 *	**L3-Me**	3	Me	65		14 *^,§^	**Aubis(3-Me)**	3	Me	84
**2**	**L3-iPr**	3	iPr	47		**15**	**Aubis(3-iPr)**	3	iPr	83
**3**	**L3-Bn**	3	Bn	87		**16**	**Aubis(3-Bn)**	3	Bn	65
**4**	**L3-Mes**	3	Mes	76		**17**	**Aubis(3-Mes)**	3	Mes	52
**5**	**L3-Quin**	3	Quin	84		**18**	**Aubis(3-Quin)**	3	Quin	86
6*	**L4-Me**	4	Me	98		19*	**Aubis(4-Me)**	4	Me	80
**7**	**L4-Bn**	4	Bn	73		**20**	**Aubis(4-Bn)**	4	Bn	92
**8**	**L4-Mes**	4	Mes	62		**21**	**Aubis(4-Mes)**	4	Mes	89
**9**	**L4-Quin**	4	Quin	58		**22**	**Aubis(4-Quin)**	4	Quin	92
10*	**L5-Me**	5	Me	35		23*	**Aubis(5-Me)**	5	Me	32
**11**	**L5-Bn**	5	Bn	52		**24**	**Aubis(5-Bn)**	5	Bn	45
**12**	**L5-Mes**	5	Mes	60		**25**	**Aubis(5-Mes)**	5	Mes	74
**13**	**L5-Quin**	5	Quin	66		**26**	**Aubis(5-Quin)**	5	Quin	47
						**27**	**Au(3-iPr)Cl**	3	Me	42
						**28**	**Au(3-Bn)Cl**	3	Bn	81
						**29**	**Au(3-Quin)Cl**	3	Quin	-

* From reference [25]. ^§^ Complex **Aubis(3-Me)** (**14**) was synthetized according to the transmetalation route [25].

**Table 1:** Antimalarial and cytotoxic activities of proligands and gold(I) complexes.

Entry	Compounds		Antiplasmodial Activity on *P. falciparum* IC_50_ ± SEM (nM)	Cytotoxicity on Vero CellsIC_50_ ± SEM (nM)	Selectivity Index Vero Cells/*P. falciparum*
**1**	Auranofin		1.5 × 10^3^ ± 0.1 × 10^3^		
**2**	Artemisinin		18 ± 2	130 × 10^3^	7 000
**3**	Artemether		6.1 ± 1	214 × 10^3^ ± 29^.^10^3^	35 000
**4**	DHA-C3		8.5 ± 3	2.5 × 10^3^ ± 0.1 × 10^3^	294
**5**	**L3-Me**	**1**	935 ± 117	-	-
**6**	**L3-iPr**	**2**	335 ± 28	-	-
**7**	**L3-Bn**	**3**	351 ± 56	-	-
**8**	**L3-Mes**	**4**	655 ± 53	-	-
**9**	**L3-Quin**	**5**	330 ± 69	-	-
**10**	**Aubis(3-Me)**	**14**	35 ± 8	5 × 10^3^ ± 1.5 × 10^3^	143
**11**	**Aubis(3-iPr)**	**15**	13 ± 5	0.7 × 10^3^ ± 0.2 × 10^3^	54
**12**	**Au(3-iPr)Cl**	**27**	45 ± 8	8 × 10^3^ ± 10^3^	178
**13**	**Aubis(3-Bn)**	**16**	22 ± 3	0.3 × 10^3^ ± 0.005 × 10^3^	14
**14**	**Au(3-Bn)Cl**	**28**	104 ± 24	8 × 10^3^ ± 2.3 × 10^3^	77
**15**	**Aubis(3-Mes)**	**17**	61 ± 37	0.5 × 10^3^ ± 0.1 × 10^3^	8
**16**	**Aubis(3-Quin)**	**18**	23 ± 8	1.2 × 10^3^ ± 0.5 × 10^3^	52
**17**	**Au(3-Quin)Cl**	**29**	90 ± 9	-	-
**18**	**L4-Me**	**6**	840 ± 64	-	-
**19**	**L4-Bn**	**7**	326 ± 44	-	-
**20**	**L4-Mes**	**8**	219 ± 18	-	-
**21**	**L4-Quin**	**9**	330 ± 37	-	-
**22**	**Aubis(4-Me)**	**19**	13 ± 3	0.8 × 10^3^ ± 0.2 × 10^3^	62
**23**	**Aubis(4-Bn)**	**20**	40 ± 21	0.7 × 10^3^ ± 0.2 × 10^3^	18
**24**	**Aubis(4-Mes)**	**21**	38 ± 3	0.7 × 10^3^ ± 0.1 × 10^3^	18
**25**	**Aubis(4-Quin)**	**22**	83 ± 31	-	-
**26**	**L5-Me**	**10**	172 ± 22	-	-
**27**	**L5-Bn**	**11**	98 ± 26	10^3^ ± 0.1 × 10^3^	10
**28**	**L5-Mes**	**12**	98 ± 24	25 × 10^3^ ± 10 × 10^3^	255
**29**	**L5-Quin**	**13**	226 ± 29	-	-
**30**	**Aubis(5-Me)**	**23**	9 ± 0.9	10^3^ ± 0.5 × 10^3^	111
**31**	**Aubis(5-Bn)**	**24**	72 ± 14	0.6 × 10^3^ ± 0.1 × 10^3^	8
**32**	**Aubis(5-Mes)**	**25**	100 ± 12	-	-
**33**	**Aubis(5-Quin)**	**26**	38 ± 9	2 × 10^3^ ± 0.7 × 10^3^	53

Values of the 50% inhibitory concentration (IC_50_) against *Plasmodium falciparum* were obtained using both SYBR Green and radioactivity assays. Cytotoxic activities of the compounds were determined against the Vero cell line. The antiplasmodial control drugs, artemether and artemisinin were routinely tested.

**Table 2:** Recrudescence capacity of *Plasmodium falciparum* F32-ART and F32-TEM strains after 48h-drug exposure.

Complexes	Doses	Number of Experiments	Median (range) Recrudescence Days		Mean ± SEM Difference of Recrudescence Days between F32-TEM and F32-ART
			F32-ART	F32-TEM	
Artemisinin	18 µM	6	9.5 (6–17)	18.5 (17–>30)	9.7 ± 0.3
**Aubis(4-Me) (14)**	100 nM	1	5	6	1
	500 nM	2	12.5 (11–14)	>30	>17.5 ± 1.5
	1 µM	1	16	30	14
**Aubis(5-Me) (19)**	100 nM	1	6	15	9
	500 nM	2	9.5 (8–11)	>25.5 (21–>30)	>16
	1 µM	1	9	30	21
**Aubis(3-Me) (23)**	200 nM	3	7 (7–13)	14 (11–16)	4.6 ± 1.2
	500 nM	1	8	24	16

Synchronized ring-stage parasites have undergone 48 h of drug treatment. After that, cultures were washed and parasitemia was monitored during 30 days or until reaching the initial parasitemia, defined as the recrudescence day. If no parasites were observed at the end of the experiment, the culture was classified as showing no recrudescence, and the recrudescence day was noted as >30.

## Supplementary Materials

Supplementary File 1
