# Supplementary material for: Hybrid Gold(I) NHC-Artemether Complexes to Target Falciparum Malaria Parasites
Source: Molecules. 2020 Jun 18;25(12):2817. doi: 10.3390/molecules25122817 (PMC7356589; doi:10.3390/molecules25122817)
Supplement: Supplementary File 1 [file molecules-25-02817-s001.pdf]

Hybrid Gold(I) NHC-Artemether Complexes to Target Falciparum Malaria Parasites

Manel Ouji[1,2,$] , Guillaume Barnoin[1,$], Álvaro Fernández Álvarez[1], Jean-Michel Augereau[1,2], Catherine Hemmert[1,*], Françoise Benoit-Vical[1,2,3,*] and Heinz Gornitzka[1,*]

Supporting Information:

^{1}H and ^{13}C NMR spectra

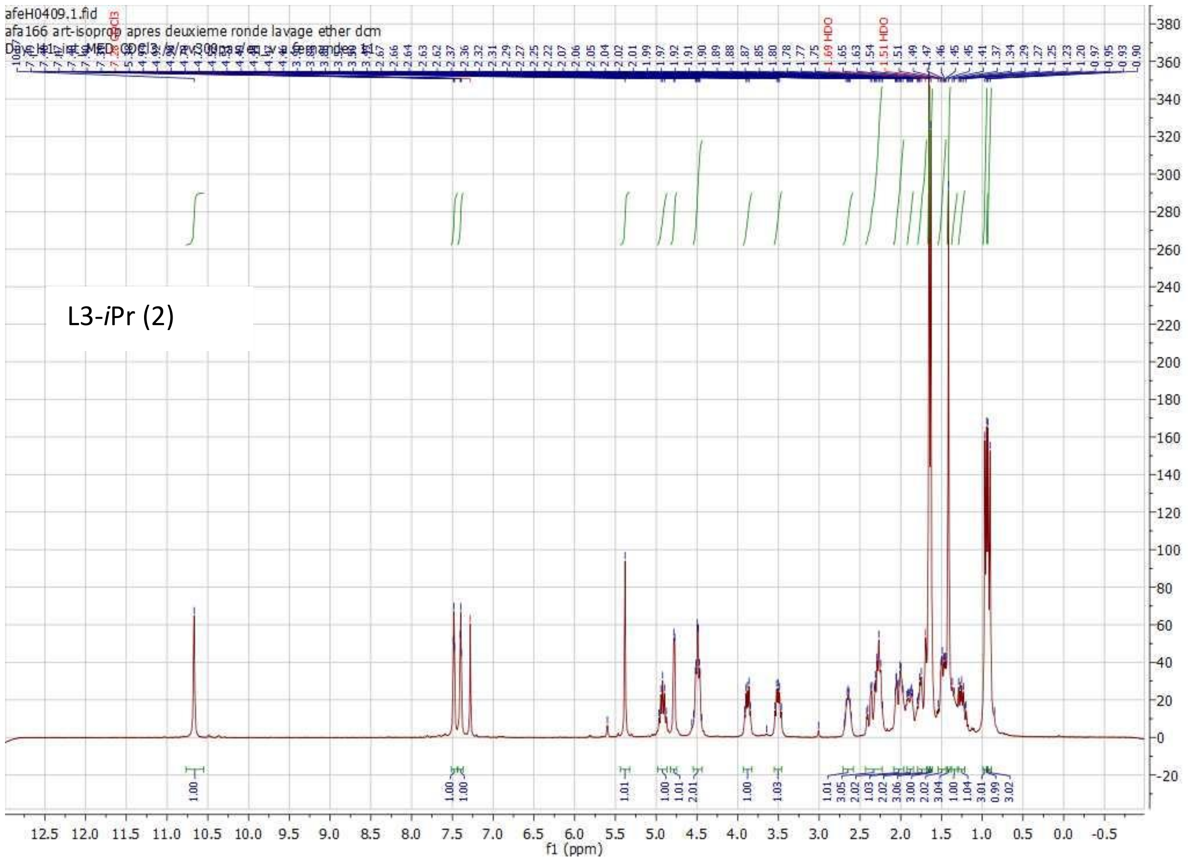
afeH0409.1.fid
afa166 art-isoprop apres deuxieme ronde lavage ether dcm
L3-iPr (2)
f1 (ppm)

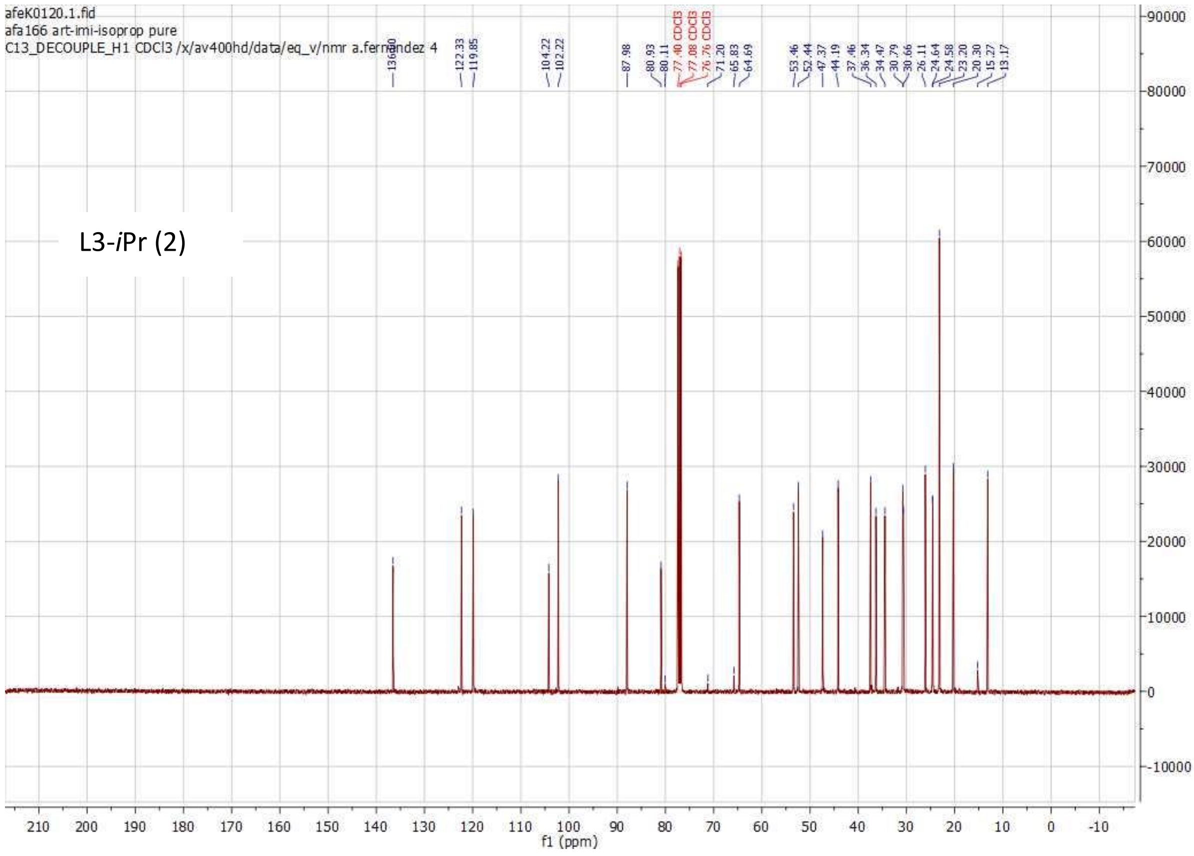
afeK0120.1.fid
afa166 art-imi-isoprop pure
C13_DECOUPLE_H1 CDCl3 /x/av400hd/data/eq_v/nmr a.fernandez 4
122.33
119.85
104.22
102.22
87.98
80.93
80.11
77.40 CDCl3
77.08 CDCl3
76.76 CDCl3
71.20
65.83
64.69
53.46
52.44
47.37
44.19
37.46
36.34
34.47
30.79
30.66
26.11
24.64
24.58
23.20
20.30
15.27
13.17
L3-iPr (2)
f1 (ppm)

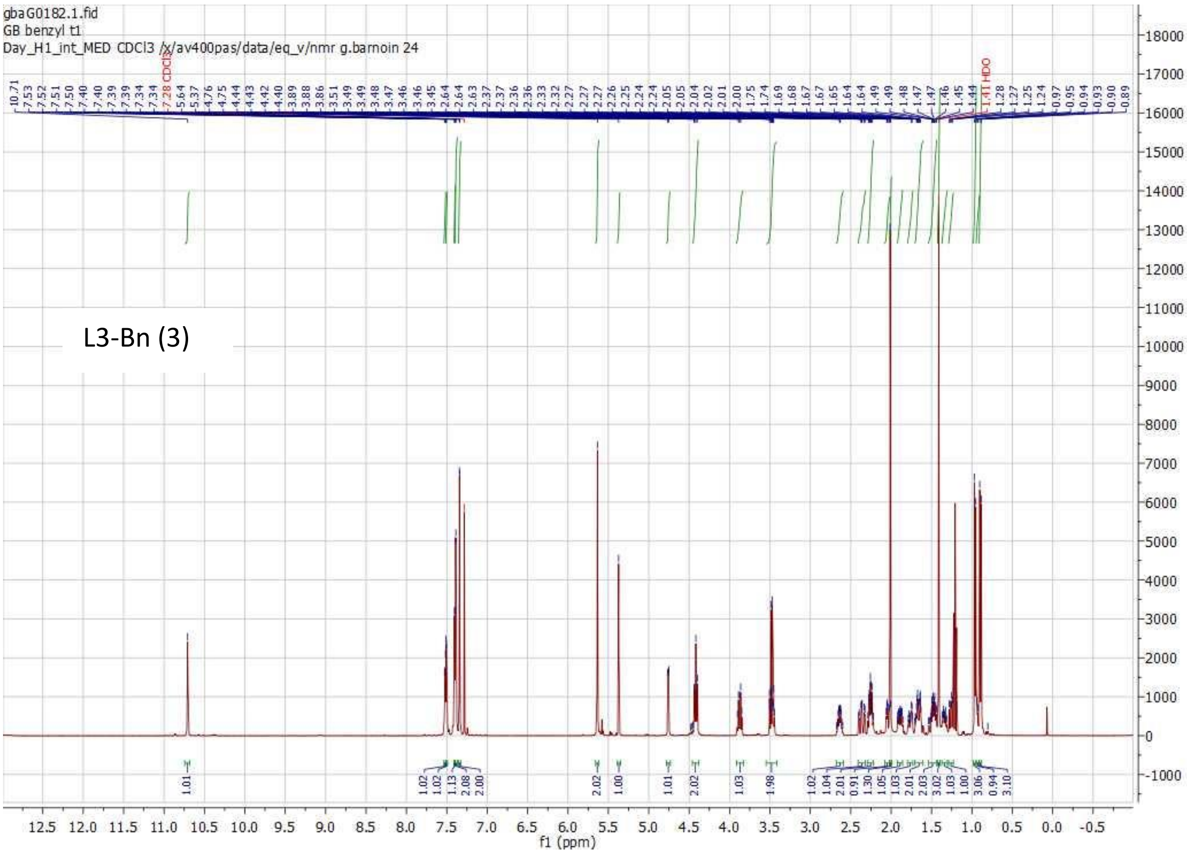
gbaG0182.1.fid
GB benzyl t1
Day_H1_int_MED CDCl3 /x/av400pas/data/eq_v/nmr g.barnoin 24
L3-Bn (3)
f1 (ppm)

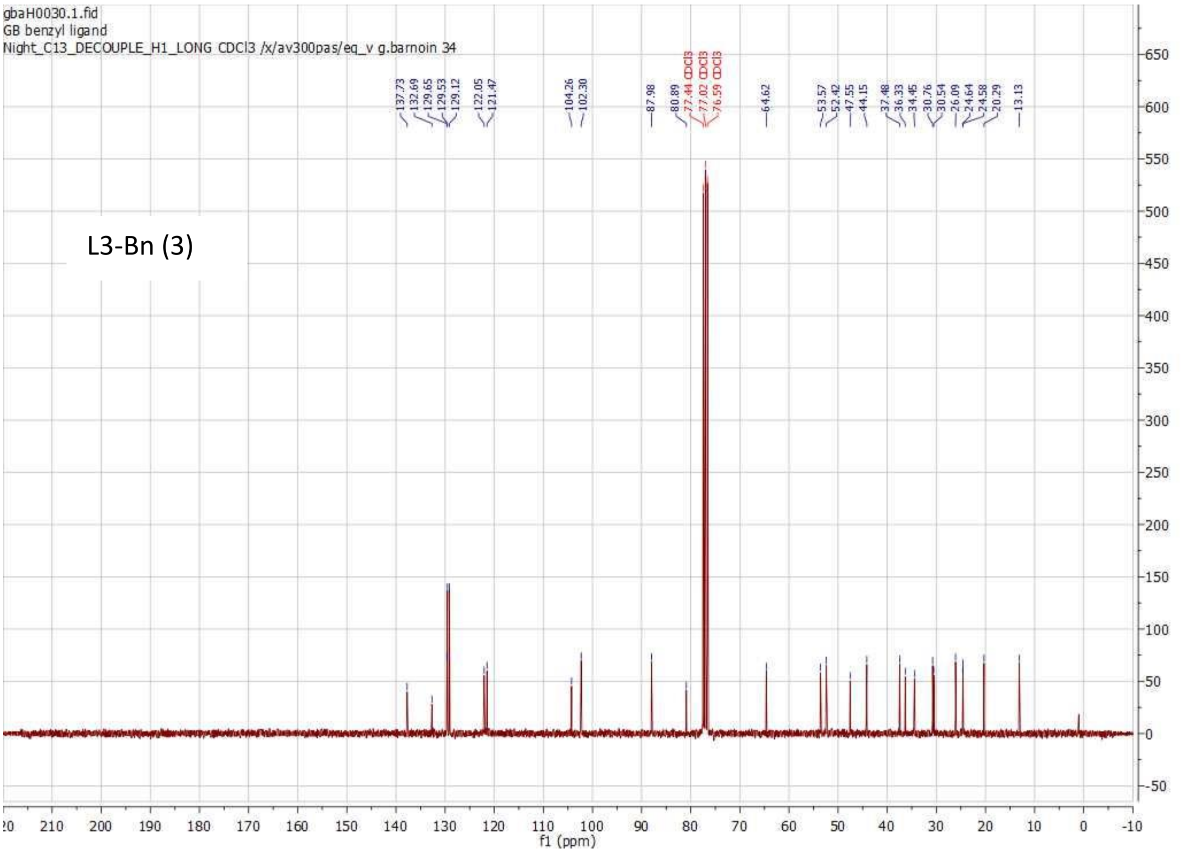
gbaH0030.1.fid
GB benzyl ligand
Night_C13_DECOUPLE_H1_LONG CDCl3 /x/av300pas/eq_v g.barnoin 34
137.73 132.69 129.65 129.53 129.12 122.05 121.47 104.26 102.30 87.98 80.89 77.44 CDCl3 77.02 CDCl3 76.59 CDCl3 64.62 53.57 52.42 47.55 44.15 37.48 36.33 34.45 30.76 30.54 26.09 24.64 24.58 20.29 13.13
L3-Bn (3)
f1 (ppm)

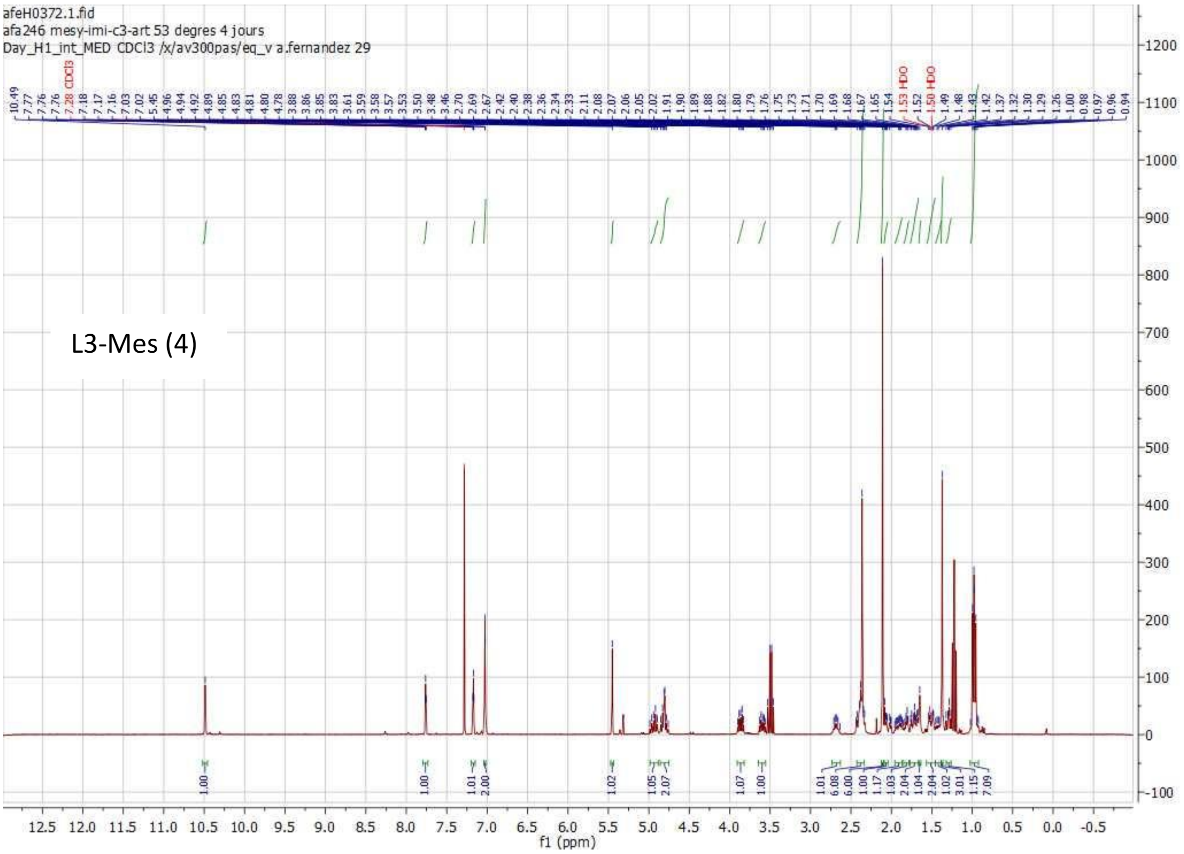
afeH0372.1.fid
afa246 mesy-imi-c3-art 53 degres 4 jours
Day_H1_int_MED CDCl3 /x/av300pas/eq_v a.fernandez 29
10.49 7.77 7.76 7.28 CDCl3 7.18 7.17 7.16 7.03 7.02 5.45 4.96 4.94 4.92 4.89 4.85 4.83 4.81 4.80 4.78 3.88 3.86 3.85 3.83 3.61 3.59 3.58 3.57 3.53 3.50 3.48 3.46 2.70 2.69 2.67 2.42 2.40 2.38 2.36 2.34 2.33 2.11 2.08 2.07 2.06 2.05 2.02 1.91 1.90 1.89 1.88 1.82 1.80 1.79 1.76 1.75 1.73 1.71 1.70 1.69 1.68 1.67 1.65 1.54 1.53 HDO 1.52 1.50 HDO 1.49 1.48 1.43 1.42 1.37 1.32 1.30 1.29 1.26 1.00 0.98 0.97 0.96 0.94
L3-Mes (4)
1.00 1.00 1.01 2.00 1.02 1.05 2.07 1.07 1.00 1.01 6.08 6.00 1.00 1.17 1.03 2.04 1.04 2.04 1.02 3.01 1.15 7.09
f1 (ppm)

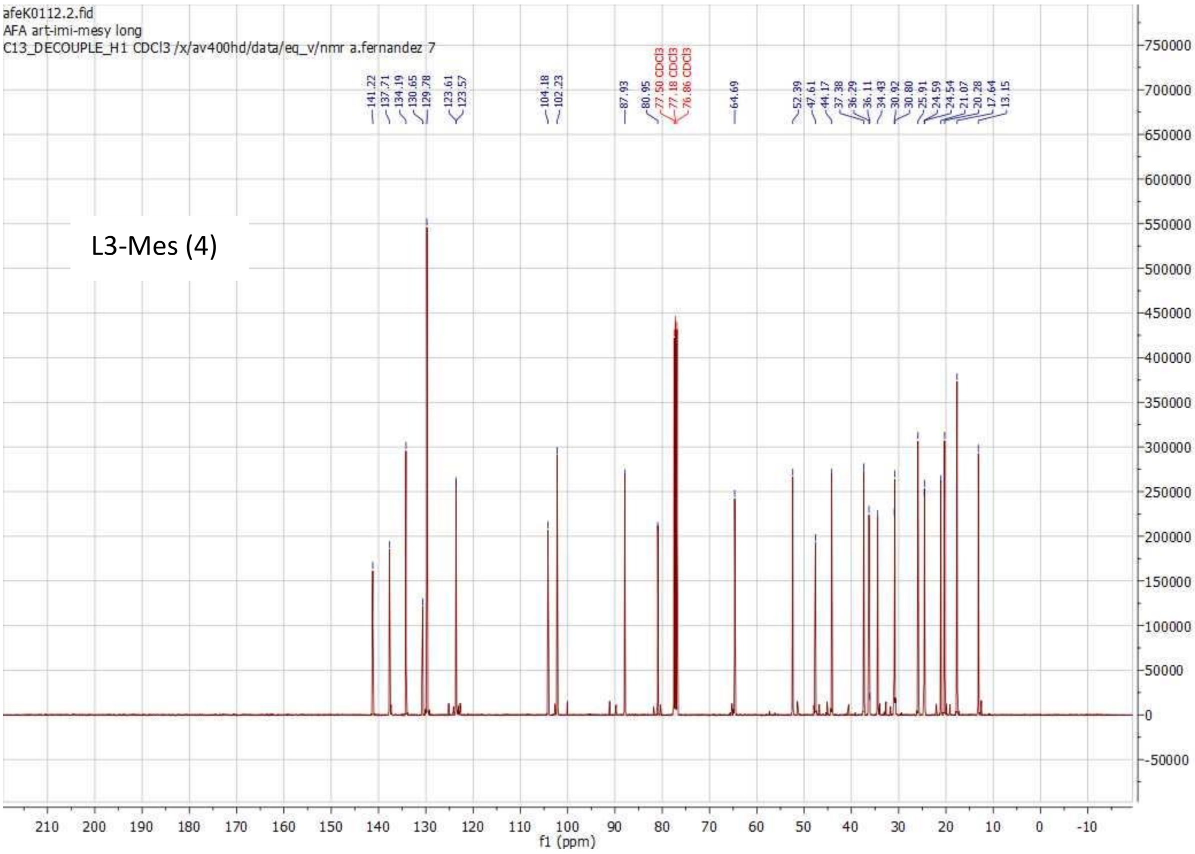
afeK0112.2.fid
AFA art-imi-mesy long
C13_DECOUPLE_H1 CDCl3 /x/av400hd/data/eq_v/nmr a.fernandez 7
141.22 137.71 134.19 130.65 129.78 123.61 123.57 104.18 102.23 87.93 80.95 77.50 CDCl3 77.18 CDCl3 76.86 CDCl3 64.69 52.39 47.61 44.17 37.38 36.29 36.11 34.43 30.92 30.80 25.91 24.59 24.54 21.07 20.28 17.64 13.15
L3-Mes (4)
f1 (ppm)

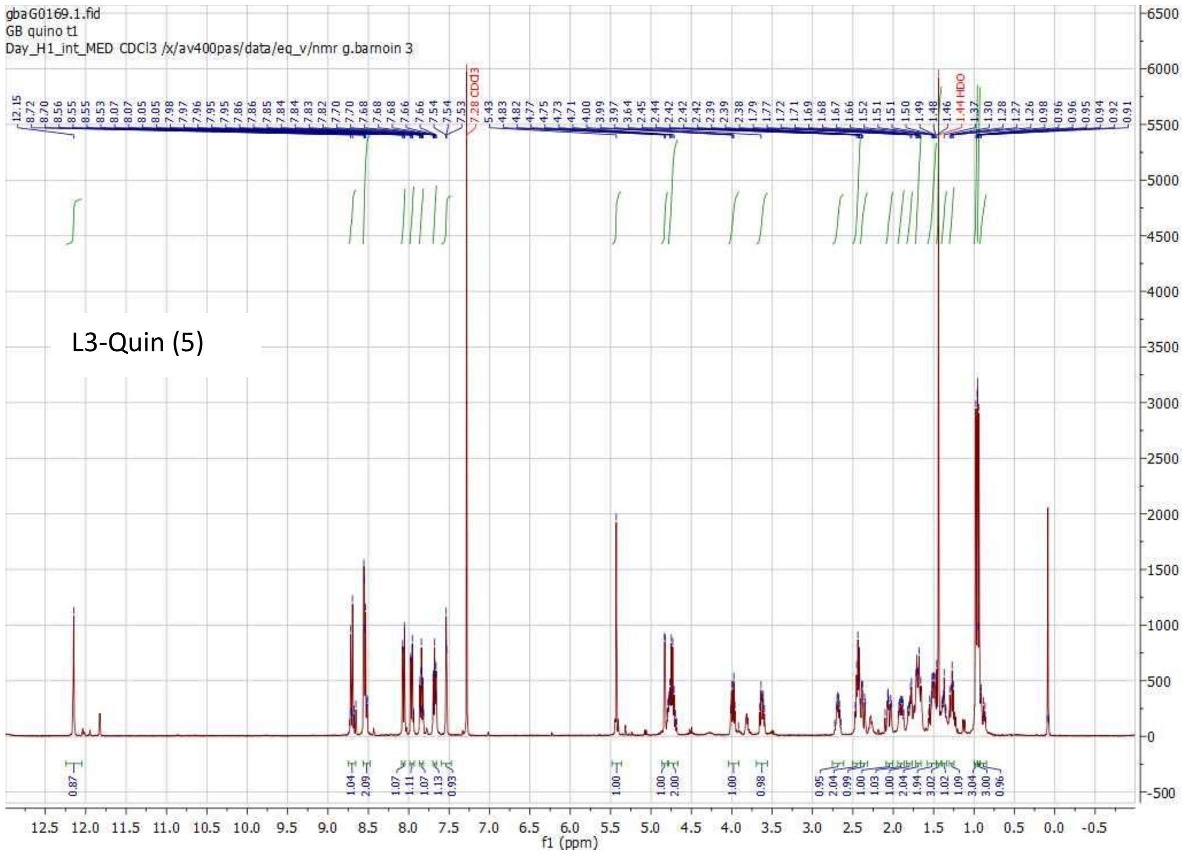
gbaG0169.1.fid
GB quino t1
Day_H1_int_MED CDCl3 /x/av400pas/data/eq_v/nmr g.barnoin 3
L3-Quin (5)
f1 (ppm)

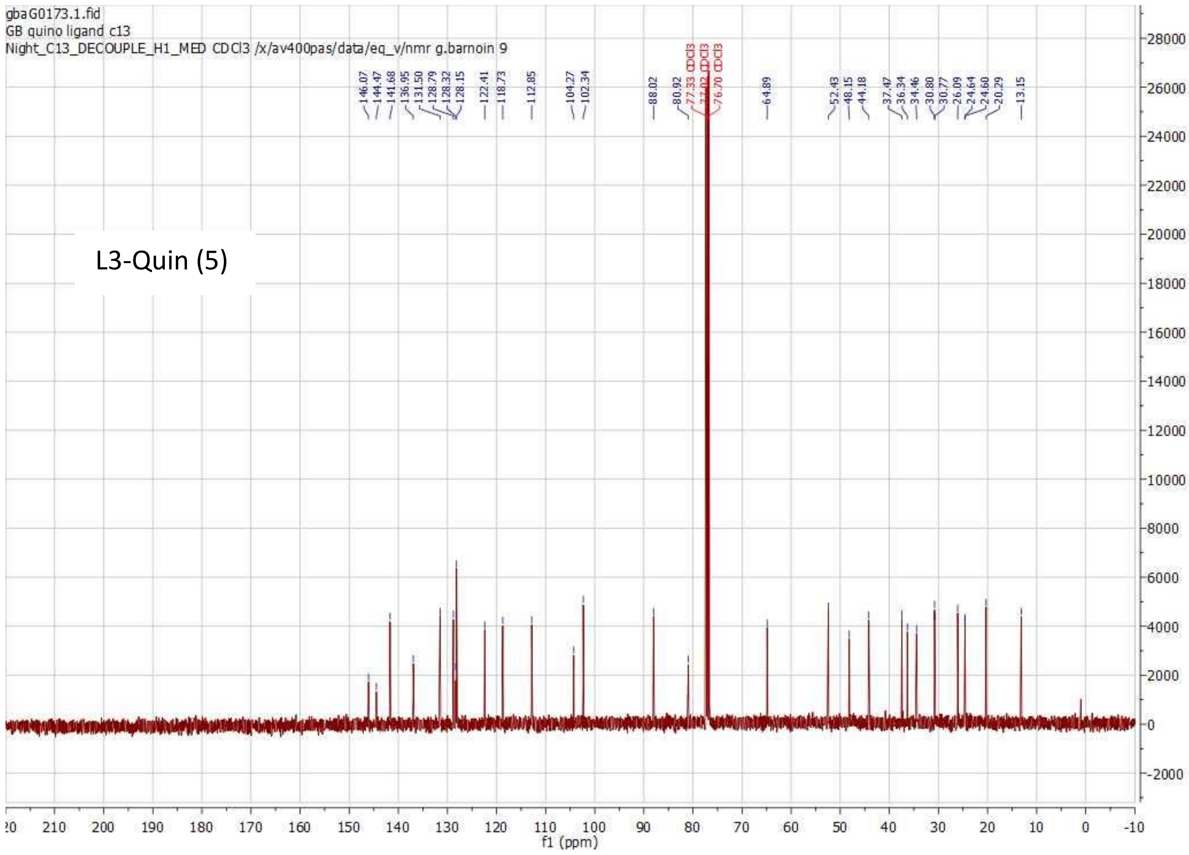
gbaG0173.1.fid
GB quino ligand c13
Night_C13_DECOUPLE_H1_MED CDCl3 /x/av400pas/data/eq_v/nmr g.barnoin 9
L3-Quin (5)
f1 (ppm)

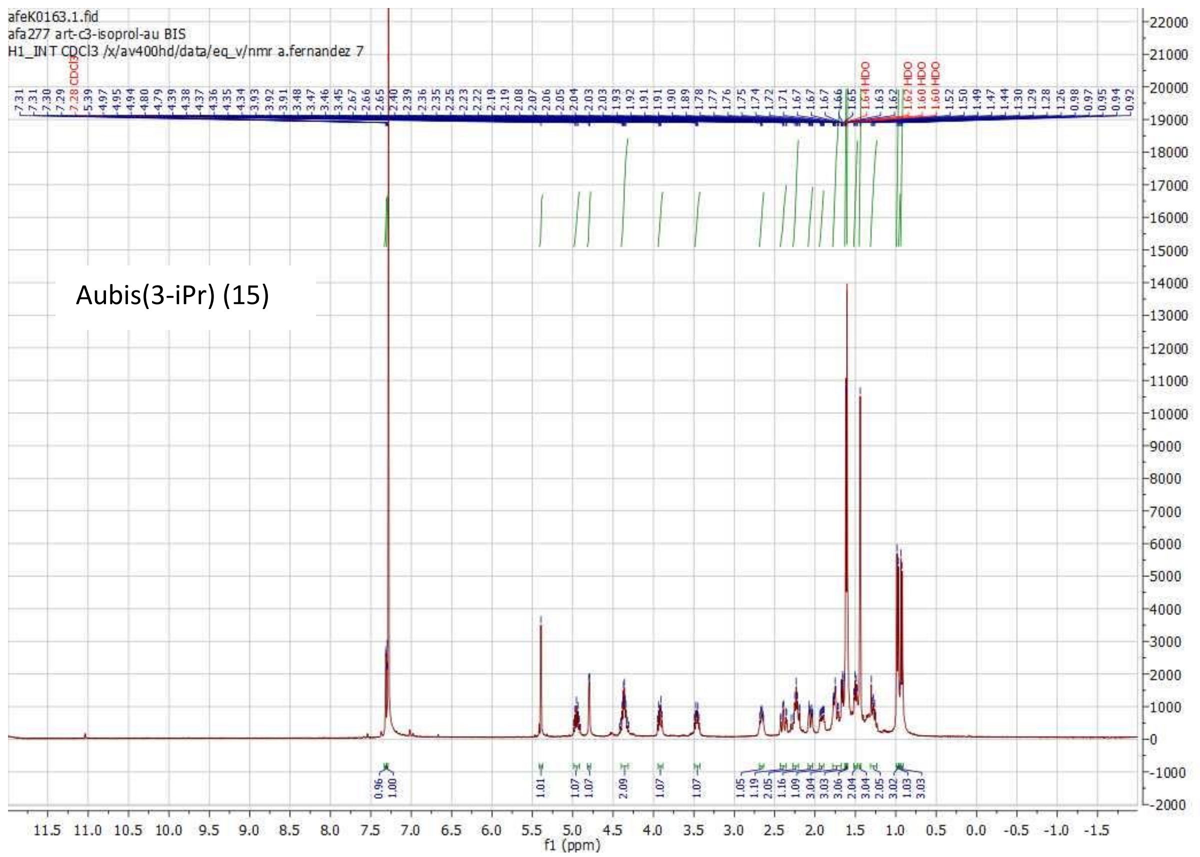
afeK0163.1.fid
afa277 art-c3-isoprol-au BIS
H1_INT CDCl3 /x/av400hd/data/eq_v/nmr a.fernandez 7
Aubis(3-iPr) (15)
f1 (ppm)

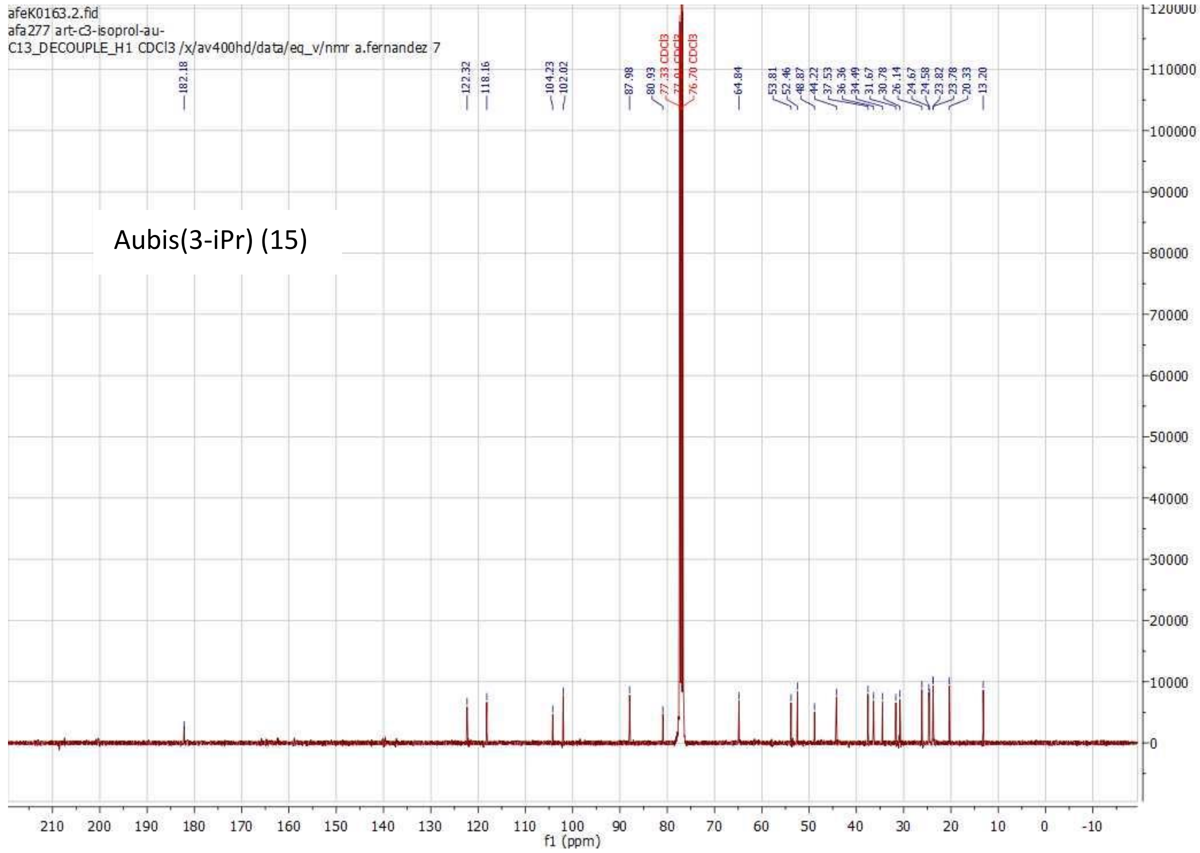
afeK0163.2.fid
afa277 art-c3-isoprol-au-
C13_DECOUPLE_H1 CDCl3 /x/av400hd/data/eq_v/nmr a.fernandez 7
182.18
122.32
118.16
104.23
102.02
87.98
80.93
77.33 CDCl3
77.01 CDCl3
76.70 CDCl3
64.84
53.81
52.46
48.87
44.22
37.53
36.36
34.49
31.67
30.78
26.14
24.67
24.58
23.82
23.78
20.33
13.20
Aubis(3-iPr) (15)
f1 (ppm)

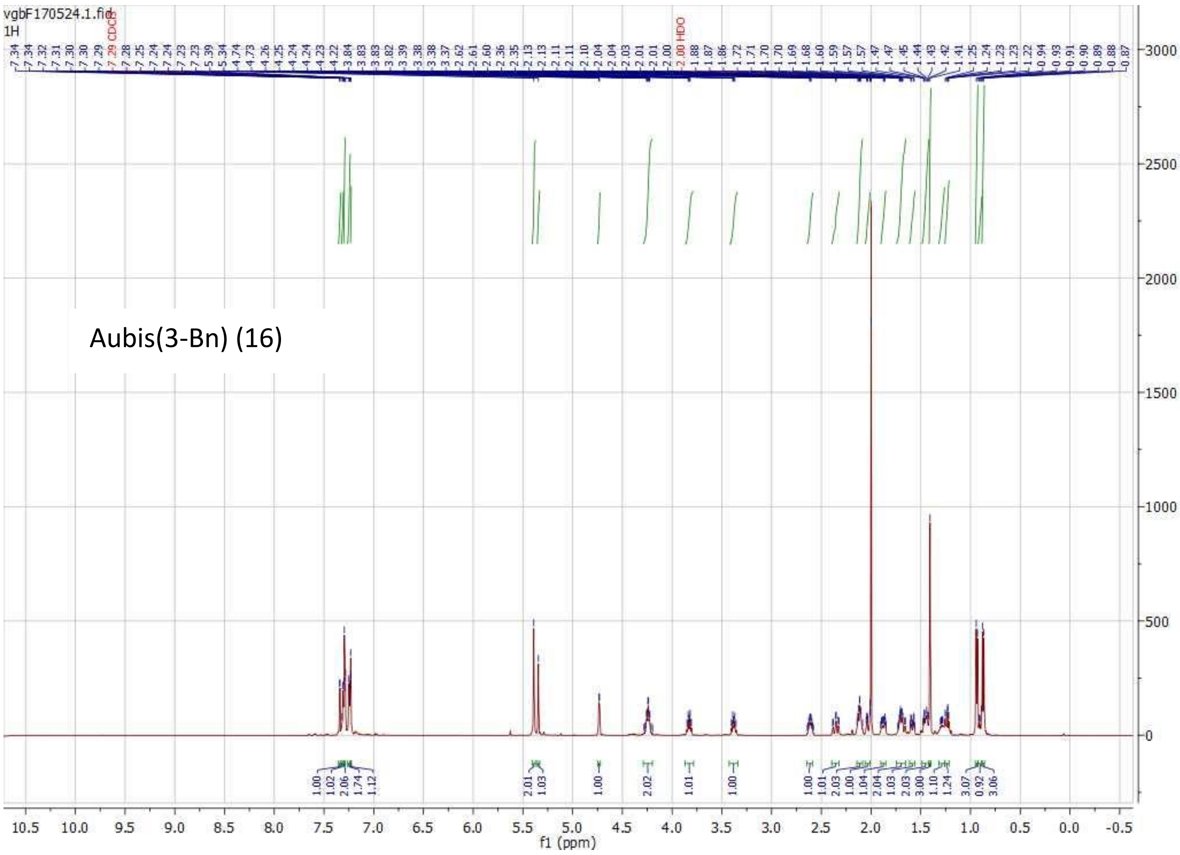
vgbF170524.1.fid
1H
Aubis(3-Bn) (16)
f1 (ppm)

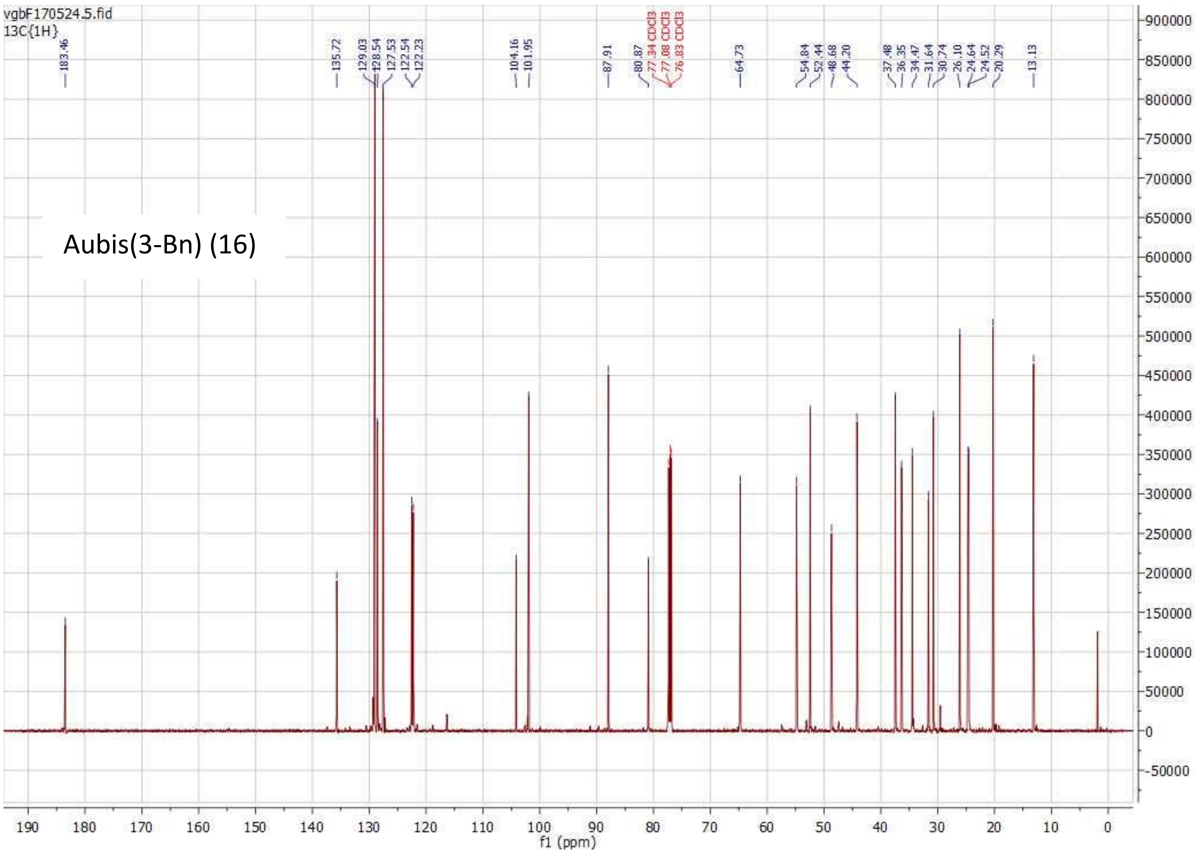
vgbF170524.5.fid
13C{1H}
Aubis(3-Bn) (16)
f1 (ppm)

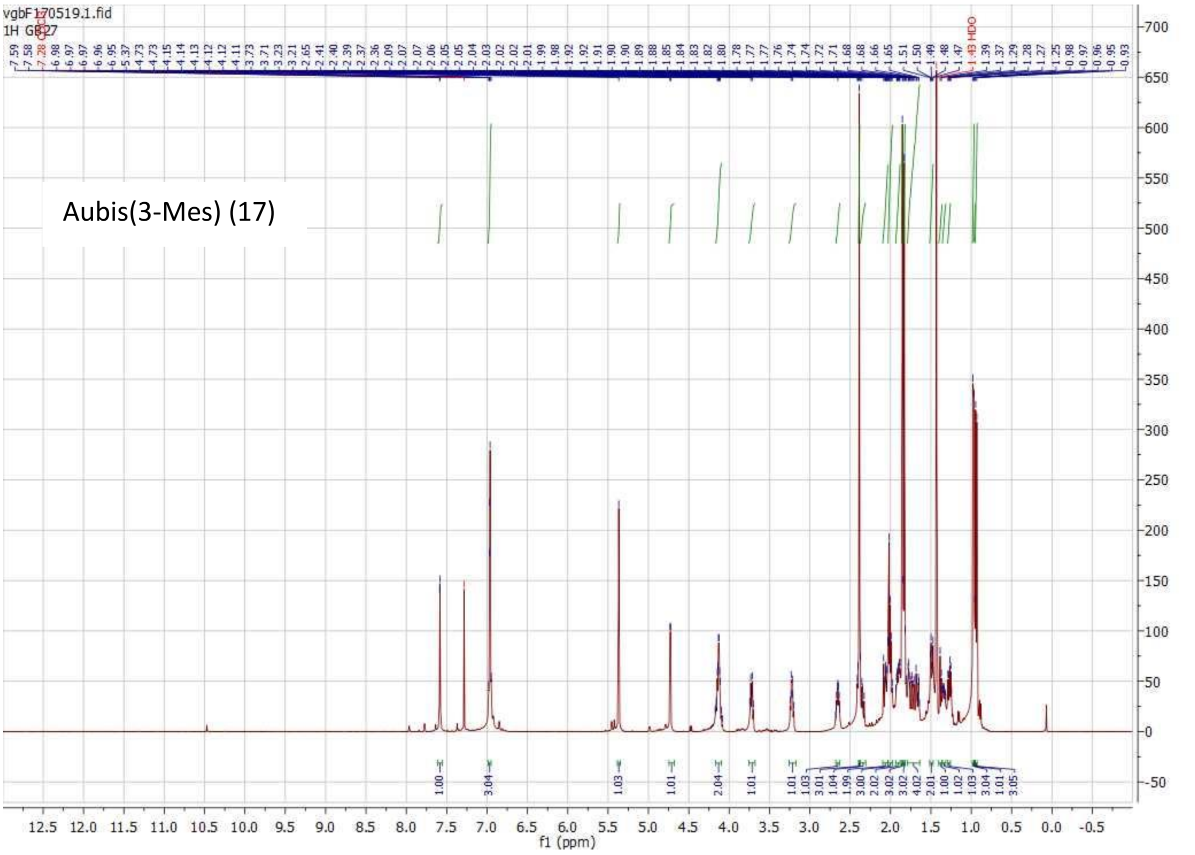
vgbF170519.1.fid
1H GB27
Aubis(3-Mes) (17)
f1 (ppm)

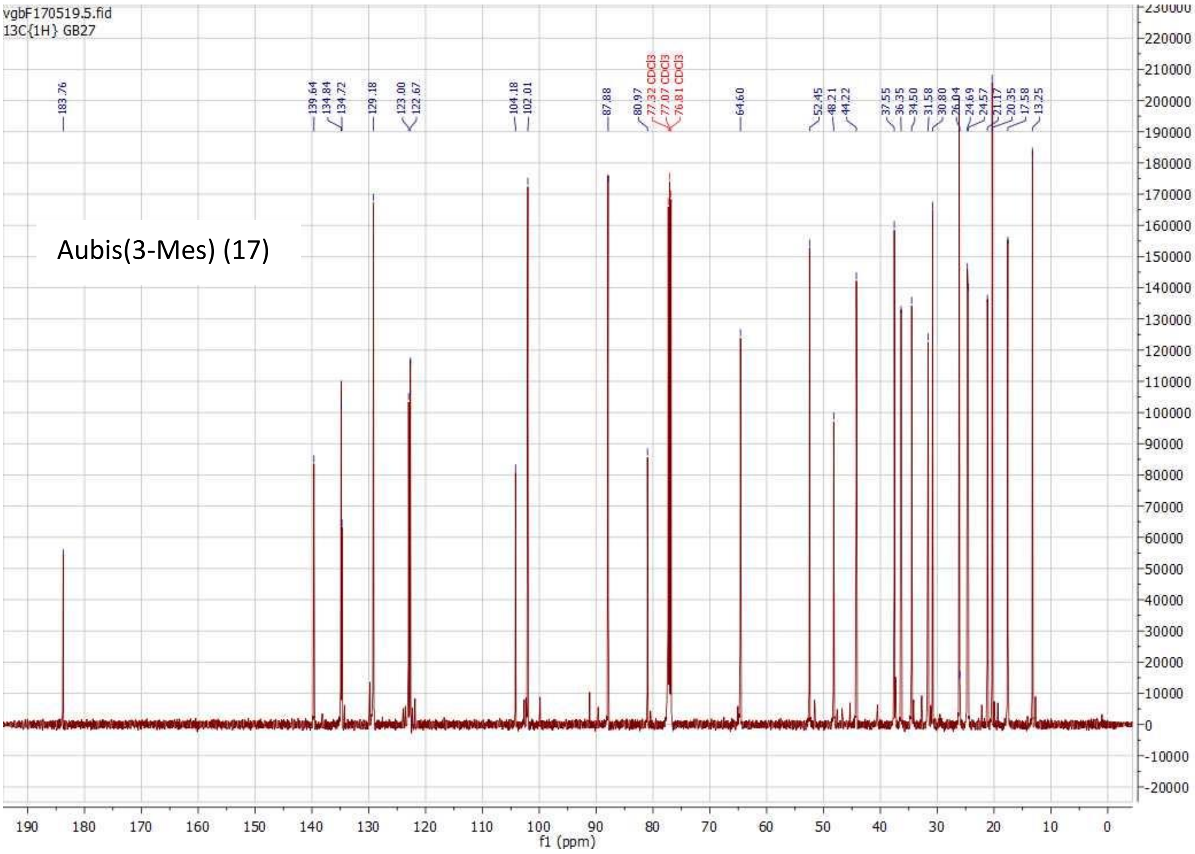
vgbF170519.5.fid
13C{1H} GB27
183.76
139.64
134.84
134.72
129.18
123.00
122.67
104.18
102.01
87.88
80.97
77.32 CDCl3
77.07 CDCl3
76.81 CDCl3
64.60
52.45
48.21
44.22
37.55
36.35
34.50
31.58
30.80
26.04
24.69
24.57
21.17
20.35
17.58
13.25
Aubis(3-Mes) (17)
f1 (ppm)

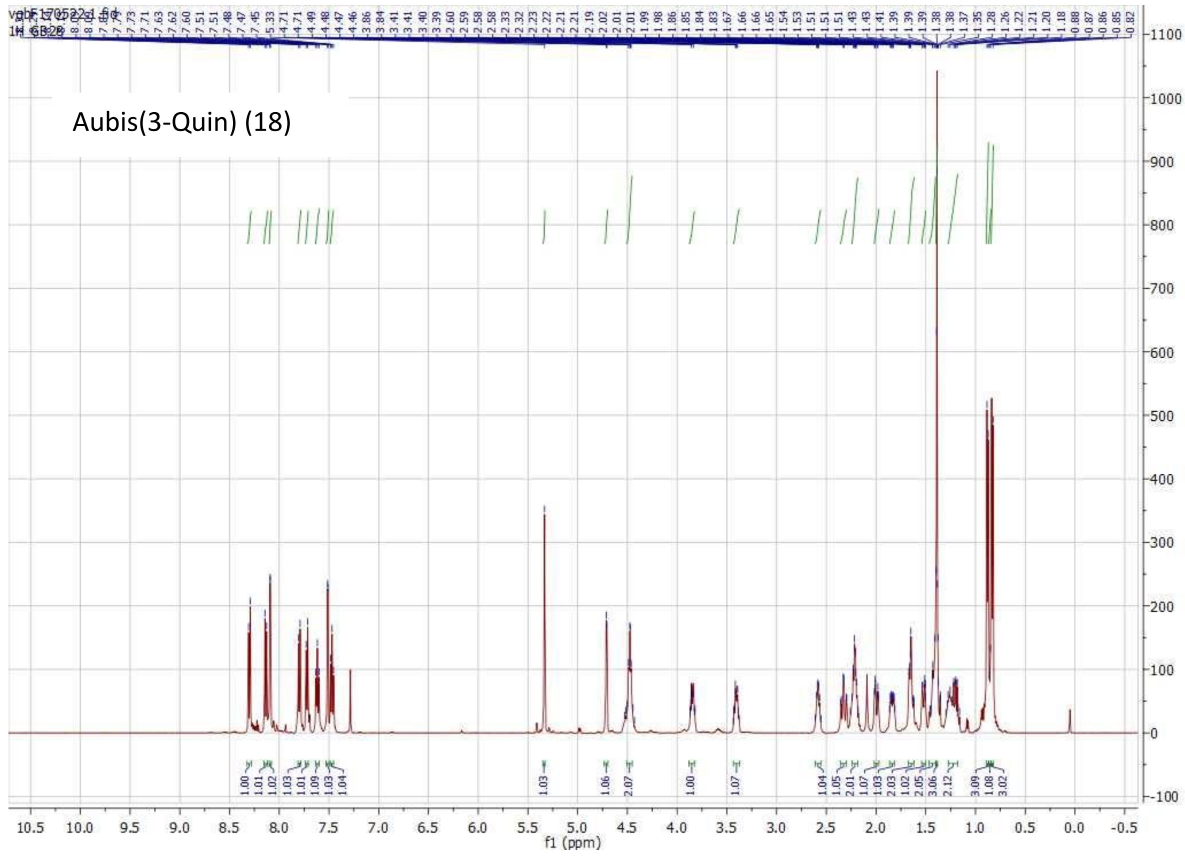
Aubis(3-Quin) (18)
f1 (ppm)

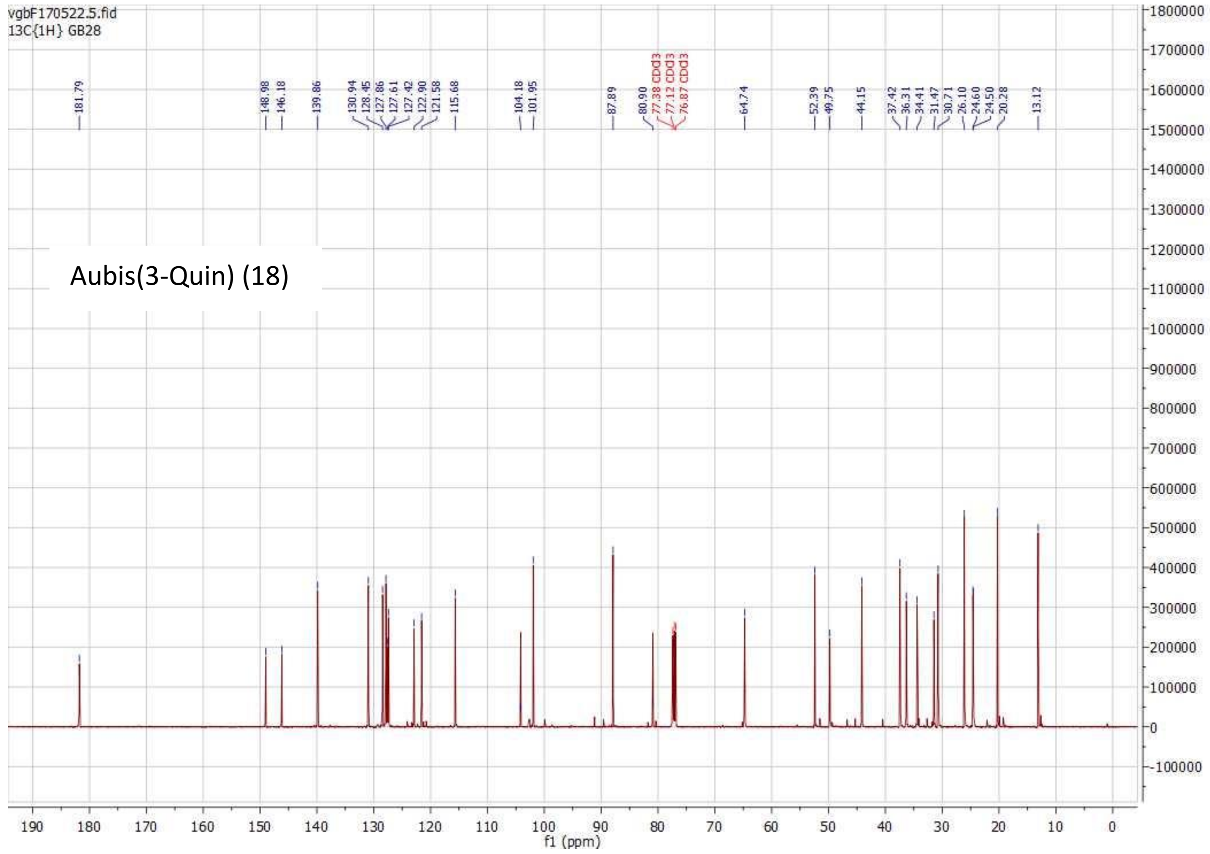
vgbF170522.5.fid
13C{1H} GB28
181.79
148.98
146.18
139.86
130.94
128.45
127.86
127.61
127.42
122.90
121.58
115.68
104.18
101.95
87.89
80.90
77.38 CDCl3
77.12 CDCl3
76.87 CDCl3
64.74
52.39
49.75
44.15
37.42
36.31
34.41
31.47
30.71
26.10
24.60
24.50
20.28
13.12
Aubis(3-Quin) (18)
f1 (ppm)

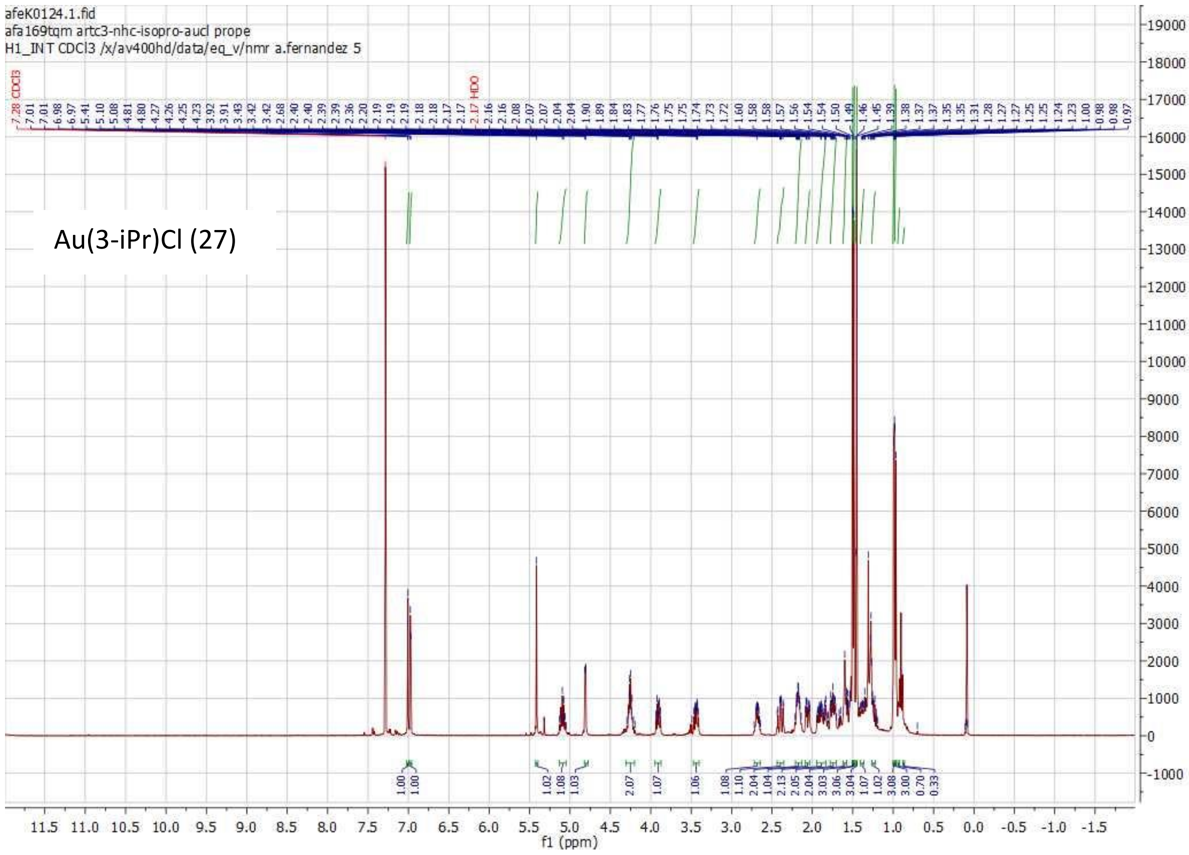
afeK0124.1.fid
afa169tqm artc3-nhc-isopro-aucl prope
H1_INT CDCl3 /x/av400hd/data/eq_v/nmr a.fernandez 5
Au(3-iPr)Cl (27)
f1 (ppm)

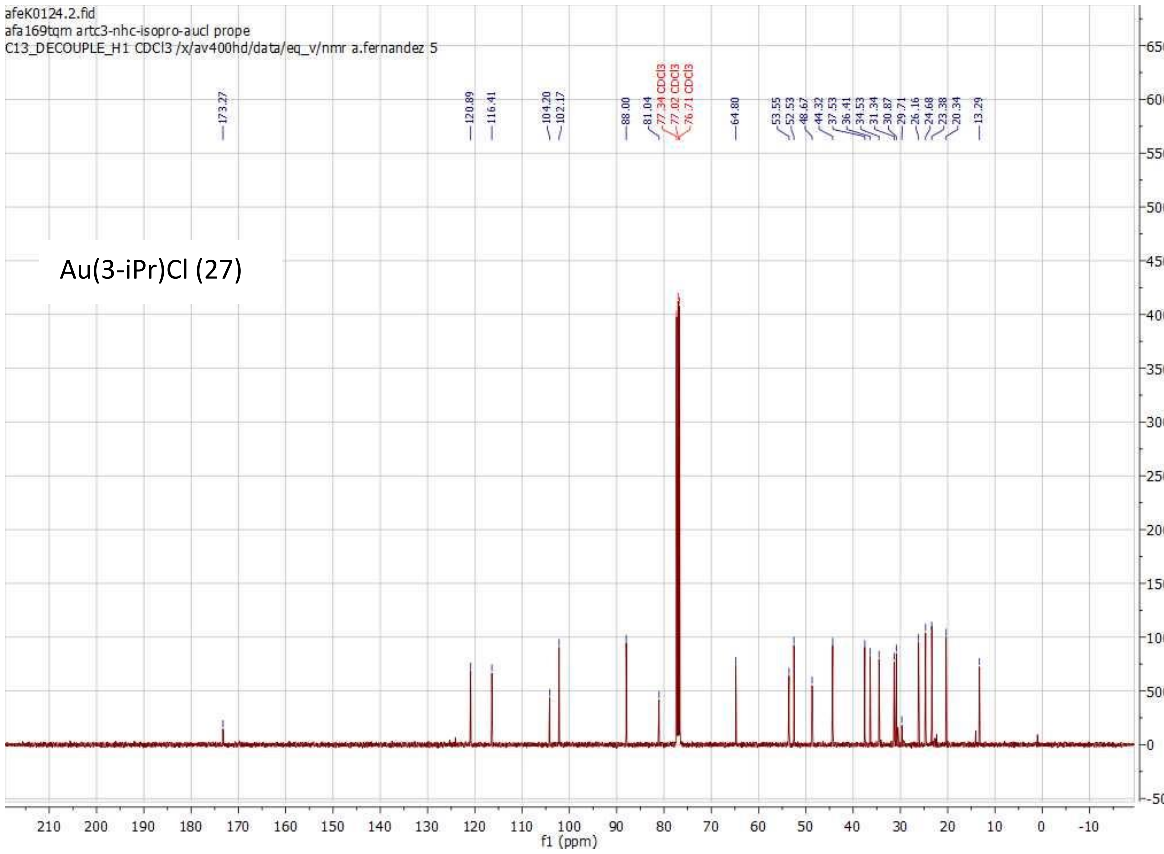
afeK0124.2.fid
afa169tqm artc3-nhc-isopro-aucl prope
C13_DECOUPLE_H1 CDCl3 /x/av400hd/data/eq_v/nmr a.fernandez 5
Au(3-iPr)Cl (27)
f1 (ppm)

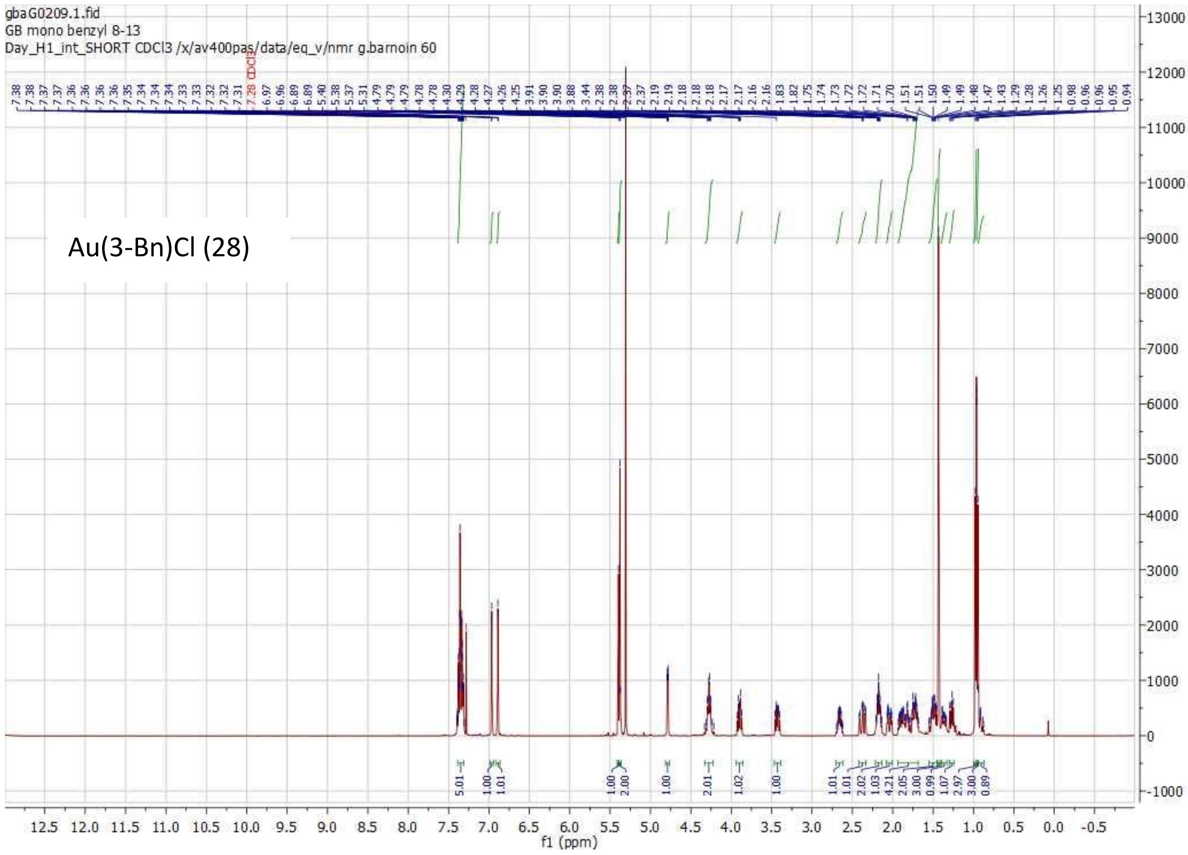
gbaG0209.1.fid
GB mono benzyl 8-13
Day_H1_int_SHORT CDCl3 /x/av400pas/data/eq_v/nmr g.barnoin 60
Au(3-Bn)Cl (28)
f1 (ppm)

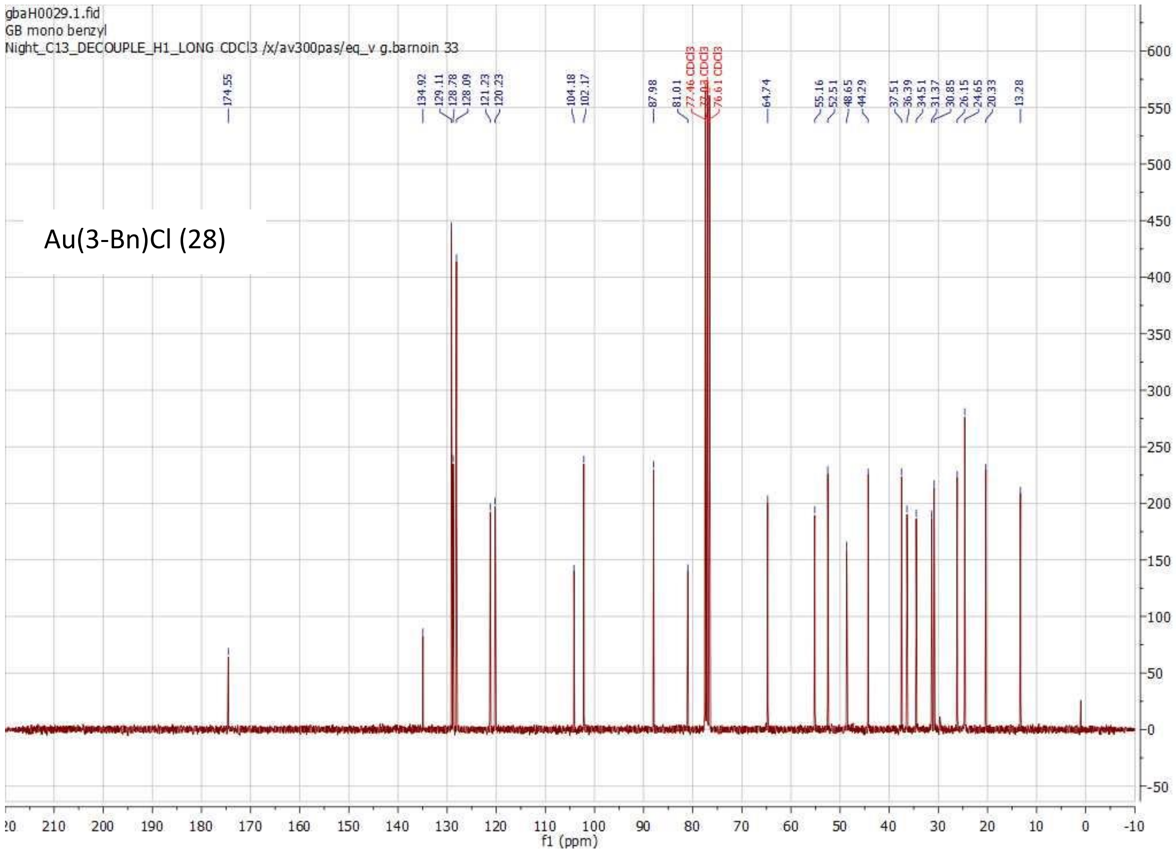
gbaH0029.1.fid
GB mono benzyl
Night_C13_DECOUPLE_H1_LONG CDCl3 /x/av300pas/eq_v g.barnoin 33
174.55
134.92
129.11
128.78
128.09
121.23
120.23
104.18
102.17
87.98
81.01
77.46 CDCl3
76.61 CDCl3
64.74
55.16
52.51
48.65
44.29
37.51
36.39
34.51
31.37
30.85
26.15
24.65
20.33
13.28
Au(3-Bn)Cl (28)
f1 (ppm)

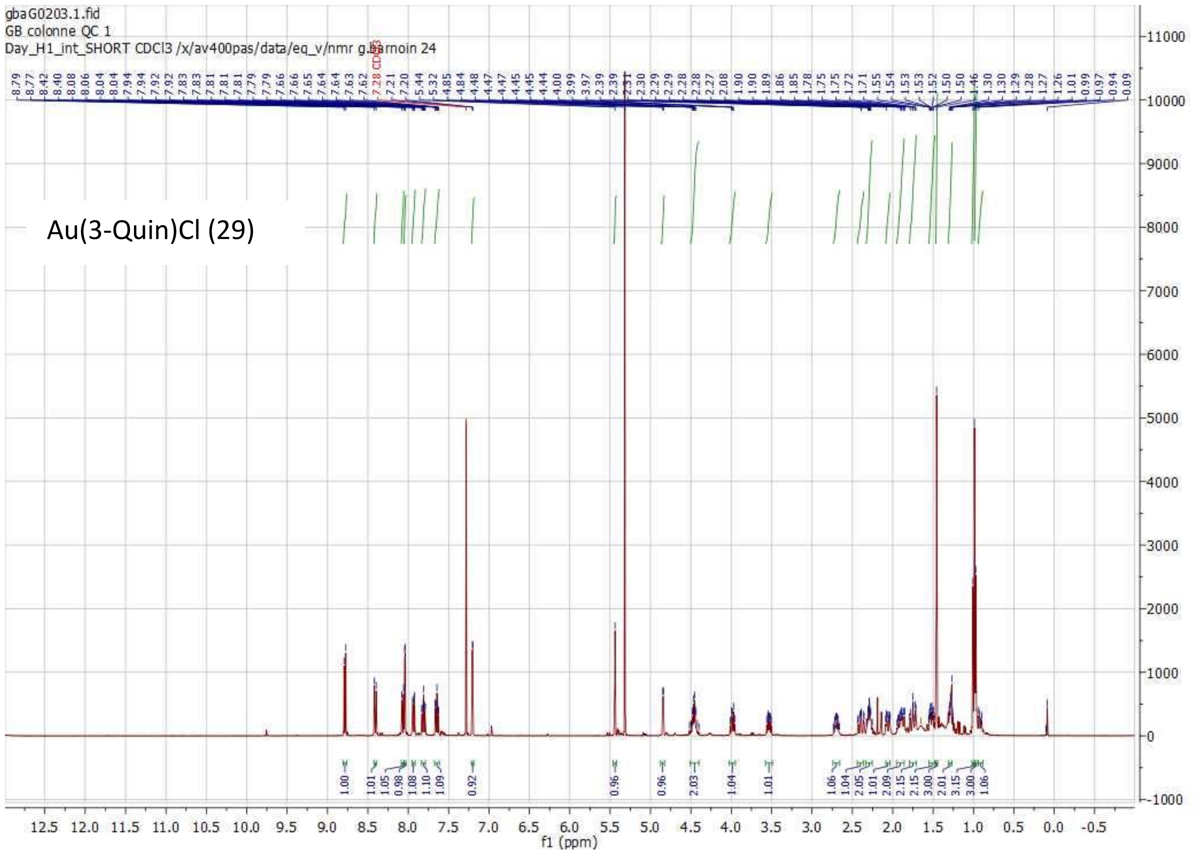
gbaG0203.1.fid
GB colonne QC 1
Day_H1_int_SHORT CDCl3 /x/av400pas/data/eq_v/nmr g.barnoin 24
Au(3-Quin)Cl (29)
f1 (ppm)

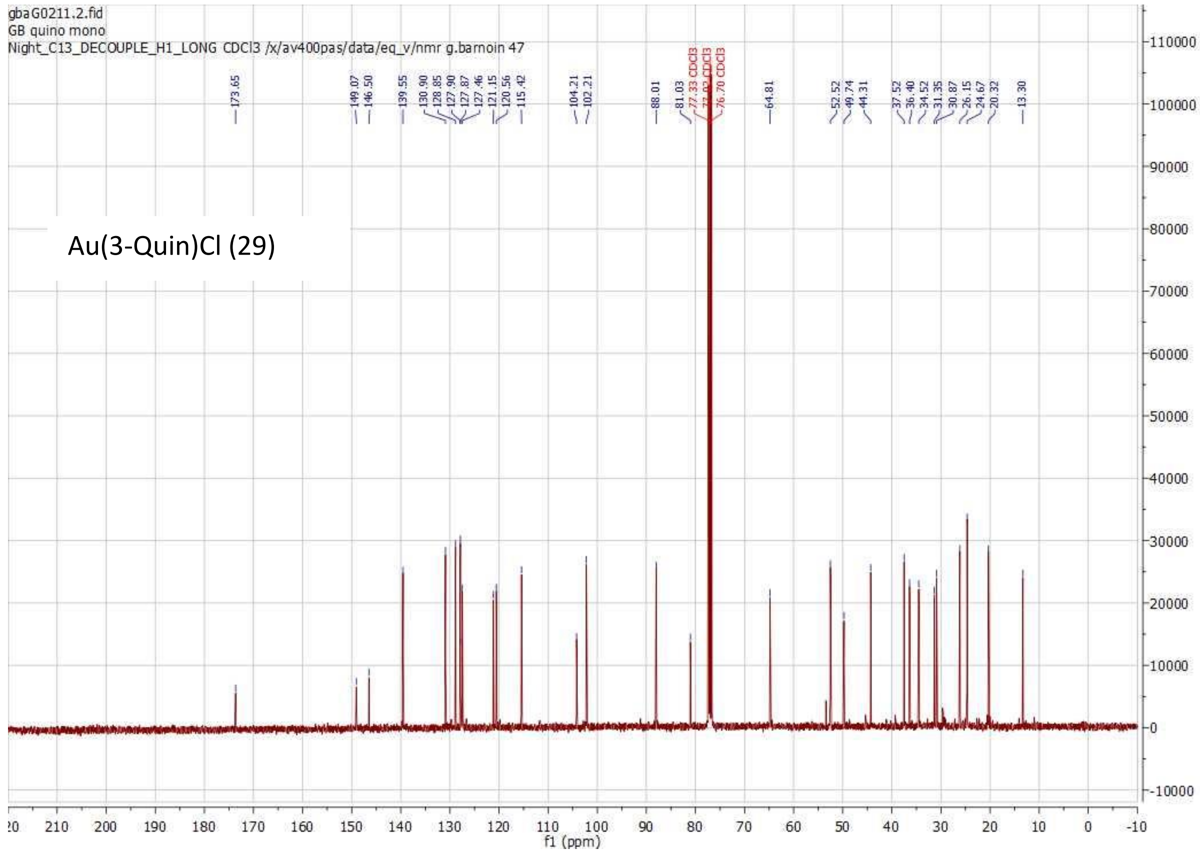
gbaG0211.2.fid
GB quino mono
Night_C13_DECOUPLE_H1_LONG CDCl3 /x/av400pas/data/eq_v/nmr g.barnoin 47
Au(3-Quin)Cl (29)
f1 (ppm)

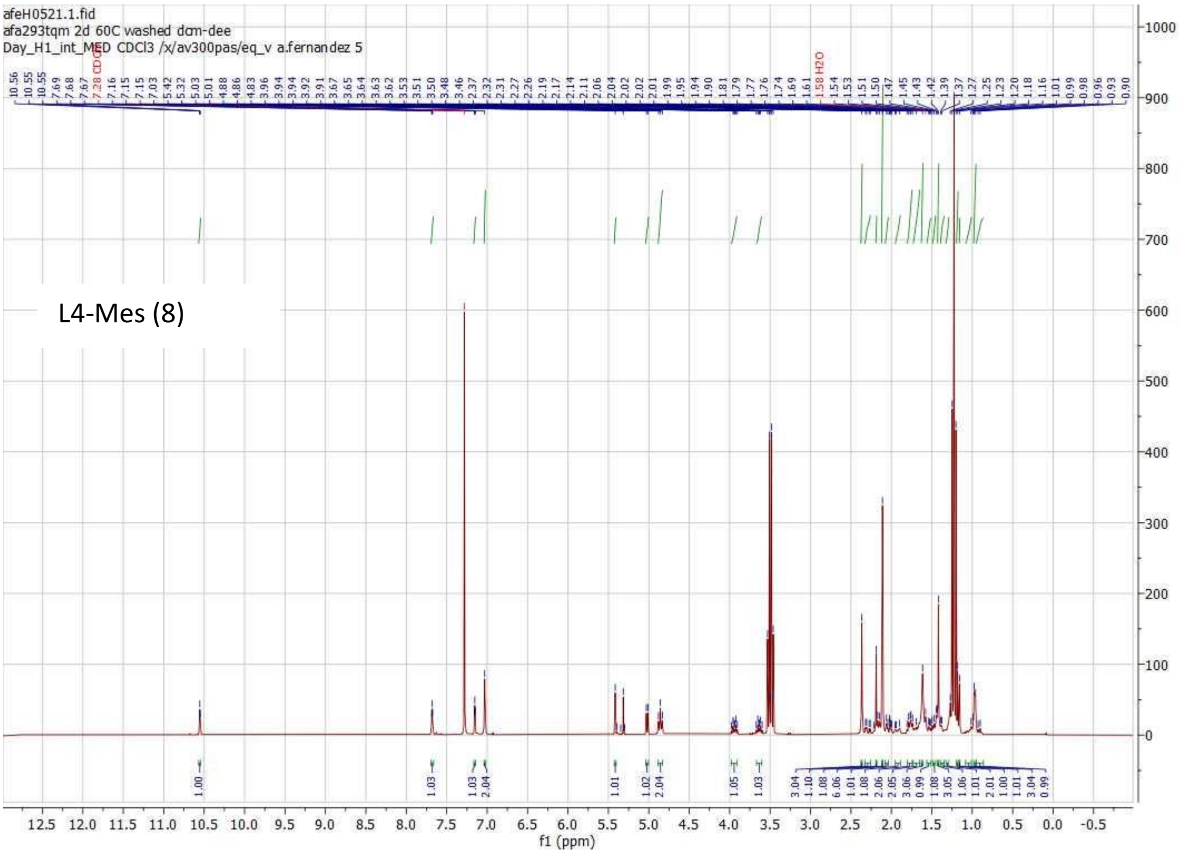
afeH0521.1.fid
afa293tqm 2d 60C washed dcm-dee
Day_H1_int_MED CDCl3 /x/av300pas/eq_v a.fernandez 5
L4-Mes (8)
f1 (ppm)

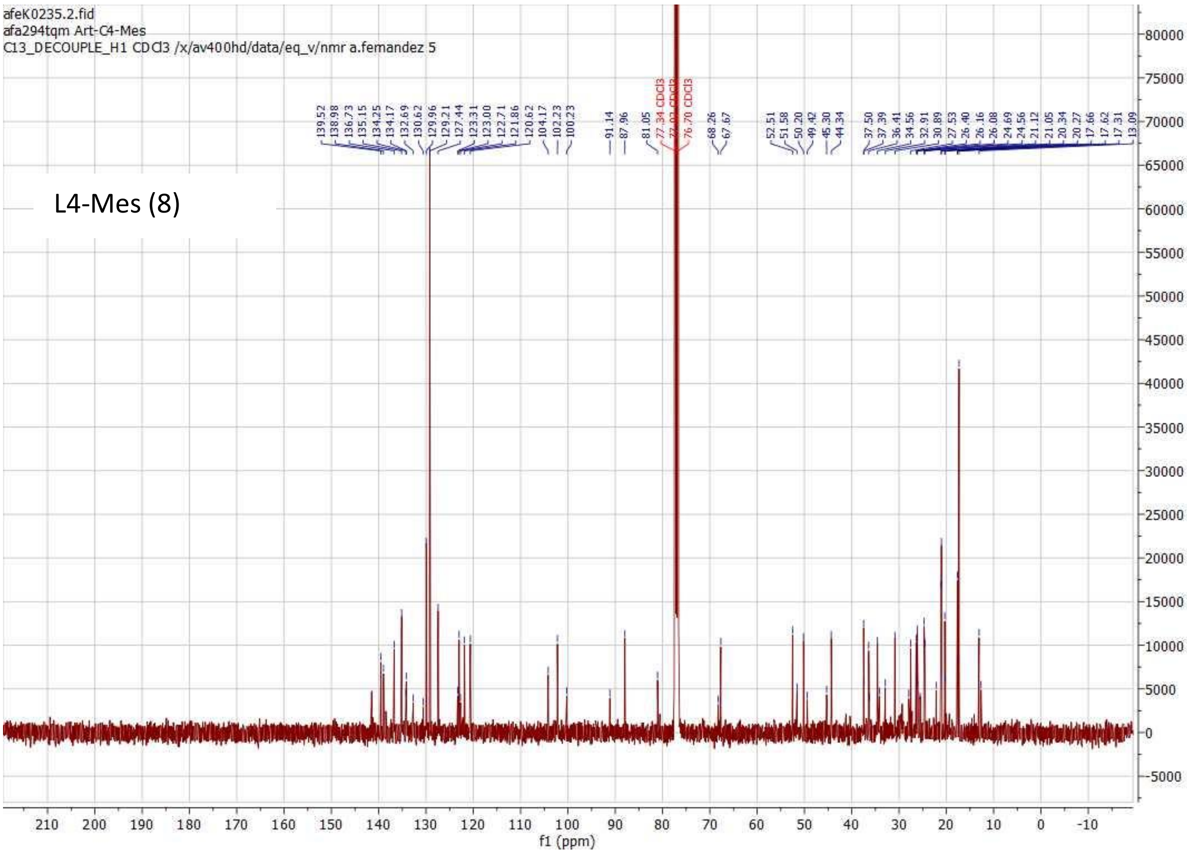
afeK0235.2.fid
afa294tqm Art-C4-Mes
C13_DECOUPLE_H1 CDCl3 /x/av400hd/data/eq_v/nmr a.fernandez 5
L4-Mes (8)
f1 (ppm)

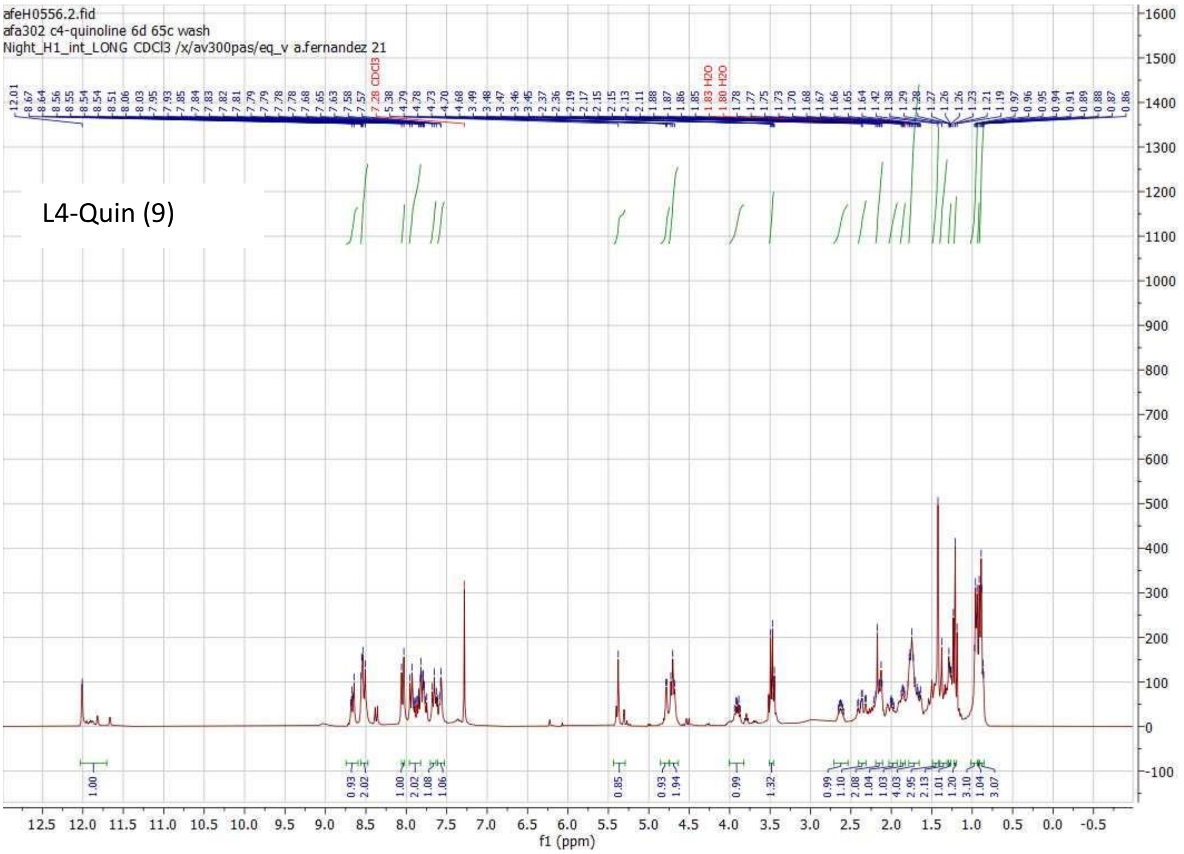
afeH0556.2.fid
afa302 c4-quinoline 6d 65c wash
Night_H1_int_LONG CDCl3 /x/av300pas/eq_v a.fernandez 21
L4-Quin (9)
f1 (ppm)

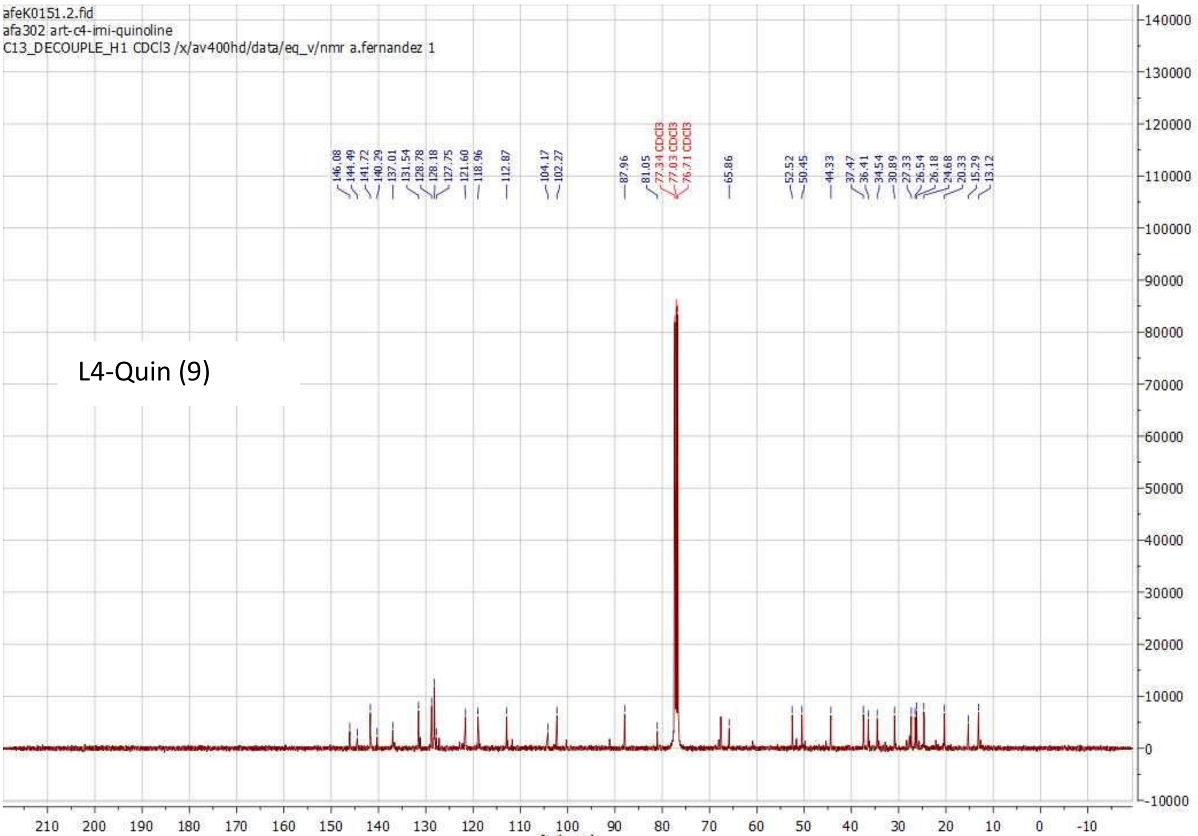
afeK0151.2.fid
afa302 art-c4-imi-quinoline
C13_DECOUPLE_H1 CDCl3 /x/av400hd/data/eq_v/nmr a.fernandez 1
146.08
144.49
141.72
140.29
137.01
131.54
128.78
128.18
127.75
121.60
118.96
112.87
104.17
102.27
87.96
81.05
77.34 CDCl3
77.03 CDCl3
76.71 CDCl3
65.86
52.52
50.45
44.33
37.47
36.41
34.54
30.89
27.33
26.54
26.18
24.68
20.33
15.29
13.12
L4-Quin (9)
f1 (ppm)

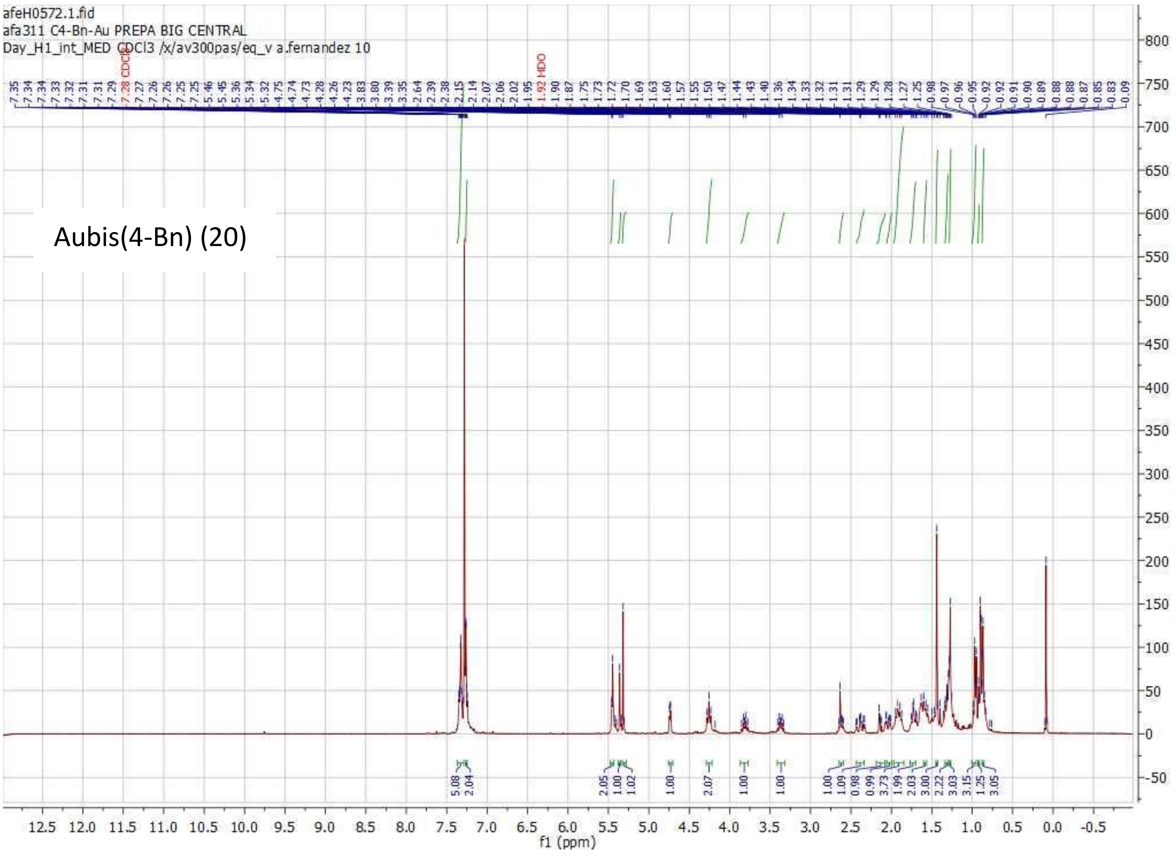
afeH0572.1.fid
afa311 C4-Bn-Au PREPA BIG CENTRAL
Day_H1_int_MED CDCl3 /x/av300pas/eq_v a.fernandez 10
7.35 7.34 7.34 7.33 7.32 7.31 7.31 7.29 7.28 CDCl3 7.27 7.26 7.26 7.25 7.25 5.46 5.45 5.36 5.34 5.32 4.75 4.74 4.73 4.28 4.26 4.23 3.83 3.80 3.39 3.35 2.64 2.39 2.38 2.15 2.14 2.07 2.06 2.02 1.95 1.92 HDO 1.90 1.87 1.75 1.73 1.72 1.70 1.69 1.63 1.60 1.57 1.55 1.50 1.47 1.44 1.43 1.40 1.36 1.34 1.33 1.32 1.31 1.31 1.29 1.29 1.29 1.28 1.27 1.25 0.98 0.97 0.96 0.95 0.92 0.92 0.91 0.90 0.89 0.88 0.88 0.87 0.85 0.83 0.09
Aubis(4-Bn) (20)
5.08 2.04 2.05 1.00 1.02 1.00 2.07 1.00 1.00 1.00 1.09 0.98 0.99 3.73 1.99 2.03 3.00 2.22 3.03 3.15 1.25 3.05
12.5 12.0 11.5 11.0 10.5 10.0 9.5 9.0 8.5 8.0 7.5 7.0 6.5 6.0 5.5 5.0 4.5 4.0 3.5 3.0 2.5 2.0 1.5 1.0 0.5 0.0 -0.5
f1 (ppm)
800 750 700 650 600 550 500 450 400 350 300 250 200 150 100 50 0 -50

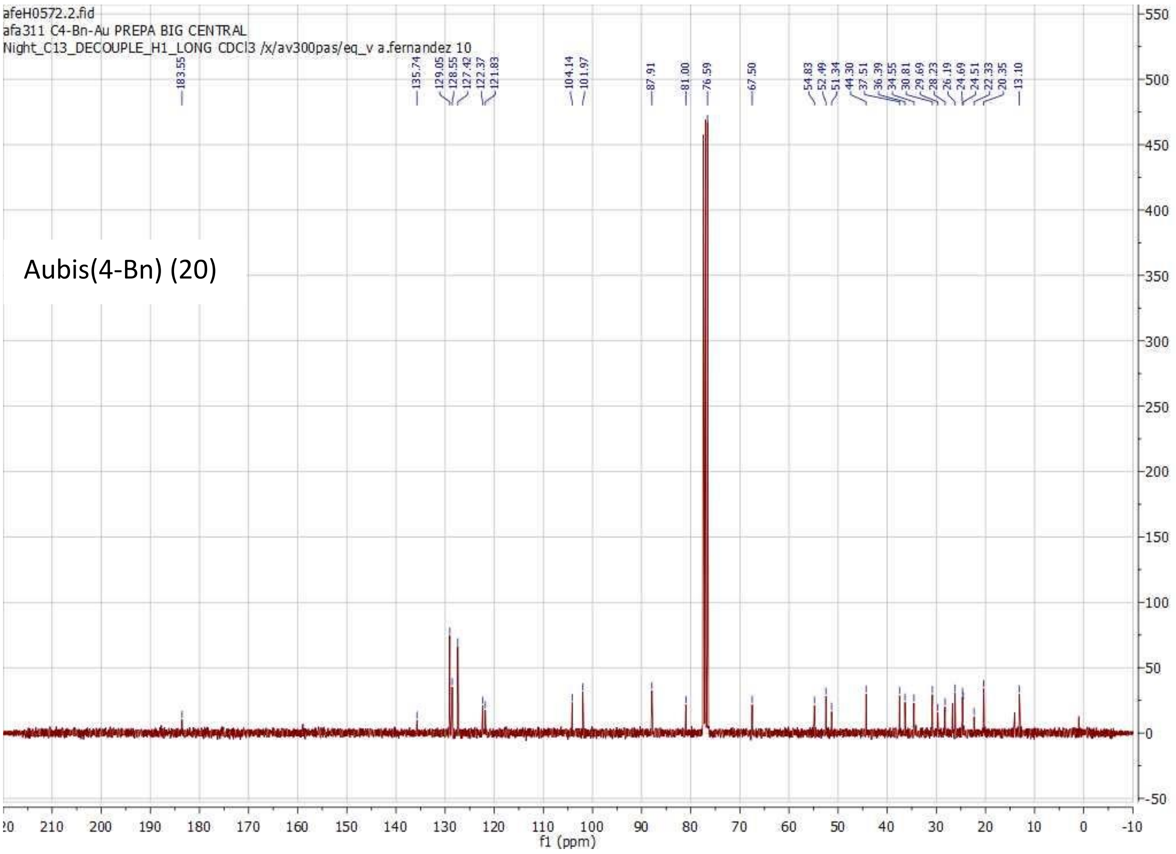
afeH0572.2.fid
afa311 C4-Bn-Au PREPA BIG CENTRAL
Night_C13_DECOUPLE_H1_LONG CDCl3 /x/av300pas/eq_v a.fernandez 10
183.55 135.74 129.05 128.55 127.42 122.37 121.83 104.14 101.97 87.91 81.00 76.59 67.50 54.83 52.49 51.34 44.30 37.51 36.39 34.55 30.81 29.69 28.23 26.19 24.69 24.51 22.33 20.35 13.10
Aubis(4-Bn) (20)
220 210 200 190 180 170 160 150 140 130 120 110 100 90 80 70 60 50 40 30 20 10 0 -10
f1 (ppm)
550 500 450 400 350 300 250 200 150 100 50 0 -50

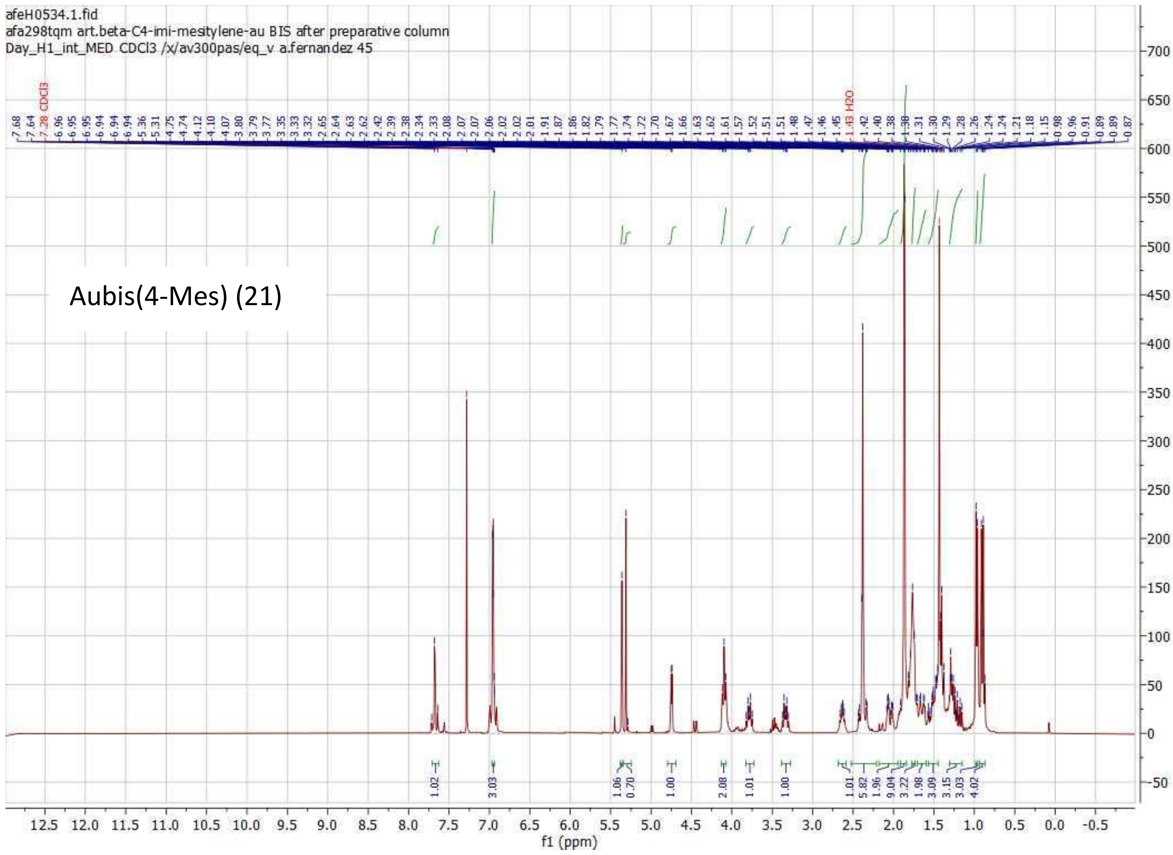
afeH0534.1.fid
afa298tqm art.beta-C4-imi-mesitylene-au BIS after preparative column
Day_H1_int_MED CDCl3 /x/av300pas/eq_v a.fernandez 45
Aubis(4-Mes) (21)
f1 (ppm)

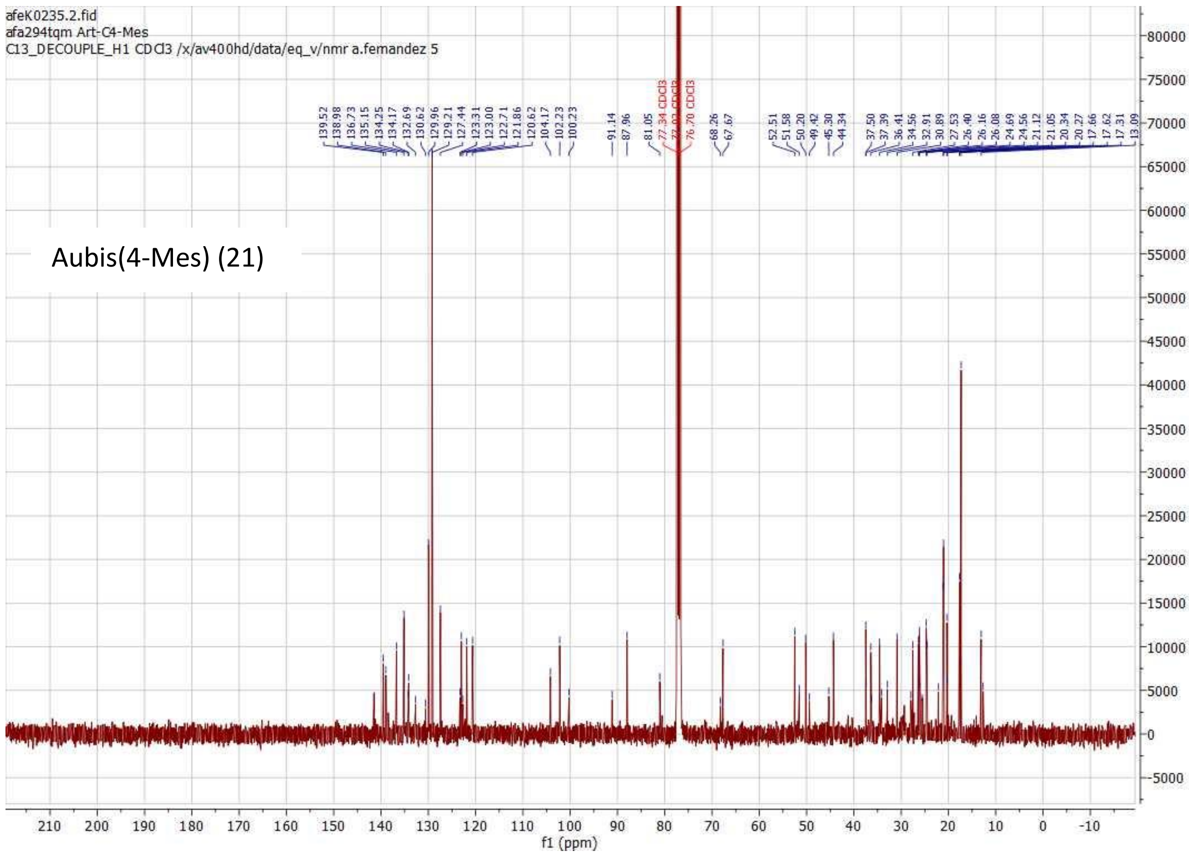
afeK0235.2.fid
afa294tqm Art-C4-Mes
C13_DECOUPLE_H1 CDCl3 /x/av400hd/data/eq_v/nmr a.fernandez 5
Aubis(4-Mes) (21)
f1 (ppm)

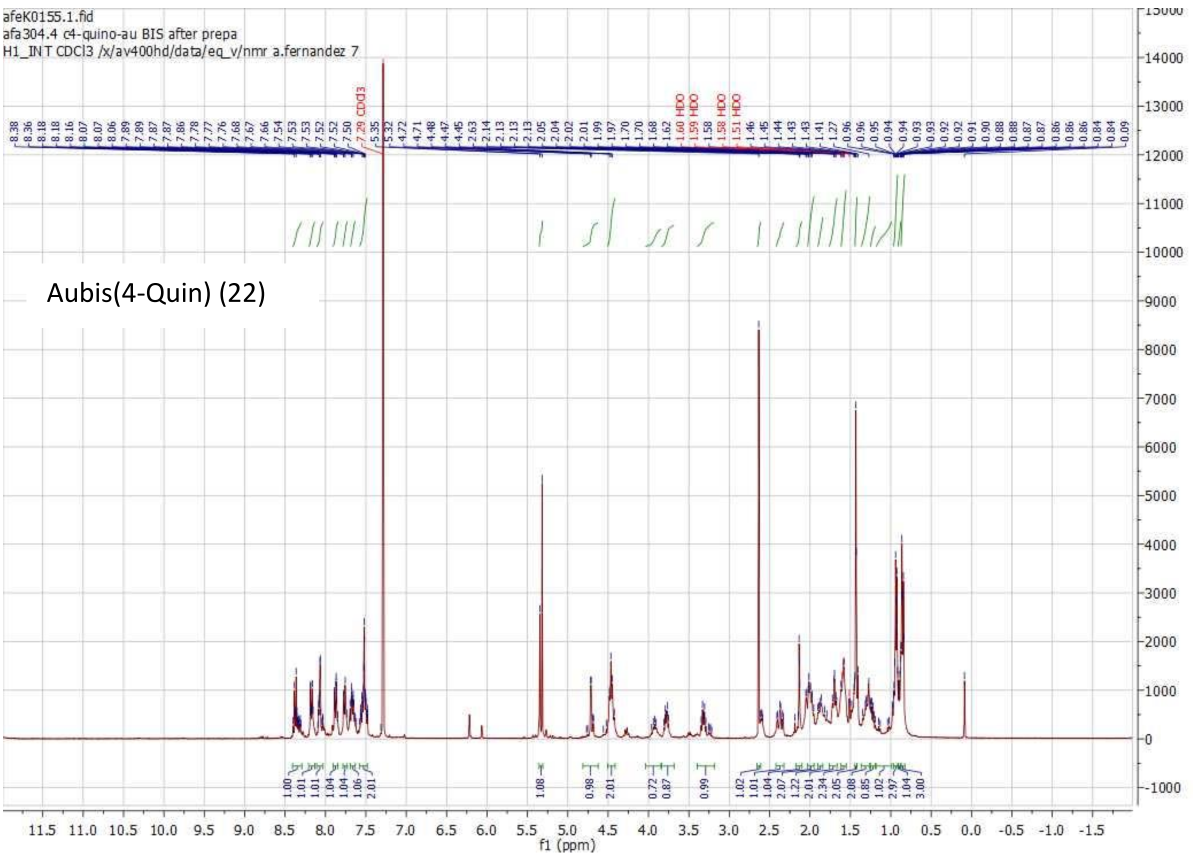
afeK0155.1.fid
afa304.4 c4-quino-au BIS after prepa
H1_INT CDCl3 /x/av400hd/data/eq_v/nmr a.fernandez 7
Aubis(4-Quin) (22)
f1 (ppm)

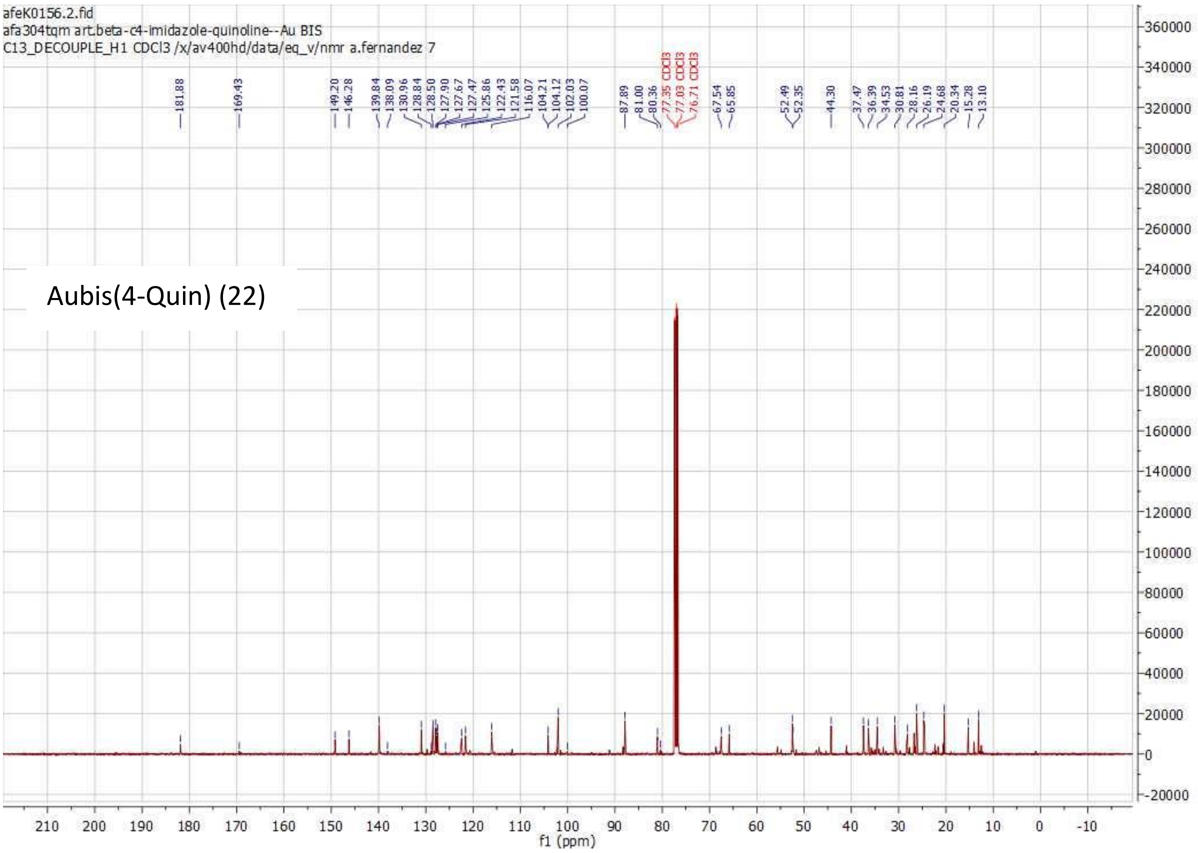
afeK0156.2.fid
afa304tqm art.beta-c4-imidazole-quinoline--Au BIS
C13_DECOUPLE_H1 CDCl3 /x/av400hd/data/eq_v/nmr a.fernandez 7
181.88
169.43
149.20
146.28
139.84
138.09
130.96
128.84
128.50
127.90
127.67
127.47
125.86
122.43
121.58
116.07
104.21
104.12
102.03
100.07
87.89
81.00
80.36
77.35 CDCl3
77.03 CDCl3
76.71 CDCl3
67.54
65.85
52.49
52.35
44.30
37.47
36.39
34.53
30.81
28.16
26.19
24.68
20.34
15.28
13.10
Aubis(4-Quin) (22)
f1 (ppm)

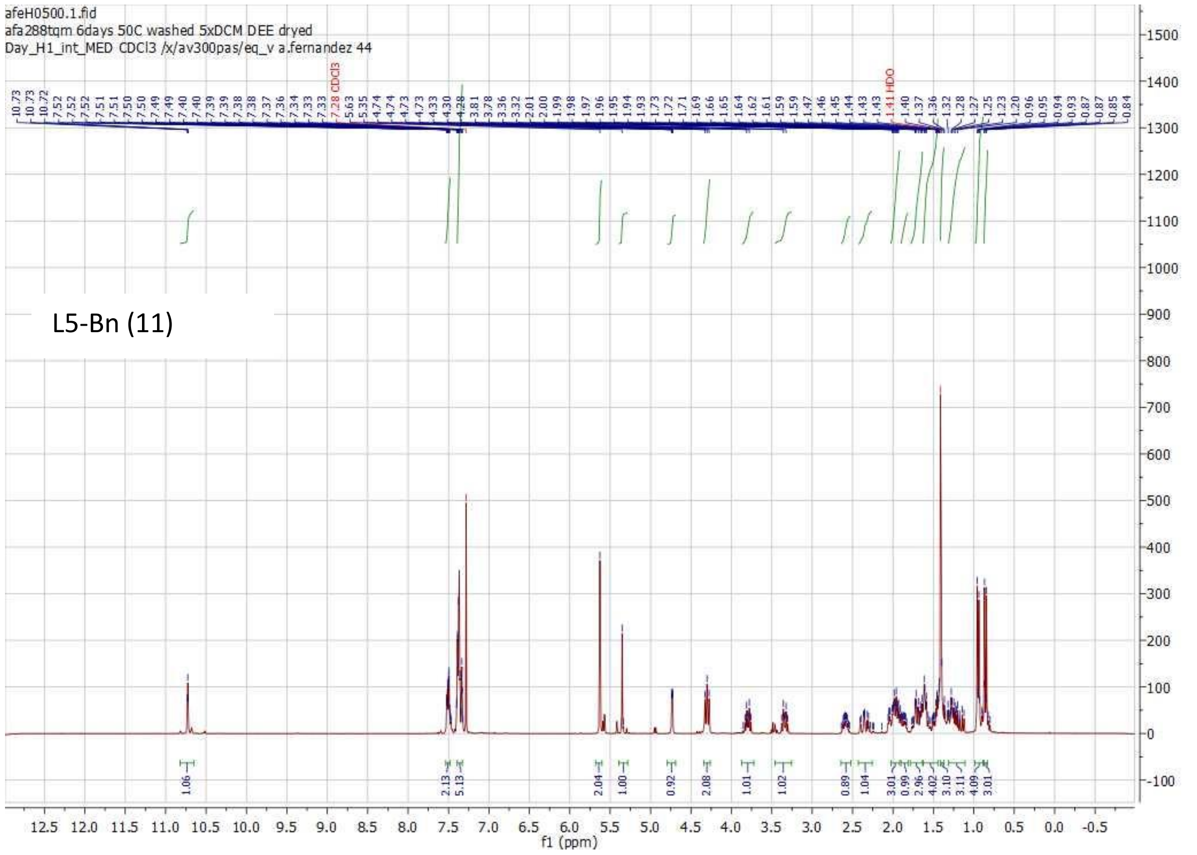
afeH0500.1.fid
afa288tqm 6days 50C washed 5xDCM DEE dryed
Day_H1_int_MED CDCl3 /x/av300pas/eq_v a.fernandez 44
L5-Bn (11)
f1 (ppm)

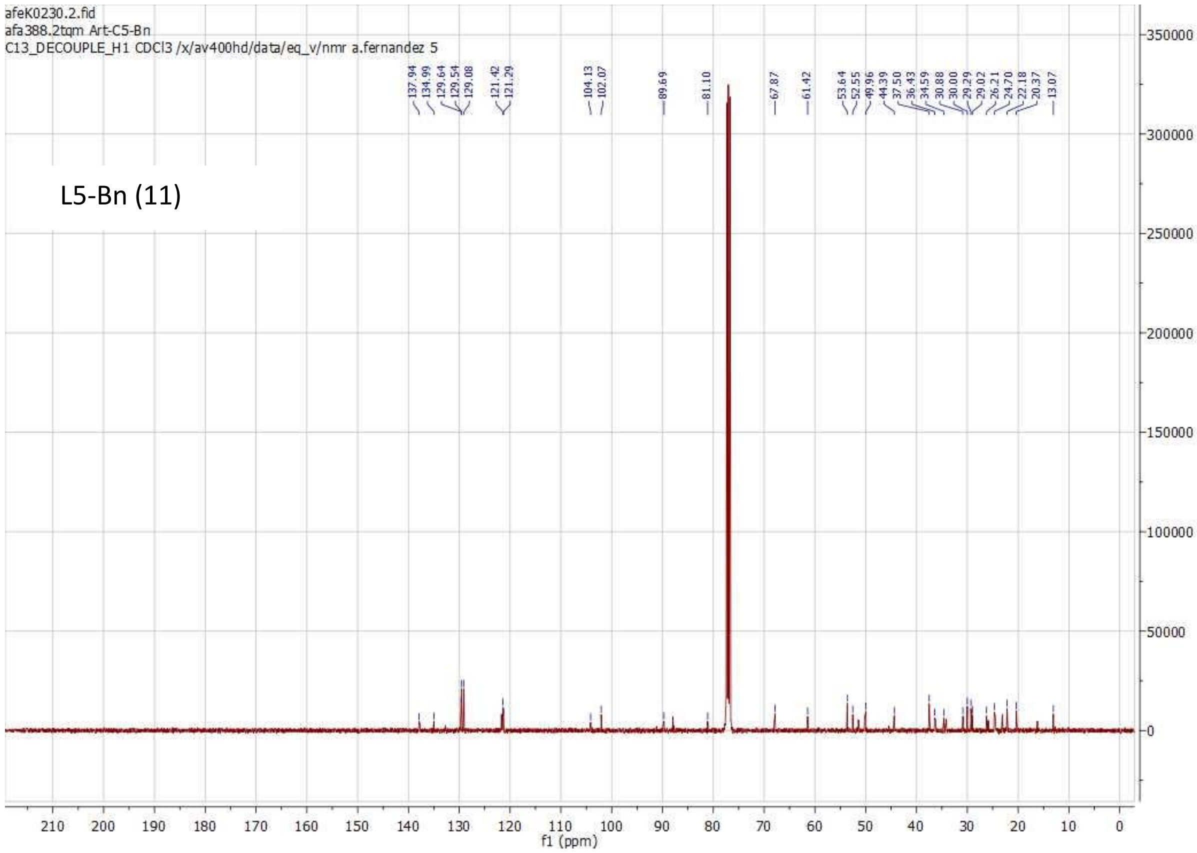
afeK0230.2.fid
afa388.2tqm Art-C5-Bn
C13_DECOUPLE_H1 CDCl3 /x/av400hd/data/eq_v/nmr a.fernandez 5
L5-Bn (11)
f1 (ppm)

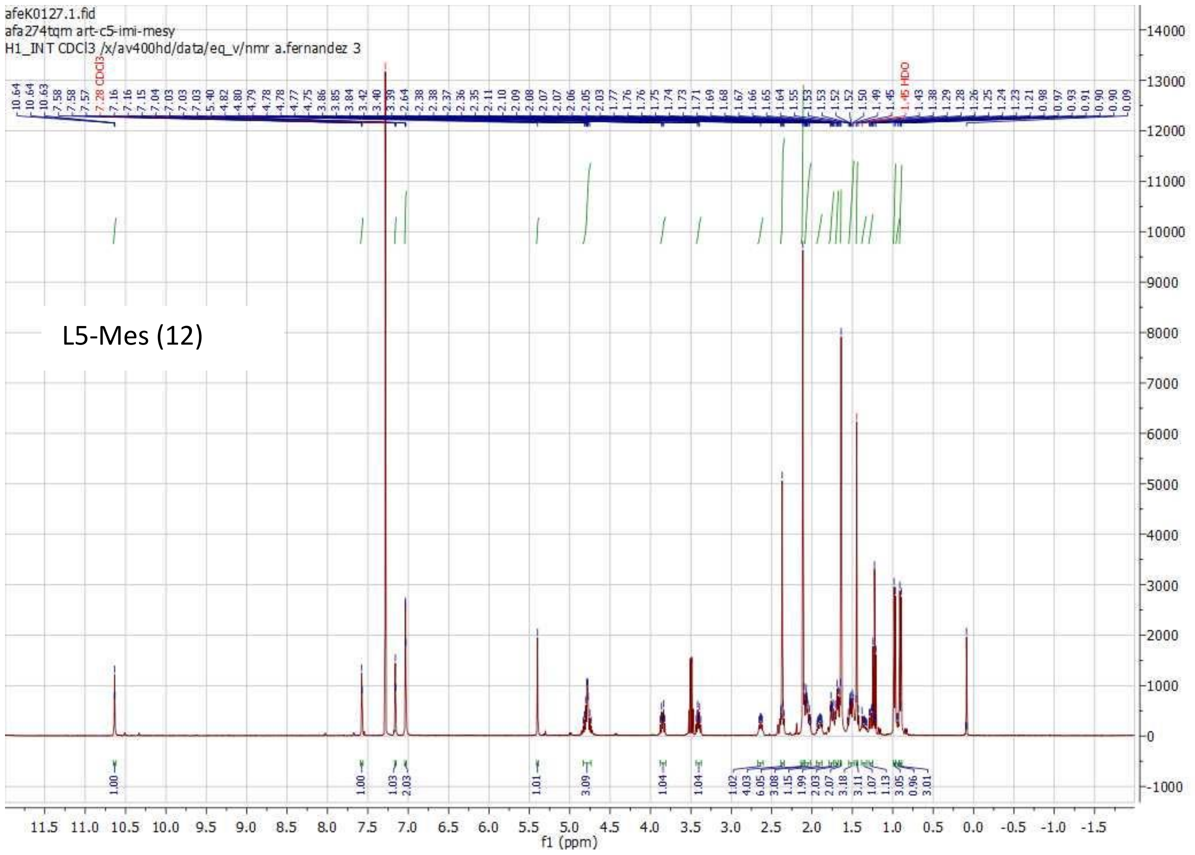
afeK0127.1.fid
afa274tqm art-c5-imi-mesy
H1_INT CDCl3 /x/av400hd/data/eq_v/nmr a.fernandez 3
L5-Mes (12)
f1 (ppm)

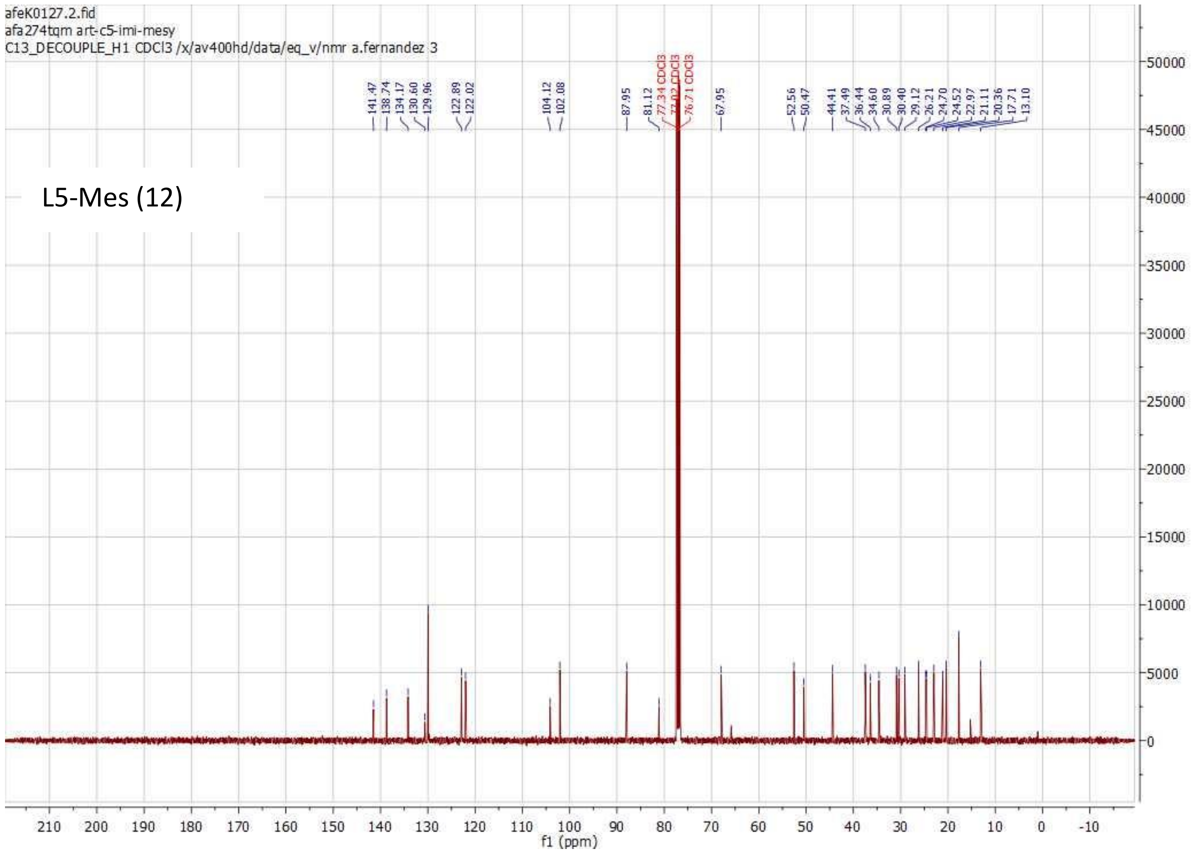
afeK0127.2.fid
afa274tqm art-c5-imi-mesy
C13_DECOUPLE_H1 CDCl3 /x/av400hd/data/eq_v/nmr a.fernandez 3
L5-Mes (12)
f1 (ppm)

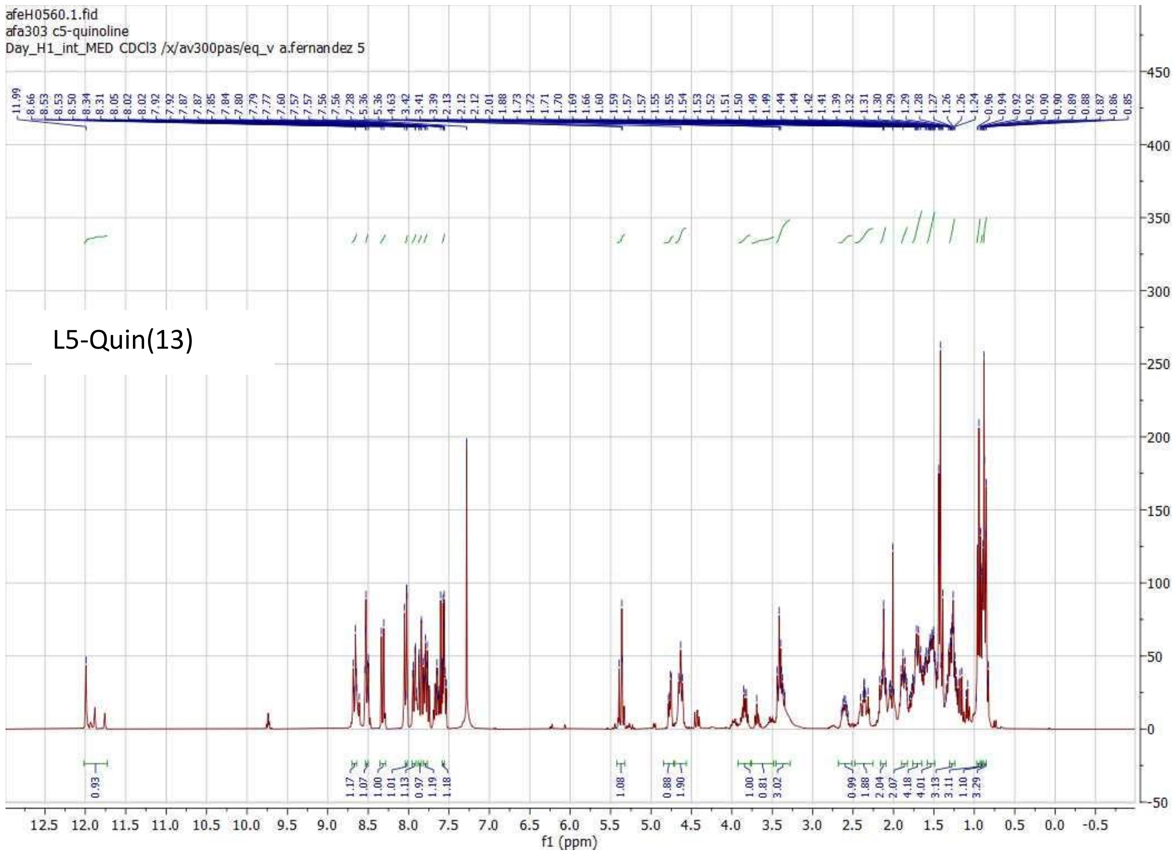
afeH0560.1.fid
afa303 c5-quinoline
Day_H1_int_MED CDCl3 /x/av300pas/eq_v a.fernandez 5
L5-Quin(13)
f1 (ppm)

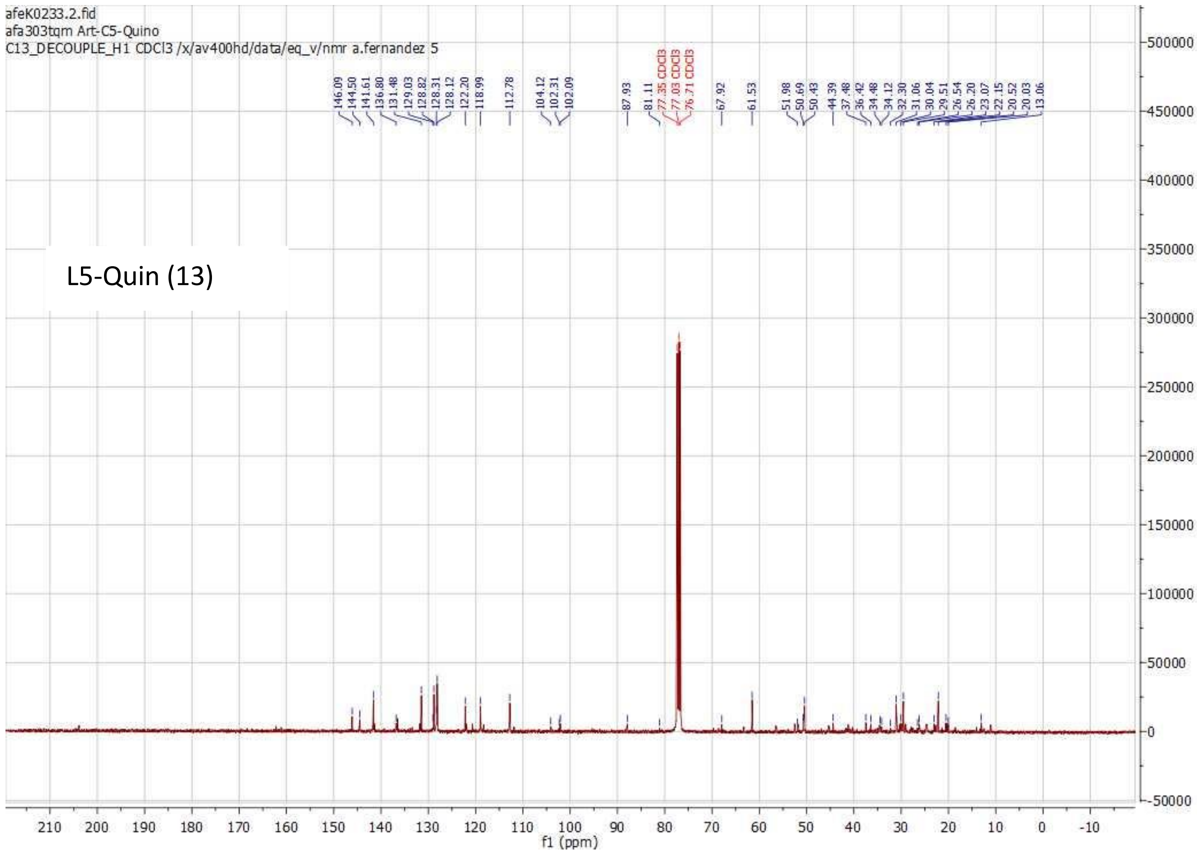
afeK0233.2.fid
afa303tqm Art-C5-Quino
C13_DECOUPLE_H1 CDCl3 /x/av400hd/data/eq_v/nmr a.fernandez 5
L5-Quin (13)
f1 (ppm)

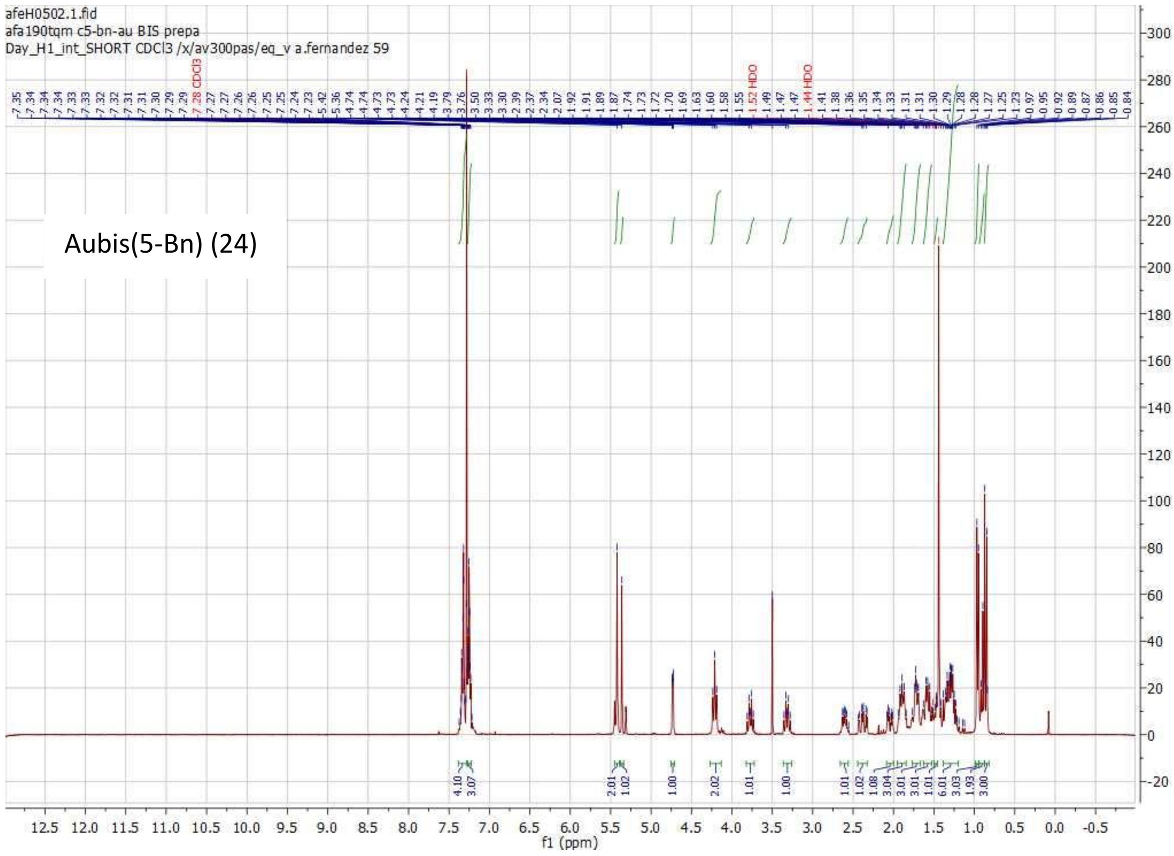
afeH0502.1.fid
afa190tqm c5-bn-au BIS prepa
Day_H1_int_SHORT CDCl3 /x/av300pas/eq_v a.fernandez 59
Aubis(5-Bn) (24)
f1 (ppm)

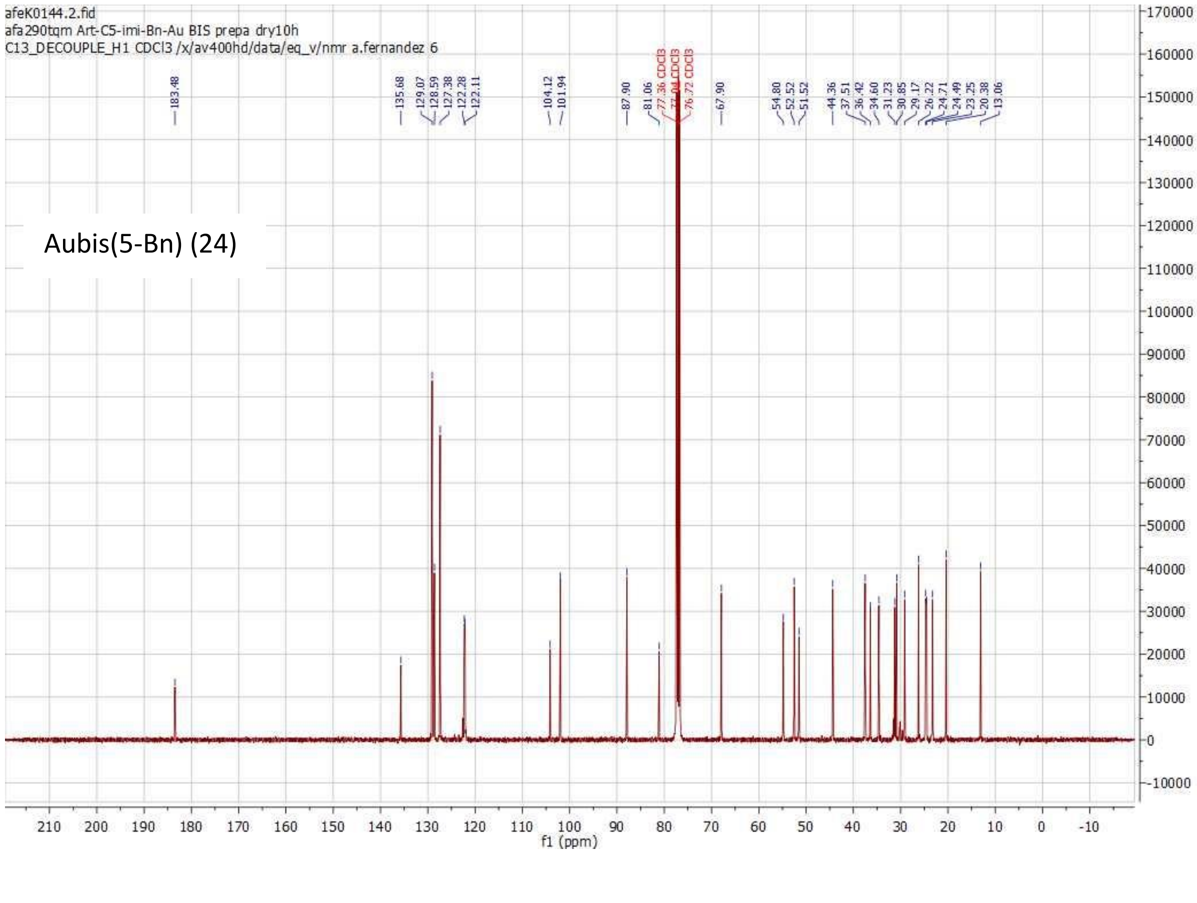
afeK0144.2.fid
afa290tqm Art-C5-imi-Bn-Au BIS prepa dry10h
C13_DECOUPLE_H1 CDCl3 /x/av400hd/data/eq_v/nmr a.fernandez 6
Aubis(5-Bn) (24)
f1 (ppm)

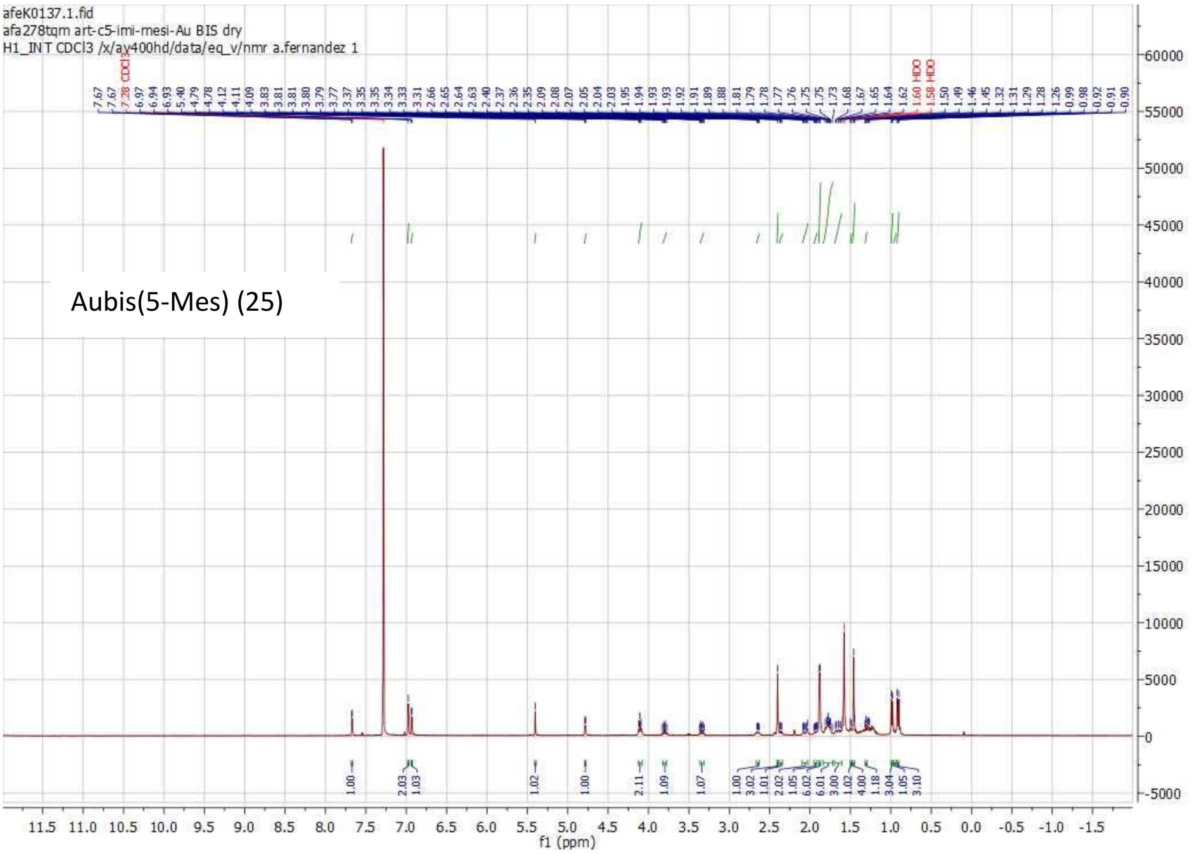
afeK0137.1.fid
afa278tqm art-c5-imi-mesi-Au BIS dry
H1_INT CDCl3 /x/av400hd/data/eq_v/nmr a.fernandez 1
Aubis(5-Mes) (25)
f1 (ppm)

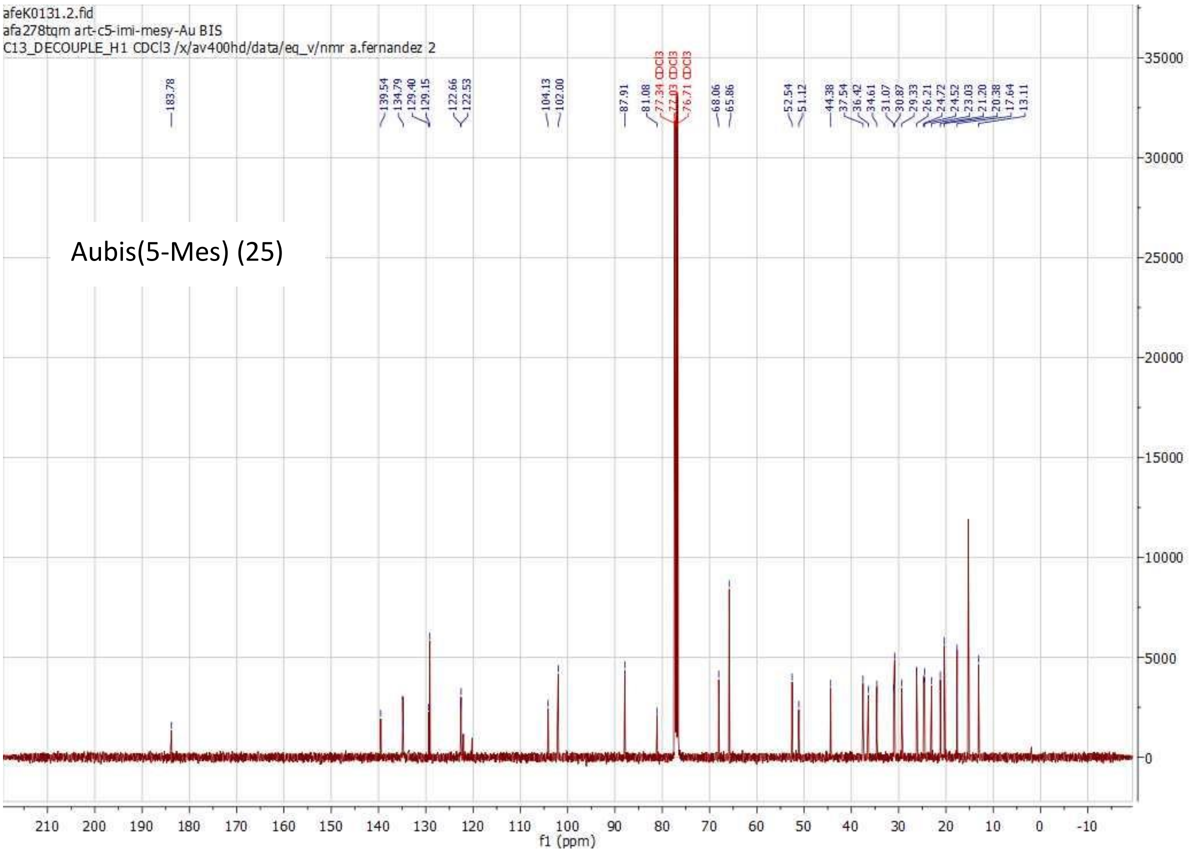
afeK0131.2.fid
afa278tqm art-c5-imi-mesy-Au BIS
C13_DECOUPLE_H1 CDCl3 /x/av400hd/data/eq_v/nmr a.fernandez 2
Aubis(5-Mes) (25)
f1 (ppm)

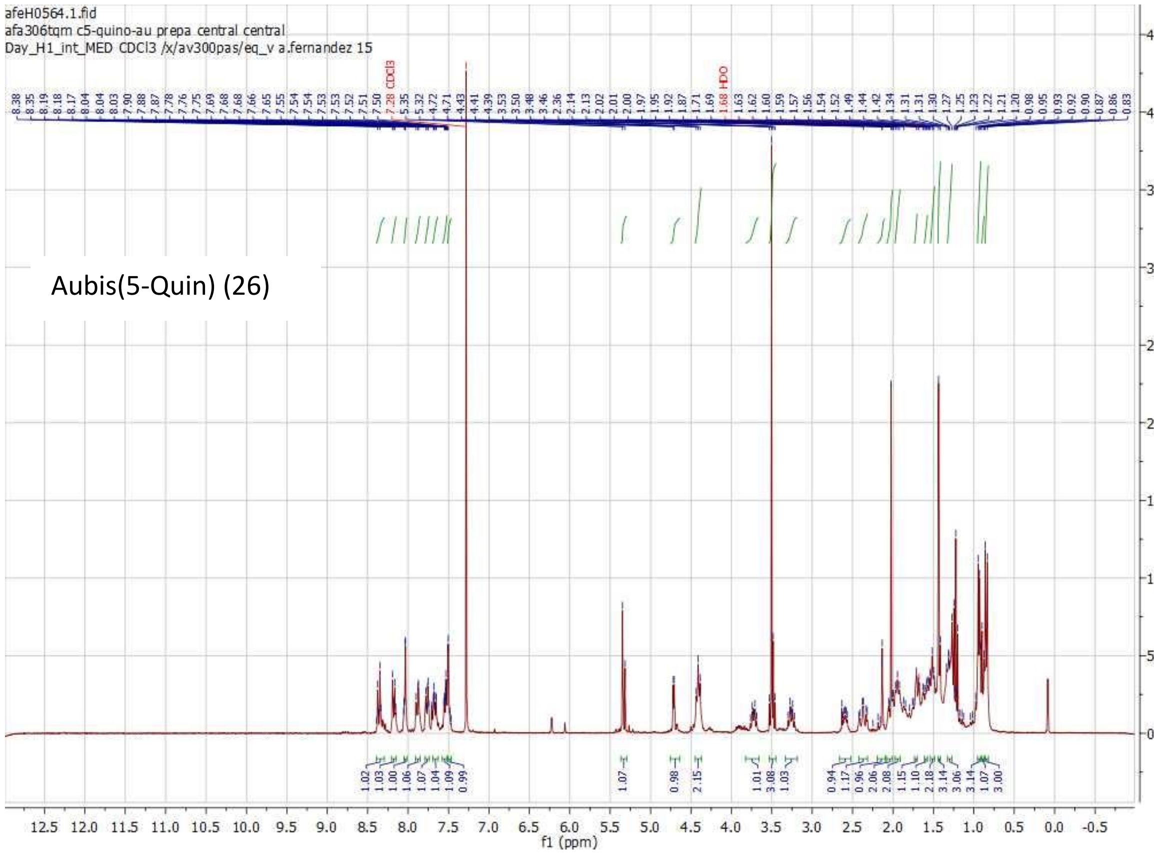
afeH0564.1.fid
afa306tqm c5-quino-au prepa central central
Day_H1_int_MED CDCl3 /x/av300pas/eq_v a.fernandez 15
8.38 8.35 8.19 8.17 8.04 8.03 7.90 7.88 7.87 7.78 7.76 7.75 7.69 7.68 7.66 7.65 7.55 7.54 7.54 7.53 7.52 7.51 7.50 7.28 CDCl3 5.35 5.32 4.72 4.71 4.43 4.41 4.39 3.53 3.50 3.48 3.46 2.36 2.14 2.13 2.02 2.01 2.00 1.97 1.95 1.92 1.87 1.71 1.69 1.68 HDO 1.63 1.62 1.60 1.59 1.57 1.56 1.54 1.52 1.49 1.44 1.42 1.34 1.31 1.31 1.30 1.27 1.25 1.23 1.22 1.21 1.20 0.98 0.95 0.93 0.92 0.90 0.87 0.86 0.83
Aubis(5-Quin) (26)
1.02 1.03 1.00 1.06 1.07 1.04 1.04 1.09 0.99 1.07 0.98 2.15 1.01 3.08 1.03 0.94 1.17 0.96 2.06 2.08 1.15 1.10 2.18 3.14 3.06 3.14 1.07 3.00
f1 (ppm)

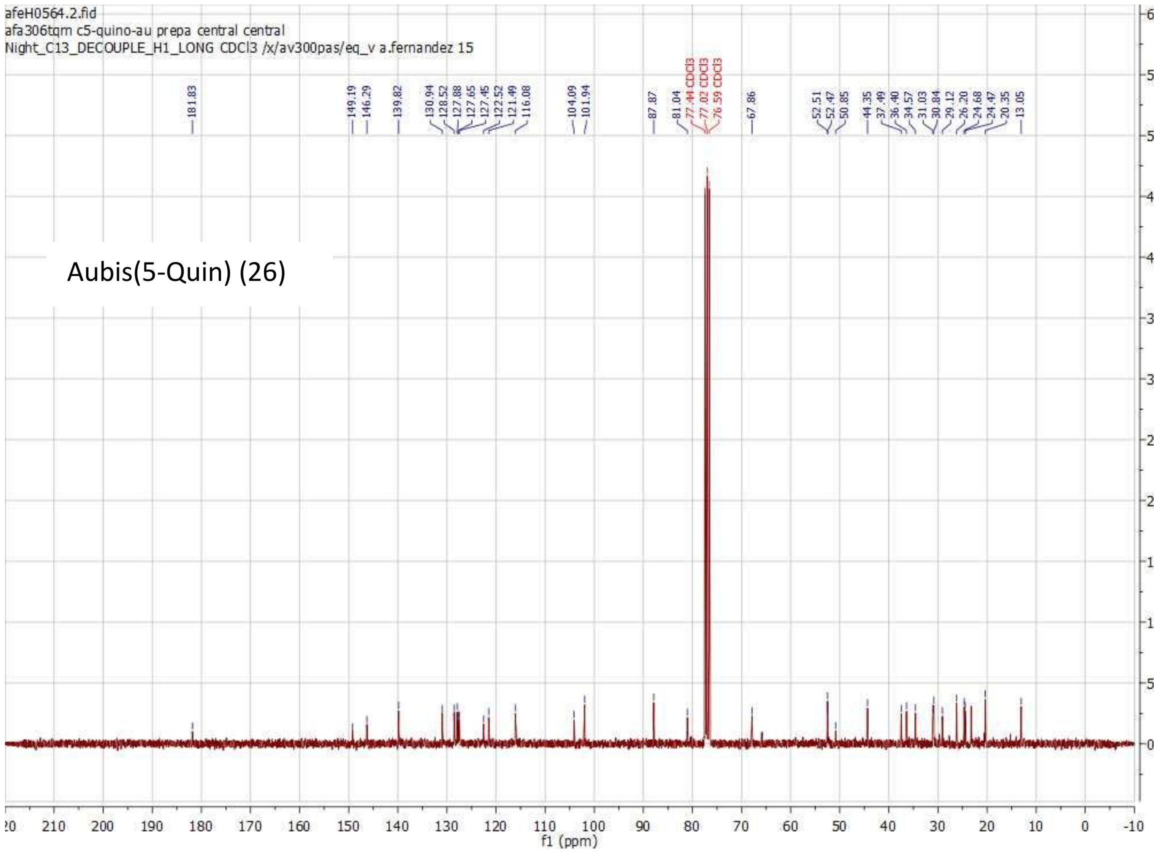
afeH0564.2.fid
afa306tqm c5-quino-au prepa central central
Night_C13_DECOUPLE_H1_LONG CDCl3 /x/av300pas/eq_v a.fernandez 15
181.83 149.19 146.29 139.82 130.94 128.52 127.88 127.65 127.45 122.52 121.49 116.08 104.09 101.94 87.87 81.04 77.44 CDCl3 77.02 CDCl3 76.59 CDCl3 67.86 52.51 52.47 50.85 44.35 37.49 36.40 34.57 31.03 30.84 29.12 26.20 24.68 24.47 20.35 13.05
Aubis(5-Quin) (26)
f1 (ppm)
